# A New Look at Vaccination Behaviors and Intentions: The Case of Influenza

**DOI:** 10.3390/bs15121645

**Published:** 2025-11-30

**Authors:** Valerie F. Reyna, Sarah M. Edelson, David M. N. Garavito, Michelle M. Galindez, Aadya Singh, Julia Fan, Jiwoo Suh

**Affiliations:** Department of Psychology, Cornell University, G331D Martha Van Rensselaer Hall, Ithaca, NY 14850, USA

**Keywords:** vaccine hesitancy, rational choice, influenza vaccination, fuzzy-trace theory, gist, psychological measurement

## Abstract

Although viral outbreaks are increasing, vaccination rates are decreasing. Our aim was to explain this baffling behavior that seems to contradict rational self-interest, and, thus, be beyond the purview of rational choice theories. We integrated fuzzy-trace theory and major theoretical alternatives and applied them to influenza, testing theoretical predictions in two samples: young adults (who are major viral vectors), *N* = 722, and community members, *N* = 185. Controlling for prior knowledge and other psychosocial factors that influence vaccination, explained variance jumped significantly when key predictors from fuzzy-trace theory were added, reaching 62% and 80% for vaccination intentions and 37% and 59% for behavior for each sample, respectively. Single items assessing global gist perceptions of risks and benefits achieved remarkable levels of diagnosticity. Key predictors were intuitive in that they were gisty, imprecise, and non-analytical. In contrast, rational system 2 measures—numeracy and cognitive reflection—were not predictive. These results provide new insights into why individuals vaccinate or not and new avenues for interventions to improve shared clinical decision-making.

Influenza kills more than 500,000 people worldwide annually (Del Riccio et al., 2025). Many more suffer from serious complications from infection (World Health Organization, 2025). Despite scientific evidence that vaccines are safe and effective in reducing mortality and morbidity, vaccination rates remain suboptimal, even among populations with access to the vaccines. For example, in the years since the debut of the COVID-19 pandemic, influenza vaccination rates for adults in the U.S. have steadily declined (Centers for Disease Control and Prevention [CDC], 2024). Drawing on rigorous research and evidence-based theory, we investigated the psychosocial processes that explain and predict vaccination for influenza.

## 1. Background: Prior Theories

Prior approaches to explain and predict vaccination include the health belief model (HBM, developed in the 1950s; Rosenstock, 1974), theory of reasoned action (TRA; Fishbein, 1967), protection motivation theory (PMT; Rogers, 1975), and theory of planned behavior (TPB; Ajzen, 1991). These approaches have been dubbed expectancy-value theories because they assume a kind of psychological calculus of expected outcomes that determines courses of action. That is, the subjective value of an outcome, and the subjective probability or expectation that an action will achieve the outcome, determine behavior—a rational cognitive process (von Neumann & Morgenstern, 1944).

For example, PMT distinguishes between threat appraisal (e.g., perceived susceptibility to and severity of influenza) and coping appraisal (e.g., perceived self-efficacy, ability to access vaccination and response efficacy, belief that vaccination is effective against influenza) to predict taking action to vaccinate. TRA adds consideration of descriptive and injunctive social norms, which are social influences to conform to what important others do and approve of, respectively. TPB adds perceived behavioral control (e.g., perceived ability to obtain and afford vaccines, similar to self-efficacy) to TRA’s construct of attitudes (a positive or negative evaluation of a behavior, e.g., whether vaccination will lead to good or bad outcomes). These theories remain widely used today (e.g., Eberhardt & Ling, 2021; Xu et al., 2025).

Most recently, to expand the focus of expectancy-value models beyond rational cognitive processes, dual-process theories (emotion vs. reason or System 1 vs. 2) have been applied to understand why people do or do not vaccinate (e.g., Tomljenovic & Bubic, 2021). For detailed comparisons of expectancy-value models to fuzzy-trace theory (FTT) and of dual-process models (System 1 and 2) to FTT in the context of health, see Reyna and Farley (2006) and Reyna et al. (2021).

## 2. The Current Approach

All of these theories influenced the approach we take here, but we have substantially modified the explanatory factors in light of FTT’s predictions for vaccination (Reyna, 2012). To wit, variables which originally fell under perceived susceptibility and severity have been revised as global risk perceptions, variables that fell under perceived benefits (including response efficacy) have been revised as global benefits perceptions, and barriers to taking action have been revised as accessibility, which encompasses measures of self-efficacy, perceived behavioral control, and affordability. To preview, the latter combined measure appears to be coherent as it has reliabilities of 0.905 and 0.908 in our two samples.

Also broadly consistent with Rosenstock (1974), we operationalized knowledge using a 51-item objective test that taps knowledge that is relevant to perceived risks and benefits of vaccination. Thus, we agree that “Perceived susceptibility and severity having a strong cognitive component are at least partly dependent on knowledge.” (p. 331). In our approach, raw knowledge is not an end in itself, but a precursor to being able to derive accurate mental representations of the gist of vaccination options (e.g., Garavito et al., 2021; Reyna & Mills, 2014). In addition, our conception of “cognitive” differs fundamentally from prior approaches. Cognition is characterized as emphasizing fuzzy impressionistic thinking that often operates without conscious awareness, assumptions that have been explicitly tested and supported in experimental and mathematical modeling research (e.g., Broniatowski & Reyna, 2018; Reyna et al., 2016).

### 2.1. Fuzzy-Trace Theory

Specifically, according to research on FTT, individuals derive both fuzzy gist and precise verbatim (literal) mental representations in parallel from information and experience, including social media and other inputs. Gist representations capture the bottom-line meaning of inputs at a reduced level of precision, for example, that the risk of vaccination is essentially none (nil), but the benefits are high. Rather than representing a System 1 impulsive process or a mental shortcut, gist is the integrated, meaningful essence of information that supports decision-making.

Therefore, we asked global questions with crude response options that other theories would characterize as vague or ill-formed (e.g., Fishbein, 2008), such as “Overall, for you, which of the following represents the risks of getting a flu vaccine?” where responses were simply none, low, medium, and high. This FTT approach contrasts with the definition of attitudes in TRA, TPB, and similar theories: “Cognitive attitude is elicited by rational evaluations and often measured on a continuum of benefits or losses…whereas affective attitude is evoked by emotions and often measured on a continuum of positive and negative feelings…” (Xiao & Wong, 2020, pp. 5135–5136). Mental representations in FTT can range from categorical (rather than continuous) to ordinal (more precise than categorical) to verbatim that captures continuous quantities. Emotions are not opposed to cognition but, rather, fall out of how inputs are interpreted, namely, the gist (rather than literal facts), and they can reflect deep insights about meaning (Edelson et al., 2024; Reyna, 2021).

Asking questions in a crude but meaningful way (e.g., about global risks and benefits) is also more likely to retrieve gist mental representations of options and of values (values that apply to those options) stored in long-term memory (e.g., Fujita & Han, 2009; Greene et al., 2022). Questions that evoke gist representations cue similar gist memories because of the encoding specificity principle: Retrieval cues are more effective when they match the form that information is stored in memory. Also, because gist representations are more likely to be emphasized over more precise verbatim representations in decision-making—called the “fuzzy-processing preference”—asking questions in a gisty way is more likely to predict decision-making.

This fuzzy processing preference also explains why decisions often boil down to a categorical gist, the least precise form of representation. For example, in the classic standard gamble pitting a sure option against a risky option, most decision makers prefer the sure option because the choices boil down to a categorical gist: gain something for sure or take a chance and either gain something or gain nothing; gaining something is better than gaining nothing, favoring the sure option (Duke et al., 2018; Reyna et al., 2023). Applying the same kind of categorical gist to vaccination decisions, a decision maker who fails to grasp the nature of prevention might view vaccination decisions as boiling down to a choice between a status quo of feeling okay (not vaccinating) versus taking a chance on feeling okay or not feeling okay post vaccination (harms from vaccination). On analogy with the standard gamble, feeling okay is better than (the possibility of) not feeling okay, which would favor not vaccinating. This categorical gist has been found to predict intentions and behaviors in other prevention contexts (e.g., cancer screening, Reyna, 2008; Reyna et al., 2021; Wilhelms et al., 2018). Therefore, we asked participants whether they agreed with various versions of this naïve categorical gist. Note that although gist thinking is more developmentally advanced than verbatim (literal) thinking, and has been associated with healthier outcomes (e.g., Edelson et al., 2024; Reyna et al., 2011; Reyna & Mills, 2014; Wolfe et al., 2015), a gist representation reflects the level of understanding of the individual and thus is not always the ideal representation (e.g., Blalock & Reyna, 2016).

Last among the key FTT ideas, FTT offers a construct that roughly corresponds to social norms in TRA and TPB and updated versions of PMT and HBM. This construct, called “gist principles”, captures social and moral values stored in a simple mental form in long-term memory, such as it is bad to hurt other people or saving lives is good (Reyna, 2021). According to FTT, these principles are cued when the gist representations of decision options remind people of their core values, which can then be applied to options to determine choices.

### 2.2. Additional Constructs and Measures

In addition to being mentally represented in a simple generic form, gist principles are unlike social norms to the extent that the latter are about conformity and other social pressures (that can be exerted counter-attitudinally) regardless of whether the values they represent have become internalized. Nevertheless, we agree that social norms can affect behavior in ways that go beyond internalized simple values through pressures to conform to perceived descriptive norms or to seek approval of important others through adherence to perceived injunctive norms. Therefore, we administered such measures and compared their predictive validity to the FTT predictors to assess empirically whether they accounted for additional variance in vaccination intentions or behavior.

To contrast with our global measures of the gist of risk, we also administered fine-grained measures of perceived risk, namely, quantitative assessments of specific adverse outcomes on a 0 to 100 scale. No measure is process-pure, but the fine-grained assessment is more likely to tap verbatim representations about specific details (to the extent that they have been encoded and can be retrieved) compared to the global gist measure, which is more likely to tap gist representations of risk. Other FTT research has shown that the variance in corresponding (same person, same action or event, but different way of asking about perceived risk) verbatim and gist measures of risk perception can be disentangled from one another, and they can be doubly dissociated (e.g., correlations in opposite directions with third variables; Mills et al., 2008; Reyna et al., 2011). Therefore, we conducted analyses to address the issue of whether the gist and verbatim measures of risk perception are distinct in the context of vaccination.

Completing our panoramic comparison of theories, we also compared FTT predictors to dual-process predictors, such as numeracy and cognitive reflection. Dual-process theories make the straightforward prediction that individuals high in the ability to use and understand numbers (E. Peters, 2020) and/or high in cognitive reflection (System 2 reasoning, Kahneman, 2011) should be more likely to intend to vaccinate and to follow through on those intentions (Murphy et al., 2021; follow-through because of higher executive functions such as planning; e.g., Thoma et al., 2021). Prior research has shown that these variables (numeracy and cognitive reflection) can predict behavior (Martinelli & Veltri, 2021), but effects are sometimes the opposite of those anticipated by dual-process theory (Reyna et al., 2025). In any case, we analyze them here to show that these constructs are distinct from imprecise, non-reflective, and adaptive gist thinking.

Our results also allow us to gain purchase on three additional issues: We discuss how our measures solve the commensurability problem of comparing the relative strengths of perceived risks and benefits in determining intentions and behavior, the greater impact of benefits over risks on vaccination intentions and behavior (which is often neglected in public health messaging), and the diagnostic value of asking two simple questions about global risks and benefits to identify individuals with misconceptions about vaccination without directly asking about vaccine hesitancy.

## 3. Method

### Participants

We recruited a young adult sample of college students (*N* = 722, ranging in age from 18 to 28 years old, *M* = 19.52) who participated to fulfill a course requirement or receive extra credit. Young adults were recruited because they represent major vectors of disease (National Academies of Sciences, Engineering, and Medicine, 2020). Although younger adults are less likely to experience serious complications or death from influenza, they are more likely to spread the virus compared to older adults (McGovern et al., 2024; T. R. Peters et al., 2014). Influenza spreads rapidly in university environments where large numbers of individuals are in close proximity and interact widely (e.g., in large classes and dormitories housing large numbers of students). Thus, vaccinating young people can be effective in preventing the spread of influenza to vulnerable populations (Taksler et al., 2015). Two young adults completed all but one of the study measures. An additional 92 respondents were excluded for failure to provide consent or incomplete data (none of which could be included in our main regression analyses due to listwise deletion). Demographics for the young adults were 60% White, 29% Asian, 7% Black, and 4% mixed/other; 70% were female, and 11% were Hispanic (asked separately from race).

A community sample (*N* = 185, ranging in age from 20 to 76 years old, *M* = 39.72) was recruited using a combination of snowball sampling from outreach and extension community networks and social media outreach to capture a variety of viewpoints, including online communities expressing vaccine hesitancy. Twenty-five participants declined to provide demographic information. Another 51 participants had partial data and are included in analyses when possible; see Supplemental Materials. Fifty-three individuals were excluded for failure to consent or advance past the first question of the survey. Demographics for the community members were 82% White, 8% Asian, 4% Black, and 7% mixed/other. Similar to the young adults, community members were 71% female, and 10% Hispanic (asked separately from race).

Twenty-two percent of community members completed high school and the remaining completed higher education. The community sample varied in terms of occupation: 8% in services (e.g., food, sales, etc.), 12% staff or aides, 13% management, 9% healthcare professionals, 23% other trained or licensed professionals, 9% self-employed, 9% retired/out of the workforce, and 18% other. Substantial numbers in both samples were not vaccinated; additional descriptive statistics for the two samples are shown in Table 1.

## 4. Materials

Predictors of vaccination intentions and behavior and the latter outcome measures are described below. Two scales were administered only in one sample: Based on prior research, we expected that social norms regarding vaccination—the behavior and approval of friends and other important adults (e.g., parents)—would be important for young adults, and so we asked those questions for the young adult sample (e.g., Albert et al., 2013). Prior research also suggests that older people are less likely to be high in numeracy, so we administered the Subjective Numeracy Scale and the Cognitive Reflection Test (that taps both numeracy and reflection) to the community sample (Fagerlin et al., 2007; Meyer et al., 2024; Reyna & Brainerd, 2007).

### 4.1. Predictors of Vaccination Intentions and Behavior

**Global Risks of Getting the Flu Vaccine.** A single item measured participants’ overall, global gist assessment of the risks associated with the flu vaccine: “Overall, for YOU which of the following best represents the RISKS of getting a flu vaccine?” Responses were *none*, *low*, *medium*, and *high* (scored as 1–4).

**Global Benefits of Getting the Flu Vaccine.** A single item measured participants’ overall, global gist assessment of the benefits associated with the flu vaccine: “Overall, for YOU which of the following best represents the BENEFITS of getting a flu vaccine?” Responses were *none*, *low*, *medium*, and *high* (scored as 1–4).

**Accessibility.** A 4-item scale was developed to measure self-efficacy, perceived behavioral control, and affordability (e.g., “I would find it difficult to obtain a flu vaccine,” reverse scored; see Supplemental Materials). Participants responded on a 5-point Likert scale: *completely disagree*, *disagree*, *neither disagree nor agree*, *agree*, and *completely agree* (scored as 1–5). Higher scores indicated greater perceived access to the flu vaccine. The reliability of this scale was excellent (α = 0.905 and 0.908 for young adults and community members, respectively).

**Vaccination Knowledge.** A 51-item scale measured knowledge of scientific and practical facts about influenza and the vaccine that is relevant to perceived risks and benefits about vaccination (e.g., “Getting the vaccine will weaken my immune system and make me more susceptible to other illnesses,” reverse scored; see Supplemental Materials). Participants responded on a 5-point Likert scale: *false*, *probably false*, *could either be true or false*, *probably true*, *and true* (scored as 1–5). Knowledge ratings were computed as a mean of the 51 items, where higher scores indicate endorsement that true items are true and false items are false (reverse scored), and as proportion of items strictly correct, where *probably true* and *true* were counted as correct for true items and vice versa for false items and scored as +1; *could either be true or false* was scored as 0; and incorrect items were scored as −1. Reliability of this scale was good for both methods of scoring (for young adults, α = 0.887 and 0.845, and for community members, α = 0.935 and 0.919 for rating and correct, respectively).

**Status Quo**. A 3-item scale measured an uninformed application of categorical thinking to flu vaccination: conceiving of the choice as feeling okay versus taking a chance on vaccinating (e.g., “I feel fine now, so it is unnecessary to get the flu vaccine”). Participants responded on a 5-point Likert scale: *completely disagree*, *disagree*, *neither disagree nor agree*, *agree*, and *completely agree* (scored as 1–5). This scale had good reliability in both samples (α = 0.852 and 0.819 for the young adult and the community sample, respectively).

**Gist Principles of Social Responsibility**. An 11-item scale measured participants’ endorsement of bottom-line principles related to the social responsibility of vaccination stored in a simple mental form in long-term memory (e.g., “You should not hurt other people by giving them the flu;” see Supplemental Materials). Higher scores indicated that participants agreed with social and moral values having to do with the responsibility to protect people from harm by vaccinating. Participants responded on a 5-point Likert scale: *completely disagree*, *disagree*, *neither disagree nor agree*, *agree*, and *completely agree* (scored as 1–5). This scale had excellent reliability (α = 0.909 and 0.961 for young adults and community members, respectively).

**Social Norms.** Four items assessed social influences to conform to perceived descriptive norms (what others are doing) or to perceived injunctive norms (what others believe ought to be done). Two descriptive norm items were administered: “Most of my friends have gotten the flu vaccination” and “Most adults who are important to me have gotten the flu vaccination.” Descriptive norms of vaccination behavior for others and self-reported behavior (below) were both scored 1 for *yes* and otherwise 0 for *no* or *I don’t know*. Two injunctive norm items were administered: “Most of my friends believe people should get a flu vaccine” and “Most adults who are important to me believe people should get a flu vaccine.” For injunctive norms, similar to gist principles, participants responded on a 5-point Likert scale: *completely disagree*, *disagree*, *neither disagree nor agree*, *agree*, and *completely agree* (scored as 1–5).

**Quantitative Risk of Getting Sick from the Flu Vaccine.** An 11-item scale was used to measure individuals’ perceived precise level of specific risks of the influenza vaccine, including adverse outcomes such as death, side effects, and other potential risks of vaccination (e.g., “Probability of having a negative reaction to the flu vaccine?”; see Supplemental Materials). Participants indicated their risk perception from 0% to 100%. This scale had excellent reliability (α = 0.907 and 0.900 for young adults and community members, respectively).

**Subjective Numeracy Scale.** The 8-item Subjective Numeracy Scale (SNS; Fagerlin et al., 2007) was administered to measure self-reported numerical ability, a scale extensively used in prior research (e.g., “How good are you at working with fractions?”). Reliability for this scale was good (α = 0.858 for community members).

**Cognitive Reflection Test**. The 3-item Cognitive Reflection Test (CRT; Frederick, 2005; Meyer et al., 2018) was administered to measure System 2 analytical thinking, extensively used in prior research. Correct responses were scored as 1 and incorrect as 0.

### 4.2. Outcome Measures

**Intentions to Get Vaccinated.** A 6-item scale measured participants’ intentions to receive the flu vaccine this flu season, next flu season, every flu season, most flu seasons, and at least once in one’s lifetime (e.g., “Do you intend to get a flu vaccine at the start of the next flu season?”; see Supplemental Materials). Participants responded on a 5-point Likert scale: *extremely unlikely*, *unlikely*, *undecided*, *likely*, and *extremely likely* (scored 1–5). This scale had excellent reliability (α = 0.944 and α = 0.974 for young adults and community members, respectively).

**Past Year Vaccination Behavior**. A single item measured past year vaccination behavior: “In the past year have you had a flu vaccination (i.e., a shot, spray, drop or mist)?” A *yes* response was scored 1, otherwise 0 for *no* or *I don’t know*.^1^

## 5. Procedure

After providing informed consent, participants responded to the survey materials as follows: past year vaccination behavior followed by vaccination knowledge, social norms (young adult sample), status quo gist, gist principles of social responsibility, intentions to get vaccinated, accessibility, quantitative risk of getting sick from the flu vaccination, global risks and benefits of getting the flu vaccine, subjective numerary scale and Cognitive Reflection Test (for community sample), and, finally, demographics.

## 6. Results

### 6.1. Descriptive Results

Table 1 displays descriptive statistics for predictors and outcomes. Regarding background variables, knowledge of basic facts reached only 0.434 correct in the young adult sample and 0.667 correct in the community sample. Most reported that the vaccine was affordable, and that they had sufficient self-efficacy and personal behavioral control to obtain a vaccine. Regarding key theoretical variables, a surprising number of respondents in both samples did not reject the status quo gist, though they disagreed on average. Both groups tended to endorse the gist principles of social responsibility. On average, both groups perceived the global benefits of vaccination as medium (about 3 on a 1–4 scale) and the global risks of vaccination as low (about 2 on a 1–4 scale), with benefits-to-risks ratios of 1.51 for the young adult sample and 1.74 for the community sample, favoring vaccination but falling short of the most favorable ratio of 4.00 (4 to 1). The estimated quantitative risks of harms from the vaccine on a 0–100% scale were highly similar in the young adult (16%) and community samples (18%).

Slightly less than half of the young adults reported that their friends and other adults important to them were vaccinated, and they mostly agreed that they would approve of vaccinating. The community sample rated themselves as fairly high on subjective numeracy (78% of the maximum rating of 6) and correctly answered 61% of the CRT items (similar to a recent college sample of young adults; Reyna et al., 2025). Substantial variation remained in the measures to produce highly reliable scales and to observe robust correlations with vaccination intentions and behavior, as discussed below.

### 6.2. Bivariate Correlations

Beginning with the demographics in the young adult sample, women had slightly but significantly higher intentions to vaccinate compared to men (r_pb_, point biserial correlation = 0.101, though behavior was non-significant), as did those who were White compared to non-White (r_pb_ = 0.145). Black/African Americans had significantly lower intentions (r_pb_ = −0.150) compared to those who were not Black/African American. Similar results for race were observed for self-reported vaccination behavior; see Supplemental Materials. Age (as expected in this young sample), being Hispanic, and being Asian were not related to intentions or behavior. In the community sample, none of the demographic variables was significant, except for age, which correlated positively with both intentions and behavior (see Supplemental Materials).

Regarding the explanatory variables, we begin with the background variables of accessibility and knowledge, both of which correlated in expected directions such that greater accessibility and higher knowledge were associated with increased vaccination intentions and behavior. Specifically, in the young adult sample, accessibility and knowledge correlated significantly with both intentions and behavior, with knowledge (r’s = 0.361 and 0.181, respectively) exceeding accessibility (r’s = 0.249 and 0.124, respectively; see Supplemental Materials). In the community sample, the corresponding four correlations were also significant, but the magnitudes for knowledge jumped to 0.739 and 0.479 for intentions and behaviors, respectively. Notably, accessibility correlated higher with behavior (r = 0.291) than with intentions (r = 0.189) in the community sample, suggesting that access was a barrier to acting on intentions for some community members. SNS and CRT did not correlate significantly with either intentions or with behavior.

Regarding the key theoretical variables, all of them correlated significantly with intentions and behavior, including all FTT variables as well as social norms (see Supplemental Materials). As examples, in the young adult sample, gist principles and global benefits correlated positively and substantially with intentions (r’s = 0.681 and 0.571, respectively), whereas status quo gist and global risks correlated negatively (r’s = −0.567 and −0.233, respectively). Similarly, but even more strongly, in the community sample, the corresponding correlations with intentions were r = 0.836 (gist principles) and r = 0.819 (global benefits) versus r = −0.753 (status quo gist) and r = −0.450 (global risks). The correlations with behavior were lower, but all eight (four for each sample) were significant (see Supplemental Materials).

The question that we now turn to is, given the significant associations between these variables and important outcomes, do the key FTT explanatory variables each predict unique variance beyond the background variables, that is, beyond demographics, accessibility, and knowledge, as well as social norms, SNS, and CRT.

### 6.3. Hierarchical Multiple Regressions

We tested a series of nested regression models to predict vaccination intentions (linear regression) and behavior (logistic regression) for each sample (see Table 2 and Table 3, respectively, and Table 4 and Table 5, respectively). In the young adult sample, Model 1 contained demographics and background variables that accounted for small but significant variance in intentions, R^2^ = 0.144, and in behavior, R^2^ = 0.053. Consistent with the bivariate correlations, race and knowledge were significant predictors of intentions, and knowledge was a significant predictor of behavior, controlling for age, sex, race/ethnicity, and accessibility.

Adding the FTT predictors in Model 2 in the young adult sample, the variance accounted for jumped significantly to 0.581 for intentions and 0.319 for behavior. Each of the four key theoretical predictors accounted for unique variance for both intentions and behavior, controlling for demographics, other background variables, and quantitative risk perception: Endorsement of gist principles and perceived global benefits were associated with higher intentions to vaccinate and greater likelihood of vaccinating, whereas status quo gist and global risk were associated with lower intentions to vaccinate and less likelihood of vaccinating.

Moving to Model 3 in the young adult sample, we added the four social norm items separately to the Model 2 predictors. Adding each item separately produced higher (or very similar) beta weights and odds ratios, compared to combining them (see Supplemental Materials for alternative analyses). For all social norm analyses of intentions, Model 3 produced an additional significant but small gain in R^2^ to 0.586 for friends—descriptive norms, to 0.604 for adults—descriptive norms, to 0.589 for friends—injunctive norms, and to 0.622 for adults—injunctive norms (all increases in R^2^ were significant). The corresponding variance accounted for in behavior was 0.366, 0.360, 0.328, and 0.349 (all increases in R^2^ were significant).

Because quantitative risk had a small but significant reversal of sign in Model 2 (positive) compared to bivariate correlations with intentions (negative), we also evaluated Model 2 omitting quantitative risk; the results for the other four FTT predictors remained highly similar and significant (see Supplemental Materials). The same was true for the logistic regression for which the four FTT predictors remained similar and significant when quantitative risk was omitted (see Supplemental Materials). Knowledge also reversed sign in Model 2 compared to the bivariate correlation and compared to Model 1 results; again, omitting that variable produced highly similar results for the remaining four FTT predictors (see Supplemental Materials).

Turning to the community sample, for Model 1, similar to the young adult sample, knowledge was a significant predictor of both intentions (Table 4) and behavior (Table 5). However, accessibility also accounted for unique variance in behavior in Model 1. (Accessibility remained a consistently positive predictor of behavior in the community sample for all three models.) Model 1 accounted for a substantial R^2^ of 0.574 in variance for intentions, which jumped to 0.800 for Model 2 (a significant increase) when the FTT predictors were added, but variance did not significantly increase in Model 3 when SNS was added as a predictor.

In Model 2 and Model 3 for the community sample, the FTT predictors of gist principles and global benefits were significant positive predictors of intentions and behavior, and agreement with the status quo gist was a negative predictor of intentions. Global risk reached significance when demographic variables were excluded for both Model 2 and Model 3 for intentions, but not for behavior in either Model 2 or 3 (see Supplemental Materials). The variance accounted for in behavior was 0.417 for Model 1, and this increased significantly to 0.582 in Model 2; the variance of 0.586 in Model 3, when SNS was added, did not differ significantly from Model 2. Quantitative risk did not reach significance for the community sample in any of the models tested.

Although the nested models we have just reviewed answer questions about predictive power controlling for other variables, they do not address the degree to which the FTT explanatory variables are sufficient by themselves (or are an artifact of controlling for other variables) to predict intentions and behaviors. Additional regressions containing only the FTT variables showed that they accounted for the lion’s share of the variance in intentions (0.571) and behavior (0.299) for young adults, as shown in Table 6, Panel A, and for intentions (0.786) and behavior (0.501) in the community sample, as shown in Table 6, Panel B (see Supplemental Materials for additional details).

### 6.4. Diagnosticity of Global Benefits and Risks for Vaccine Hesitancy

Because global benefits and global risks are assessed on the same scale with interpretable response options, they can be meaningfully combined. The empirical question is whether perceiving higher global benefits and/or lower global risks would be diagnostic of having vaccinated or intending to vaccinate. To evaluate the diagnosticity of combining these two simple questions for vaccine behaviors and intentions, we defined vaccine favorable as perceiving global benefits of medium or high and global risks of none or low; vaccine hesitant was defined as perceiving benefits as none or low or risks as medium or high. We designated the former as a positive test result and the latter as a negative test result. For the purposes of assessment, we dichotomized the intentions scale in a straightforward way such that low intentions fell below the midpoint of ratings (below *undecided*) and high intentions fell above the midpoint (above *undecided*). This scoring excluded 86 young adults and 7 community members who did not indicate a clear intention about influenza vaccination. Behavior was already scored dichotomously.

In the young adult sample, for behavior, these criteria yielded a sensitivity of 78%, a specificity of 48%, a positive predictive value of 65%, and a negative predictive value of 65%. Predicting intentions among young adults yielded higher performance with a sensitivity of 82%, a specificity of 57%, a positive predictive value of 82%, and a negative predictive value of 57%. In the community sample, for behavior, these criteria performed remarkably well with a sensitivity of 92%, a specificity of 58%, a positive predictive value of 76%, and a negative predictive value of 83%. These criteria achieved the highest performance for intentions in the community sample, with a sensitivity of 93%, a specificity of 85%, a positive predictive value of 95%, and a negative predictive value of 82%. Thus, two simple questions with simple response options and simple scoring (dichotomized) were able to predict vaccine favorability/hesitancy at levels well above chance, comparable to some clinical screening tests.

Table 7 integrates the results we have discussed with predictions of alternative theories, contrasting fuzzy-trace theory and expectancy value models.

## 7. Discussion

Socioeconomic factors influence vaccination behaviors. Individuals and countries with higher economic resources and higher levels of education have better access to healthcare, including vaccination, and these factors are not distributed randomly across racial and ethnic groups (Liu et al., 2024). Thus, it is not surprising that prior research has shown that demographics are linked to accessibility—but accessibility goes beyond demographics. In this study, measures of perceived ability to access vaccination (self-efficacy), of perceived behavioral control over access, and of economic barriers to access hung together tightly in our samples and accounted for unique variance beyond demographics in explaining whether community members had vaccinated.

However, the largest vaccination problem facing many societies today is not access so much as it is attitude. Declining vaccination rates even for less controversial vaccines, such as those for influenza, represent a growing threat to public health. The purpose of this work was to explain this baffling ideation about vaccination that seems to contradict rational self-interest, and, therefore, to be beyond the purview of rational choice theories.

Rational choice theories often begin with knowledge, and knowledge was strongly related to behavior and intentions in both samples in bivariate correlations (without partialling out shared variance with any other variables) and in baseline models. However, once theoretically motivated predictors that are predicated on knowledge were entered into equations, controlling for knowledge and all other background variables, the variance that the models could explain jumped substantially: Simple core values, such as it is not right to hurt other people, strongly predicted whether people of different ages, sexes, races, ethnicities, knowledge, and beliefs about accessibility intended to vaccinate or had vaccinated.

Conversely, endorsing a status quo gist that feeling okay means that it is unnecessary to vaccinate (and take on the possibility of harm by vaccinating) predicted that people of all backgrounds were less likely to intend to vaccinate or be vaccinated. The degree to which this naïve gist was endorsed in these samples, and that it significantly predicted lower intentions and less vaccination, reveals a breathtaking lack of insight into the nature of prevention. Vaccinations are useful precisely when people feel fine. Endorsing such a gist goes beyond not knowing the difference between true and false facts about prevention, as controlling for knowledge and still obtaining this result illustrates.

Global benefits of vaccination, measured with a single item that had gisty response categories (none, low, medium, and high), also consistently predicted whether people intended to vaccinate or had vaccinated across samples. Global benefits carried more weight than global risks in vaccination intentions and behavior (for similar asymmetries, see Reyna et al., 2011; Reyna & Farley, 2006): Perceived benefits were rated higher than perceived risks, and correlations with outcomes (intentions and behaviors) were also substantially greater for benefits than risks. Although public health messaging often focuses on risks, these results suggest that focusing on benefits (which predict behavior independently and in addition to risks) might be more effective.

We can make these comparisons because global benefits and risks are on the same meaningful scale and involve a global question in which only the words “benefits” and “risks” differ. Importantly, most theories approach measuring perceived benefits and risks by assessing perceptions of multiple specific conditions and consequences, which cannot be directly compared with one another. We are aware that such measures can be normed and that standardized scale scores can be compared, but their interpretation then rests on dubious assumptions about comparability across items, normality of distributions, and how different dimensions of conditions or outcomes should be weighted and combined. Asking for numerical responses does not solve these problems, and, moreover, numbers are ambiguous; it is the interpretation of numbers as high or low that drives decisions (Reyna & Brainerd, 2023). The simple global gist questions avoid these complexities by asking the individual to do the weighting and combining intuitively with minimal scoring assumptions (i.e., monotonicity).

According to prior theories, answering such unspecified global questions about benefits and risks should produce unreliable answers (e.g., Fishbein, 2008). After all, it is unlikely that individuals take a mental inventory of all of the benefits and risks that they are aware of in the few seconds it takes to make these judgments. For example, research on unpacking effects in questions about risk and probability demonstrates that examples are not necessarily retrieved when people are asked more encompassing global as opposed to more specific questions (Sloman et al., 2004; Reyna & Adam, 2003). The results here, that quantitative risk questions about specific behaviors (behaviors that individuals readily acknowledge are included in global questions) show different patterns than global risk questions, confirm that global and specific questions are not interchangeable psychologically (Mills et al., 2008).

We argue that individuals are able to take an intuitive inner glance at their perceptions of the gist of benefits and risks of vaccination and come up with valid and reliable responses because they have stored this gist as part of their experiences and knowledge. Retrieval cues in the question also matter, as FTT predicts. Our results indicate that when there are fuzzy impressions of the gist of benefits and risk stored in long-term memory, they can be accessed rapidly and non-deliberatively (e.g., without accessing a series of specific examples and weighing them) with simple global questions.

Thus, when benefits are perceived as medium or high and risks are perceived as none or low, most of the time, individuals have favorable intentions about vaccinating that can then be facilitated, if they are not already vaccinated (e.g., by providing easy access). Conversely, when individuals perceive benefits as none or low or risks as medium or high, most of the time, they are vaccine-hesitant. Further, like an echocardiogram, the test can also diagnose the nature of the problem. The answers to the two questions make it possible to open a dialog and to target communicating the scientifically supported gist, focusing on misconceptions about benefits, risks, or both, supporting shared clinical decision-making (e.g., Brace & Wolfe, 2024). These two simple yet encompassing questions could be folded easily into primary care or public health clinics to flag patients who need counseling about vaccination and what they need counseling about.

Social norms were also assessed with single items that proved remarkably robust at predicting intentions and behaviors, independently of FTT’s psychosocial predictors. Hierarchical modeling showed that social norms added variance controlling for all other predictors, suggesting that social influences or pressures can override individuals’ beliefs, attitudes, or preferences. However, there was no evidence of System 2 override (i.e., higher order cognition inhibiting irrational impulses or guiding intentions and behaviors) in that numeracy did not account for unique variance and cognitive reflection did not correlate with intentions or behavior. We do not argue that such factors are never important but that gist-based predictors tend to play a primary role in explaining vaccination intentions and behavior due to the fuzzy-processing preference.

Given that the items that mainly predicted intentions were intuitive in the sense that they were gisty, imprecise, and not analytical or reflective, it might seem odd that they predicted what seems like an analytical or reflective construct, namely, intentions. Some intentions are the product of planning and analysis, drawing on System 2 executive processes. However, asking individuals about their intentions might encourage them to form a judgment in that moment to answer the question rather than to report a previously formulated intention. Thus, intentions are not necessarily intentional in the sense of being premeditated and planful—they could represent a moment in time’s judgment in response to a cue or question.

Limitations of this study include that the samples were not nationally representative, although the goal of the research was explanatory, not descriptive. Nevertheless, it would be useful to know how widespread these perceptions and attitudes are in the population. Finding these levels of troubling perceptions, attitudes, and behaviors in college students and relatively educated samples should raise the alarm about how prevalent they might be in the population at large. Also, the study is mainly correlational rather than causal, but, again, the goal of this research was to explain rather than change ideation. However, see Blalock and Reyna (2016) and Reyna et al. (2022) for reviews of experiments on gist-based change in health-related ideations and associated behaviors, based on FTT. Such prior work corroborates causal conclusions. While it is possible that performing a behavior or forming an intention changes perceptions of risks and benefits, those effects are generally weaker than the opposite relation (perceptions of risks and benefits causing behavior and intentions), but these causal relations should be explored further in this context. In addition, we did directly contrast manipulations of questions, notably, precise quantitative versus global categorical judgments of risk, finding distinct patterns and more robust results with the global measures. Future studies should further compare measures of specific and quantitative perceptions versus global gist perceptions of dimensions such as vaccine effectiveness and the risk of getting the flu.

Taken together, our results suggest a new theoretical perspective that differs from dominant perspectives. FTT introduces a new class of gist-based predictors (status quo gist, gist principles, global risk, and global benefit) that explained a major proportion of the variance for vaccination intentions and behavior. Moreover, these gist-based predictors remained significant controlling for important predictors present in other theories—accessibility, knowledge, conformity to social norms, quantitative risk, and numeracy. The gist-based explanatory factors we have identified provide insight into why individuals choose to vaccinate or not. These decisions are predictable, if not rational in the conventional theoretical sense. Such decisions are apparently intuitive even for those who vaccinate. Across individuals from different backgrounds, fuzzy impressionistic thinking and simple values seem to matter more than reflective reasoning and analytical acumen. This research implies that public health efforts should eschew giving information whose gist is not understood, stamping out emotions without knowing why they arise, or imposing mandates that are resented because they seem arbitrary. Policy and practice can benefit from having insight into how people think, and therefore feel, and why that determines their choices.

## Figures and Tables

**Table 1:** Descriptive statistics for study variables.

Variable				Young Adult Sample (*N* = 720)					Community Sample 2 (*N* = 185)				
	# Items	Theoretical		Observed		α	*M*	*SD*	Observed		α	*M*	*SD*
		Min.	Max.	Min.	Max.				Min.	Max.			
Background Predictors													
Accessibility	4	1.000	5.000	1.000	5.000	0.905	3.980	0.767	2.000	5.000	0.908	4.636	0.705
Knowledge Rating	51	1.000	5.000	2.410	4.630	0.887	3.665	0.398	2.220	4.840	0.935	4.190	0.513
Knowledge Correct	51	−1.000	1.000	−0.330	0.920	0.845	0.434	0.236	−0.390	0.960	0.919	0.667	0.275
Key Theoretical Predictors													
Status Quo	3	1.000	5.000	1.000	5.000	0.851	2.370	0.830	1.000	5.000	0.819	1.827	0.985
Gist Principles	11	1.000	5.000	1.000	5.000	0.909	3.569	0.644	1.000	5.000	0.961	4.007	1.099
Global Benefits Flu Vax	1	1.000	4.000	1.000	4.000	n/a	3.182	0.813	1.000	4.000	n/a	3.170	1.033
Global Risks Flu Vax	1	1.000	4.000	1.000	4.000	n/a	2.099	0.604	1.000	4.000	n/a	1.820	0.838
Quantitative Risk Flu Vax	11	0.000	100.000	0.000	75.180	0.907	16.298	13.261	0.450	100.000	0.900	17.631	17.079
System 2 Predictors													
SNS	8	1.000	6.000	n/a	n/a	n/a	n/a	n/a	1.000	6.000	0.858	4.686	0.836
CRT	3	0.000	3.000	n/a	n/a	n/a	n/a	n/a	0.000	3.000	0.747	1.843	1.186
Social Norms Predictors													
Friend Descriptive Norm	1	0.000	1.000	0.000	1.000	n/a	0.440	0.497	n/a	n/a	n/a	n/a	n/a
Adult Descriptive Norm	1	0.000	1.000	0.000	1.000	n/a	0.490	0.500	n/a	n/a	n/a	n/a	n/a
Friend Injunctive Norm	1	0.000	4.000	1.000	5.000	n/a	3.693	0.872	n/a	n/a	n/a	n/a	n/a
Adult Injunctive Norm	1	0.000	4.000	1.000	5.000	n/a	3.806	0.983	n/a	n/a	n/a	n/a	n/a
Outcomes													
Intentions	6	1.000	5.000	1.000	5.000	0.944	3.487	1.017	1.00	5.00	0.974	3.822	1.416
Behavior	1	0.000	1.000	0.000	1.000	n/a	0.549	0.498	0.00	1.00	n/a	0.584	0.494

Note: Min. = minimum; Max. = maximum; Vax = vaccination; n/a = not applicable; SNS = Subjective Numeracy Scale; CRT = Cognitive Reflection Test. SNS *N* = 173 and CRT *N* = 166.

**Table 2:** Hierarchical linear regressions for intentions for the young adult sample.

	*B*	SE *B*	*β*	*t*	*p*	R^2^	ΔR^2^
Model 1						0.144	0.144 ***
Constant	−0.562	0.588		−0.954	0.340		
Demographics							
Age	0.035	0.025	0.048	1.386	0.166		
Sex ^a^	0.113	0.078	0.051	1.444	0.149		
Race ^b^	0.171	0.074	0.083 *	2.329	0.020		
Ethnicity ^c^	0.011	0.114	0.004	0.100	0.920		
Background							
Accessibility	0.079	0.056	0.060	1.427	0.154		
Knowledge Rating	0.781	0.107	0.306 ***	7.272	<0.001		
Model 2						0.581	0.437 ***
Constant	1.321	0.539		2.452	0.014		
Demographics							
Age	0.032	0.018	0.044	1.781	0.075		
Sex ^a^	0.078	0.055	0.035	1.408	0.160		
Race ^b^	0.132	0.052	0.064 *	2.540	0.011		
Ethnicity ^c^	0.014	0.080	0.004	0.173	0.862		
Background							
Accessibility	−0.047	0.039	−0.035	−1.193	0.233		
Knowledge Rating	−0.203	0.091	−0.079 *	−2.237	0.026		
Key Theoretical Predictors							
Status Quo	−0.337	0.039	−0.275 ***	−8.589	<0.001		
Gist Principles	0.679	0.052	0.430 ***	12.993	<0.001		
Global Benefit	0.313	0.038	0.250 ***	8.274	<0.001		
Global Risk	−0.176	0.045	−0.105 ***	−3.902	<0.001		
Quant Risk	0.005	0.002	0.071 *	2.503	0.013		
Model 3						0.622	0.041 ***
Constant	0.806	0.516		1.563	0.119		
Demographics							
Age	0.045	0.017	0.062 **	2.648	0.008		
Sex ^a^	0.091	0.053	0.041	1.729	0.084		
Race ^b^	0.107	0.050	0.052 *	2.158	0.031		
Ethnicity ^c^	−0.038	0.077	−0.012	−0.496	0.620		
Background							
Accessibility	−0.073	0.038	−0.055	−1.937	0.053		
Knowledge Rating	−0.201	0.086	−0.079 *	−2.339	0.020		
Key Theoretical Predictors							
Status Quo	−0.311	0.037	−0.254 ***	−8.305	<0.001		
Gist Principles	0.541	0.052	0.343 ***	10.381	<0.001		
Global Benefit	0.245	0.037	0.196 ***	6.667	<0.001		
Global Risk	−0.179	0.043	−0.107 ***	−4.173	<0.001		
Quant Risk	0.006	0.002	0.085 **	3.148	0.002		
Social Norm	0.262	0.030	0.254 ***	8.735	<0.001		

Note: adult injunctive norms were included as the social norm predictor. Results for the key theoretical predictors were virtually identical when other social norms predictors were included in Model 3; see Supplemental Materials. Effects for knowledge in Models 2 and 3 disappear when demographic variables are removed, and the remaining effects are the same; see Supplemental Materials. Quant = quantitative. ^a^ Male = 0, female = 1. ^b^ Non-White = 0, White = 1. ^c^ Non-Hispanic = 0, Hispanic = 1. Bold text indicates significant results. * *p* < 0.05. ** *p* < 0.01. *** *p* < 0.001.

**Table 3:** Hierarchical logistic regressions for behavior for the young adult sample.

	*B*	SE *B*	Wald	*OR*	*p*	R^2^	ΔR^2^
Model 1						0.053	0.053 ***
Constant	−3.441	1.293	7.078	0.032	0.008		
Demographics							
Age	0.022	0.056	0.157	1.022	0.692		
Sex ^a^	−0.081	0.170	0.228	0.922	0.633		
Race ^b^	0.267	0.159	2.836	1.307	0.092		
Ethnicity ^c^	−0.363	0.247	2.157	0.695	0.142		
Background							
Accessibility	0.072	0.120	0.356	1.074	0.551		
Knowledge Rating	0.781	0.236	11.009	2.185 ***	<0.001		
Model 2						0.319	0.266 ***
Constant	1.461	1.896	0.593	4.309	0.441		
Demographics							
Age	0.005	0.062	0.006	1.005	0.940		
Sex ^a^	−0.142	0.193	0.538	0.868	0.463		
Race ^b^	0.236	0.180	1.718	1.266	0.190		
Ethnicity ^c^	−0.481	0.274	3.084	0.618	0.079		
Background							
Accessibility	−0.134	0.138	0.940	0.875	0.332		
Knowledge Rating	−0.824	0.321	6.588	0.439 *	0.010		
Key Theoretical Predictors							
Status Quo	−0.877	0.147	35.733	0.416 ***	<0.001		
Gist Principles	1.053	0.193	29.669	2.867 ***	<0.001		
Global Benefit	0.398	0.128	9.646	1.489 **	0.002		
Global Risk	−0.475	0.161	8.726	0.622 **	0.003		
Quant Risk	0.019	0.008	5.923	1.019 *	0.015		
Model 3						0.366	0.047 ***
Constant	0.738	1.949	0.143	2.092	0.705		
Demographics							
Age	0.040	0.063	0.391	1.040	0.532		
Sex ^a^	−0.140	0.197	0.503	0.869	0.478		
Race ^b^	0.159	0.185	0.741	1.173	0.389		
Ethnicity ^c^	−0.497	0.282	3.099	0.608	0.078		
Background							
Accessibility	−0.176	0.142	1.531	0.839	0.216		
Knowledge Rating	−0.805	0.330	5.941	0.447 *	0.015		
Key Theoretical Predictors							
Status Quo	−0.819	0.150	29.806	0.441 ***	<0.001		
Gist Principles	0.991	0.197	25.324	2.693 ***	<0.001		
Global Benefit	0.382	0.133	8.262	1.465 **	0.004		
Global Risk	−0.522	0.167	9.810	0.593 **	0.002		
Quant Risk	0.017	0.008	4.748	1.017 *	0.029		
Social Norm	1.056	0.184	32.901	2.876 ***	<0.001		

Note: Nagelkerke R^2^ is reported. Asterisks for ΔR^2^ represent significance for the likelihood ratio test comparing the models. Friend descriptive norms were included as the social norm predictor. Results for the key theoretical predictors were virtually identical when other social norms predictors were included in Model 3; see Supplemental Materials. *OR* = odds ratio; Quant = quantitative. ^a^ Male = 0, female = 1. ^b^ Non-White = 0, White = 1. ^c^ Non-Hispanic = 0, Hispanic = 1. Bold text indicates significant results. * *p* < 0.05. ** *p* < 0.01. *** *p* < 0.001.

**Table 4:** Hierarchical linear regressions for intentions for the community sample.

	*B*	SE *B*	*β*	*t*	*p*	R^2^	ΔR^2^
Model 1						0.574	0.574 ***
Constant	−5.019	0.717		−6.997	<0.001		
Demographics							
Age	0.007	0.006	0.066	1.219	0.225		
Sex ^a^	0.020	0.167	0.006	0.117	0.907		
Race ^b^	−0.005	0.199	−0.001	−0.025	0.980		
Ethnicity ^c^	0.101	0.253	0.021	0.398	0.691		
Background							
Accessibility	−0.012	0.109	−0.006	−0.111	0.912		
Knowledge Rating	2.049	0.153	0.745 ***	13.419	<0.001		
Model 2						0.800	0.226 ***
Constant	−0.232	0.952		−0.243	0.808		
Demographics							
Age	0.005	0.004	0.049	1.289	0.199		
Sex ^a^	0.067	0.119	0.021	0.565	0.573		
Race ^b^	0.071	0.153	0.019	0.463	0.644		
Ethnicity ^c^	−0.153	0.179	−0.032	−0.855	0.394		
Background							
Accessibility	0.123	0.078	0.063	1.588	0.114		
Knowledge Rating	0.116	0.221	0.042	0.526	0.600		
Key Theoretical Predictors							
Status Quo	−0.273	0.090	−0.191 **	−3.039	0.003		
Gist Principles	0.474	0.111	0.363 ***	4.258	<0.001		
Global Benefit	0.472	0.089	0.343 ***	5.324	<0.001		
Global Risk	−0.170	0.087	−0.101	−1.952	0.053		
Quant Risk	0.006	0.004	0.071	1.316	0.190		
Model 3						0.800	0.000
Constant	−0.383	0.986		−0.389	0.698		
Demographics							
Age	0.005	0.004	0.047	1.239	0.217		
Sex ^a^	0.086	0.123	0.027	0.697	0.487		
Race ^b^	0.064	0.154	0.017	0.413	0.680		
Ethnicity ^c^	−0.159	0.180	−0.033	−0.881	0.380		
Background							
Accessibility	0.119	0.078	0.060	1.516	0.132		
Knowledge Rating	0.098	0.223	0.036	0.439	0.661		
Key Theoretical Predictors							
Status Quo	−0.274	0.090	−0.191 **	−3.039	0.003		
Gist Principles	0.478	0.112	0.366 ***	4.277	<0.001		
Global Benefit	0.480	0.090	0.349 ***	5.343	<0.001		
Global Risk	−0.166	0.087	−0.099	−1.906	0.059		
Quant Risk	0.006	0.004	0.070	1.294	0.198		
SNS	0.043	0.072	0.024	0.605	0.546		

Note: when demographics are excluded, global risk is a significant predictor in Model 2 (*β* = −0.106, SE = 0.078, *p* = 0.025) and Model 3 (*β* = −0.106, SE = 0.078, *p* = 0.026). Quant = quantitative; SNS = Subjective Numeracy Scale. ^a^ Male = 0, female = 1. ^b^ Non-White = 0, White = 1. ^c^ Non-Hispanic = 0, Hispanic = 1. Bold text indicates significant results. ** *p* < 0.01. *** *p* < 0.001.

**Table 5:** Hierarchical logistic regressions for behavior for the community sample.

	*B*	SE *B*	Wald	*OR*	*p*	R^2^	ΔR^2^
Model 1						0.417	0.417 ***
Constant	−15.644	2.820	30.772	0.000	<0.001		
Demographics							
Age	0.026	0.015	2.819	1.026	0.093		
Sex ^a^	−0.004	0.439	0.000	0.996	0.993		
Race ^b^	−0.025	0.513	0.002	0.976	0.962		
Ethnicity ^c^	0.593	0.698	0.724	1.810	0.395		
Background							
Accessibility	0.651	0.284	5.271	1.918 *	0.022		
Knowledge Rating	2.811	0.598	22.124	16.624 ***	<0.001		
Model 2						0.582	0.165 ***
Constant	−11.129	4.251	6.853	0.000	0.009		
Demographics							
Age	0.026	0.017	2.223	1.026	0.136		
Sex ^a^	0.032	0.515	0.004	1.032	0.951		
Race ^b^	0.308	0.604	0.261	1.361	0.609		
Ethnicity ^c^	−0.070	0.819	0.007	0.933	0.932		
Background							
Accessibility	0.865	0.330	6.846	2.374 **	0.009		
Knowledge Rating	−0.358	0.952	0.142	0.699	0.707		
Key Theoretical Predictors							
Status Quo	−0.077	0.376	0.041	0.926	0.839		
Gist Principles	1.294	0.518	6.245	3.647 *	0.012		
Global Benefit	0.841	0.356	5.590	2.318 *	0.018		
Global Risk	−0.314	0.365	0.738	0.731	0.390		
Quant Risk	0.021	0.023	0.835	1.021	0.361		
Model 3						0.586	0.054
Constant	−9.984	4.356	5.252	0.000	0.022		
Demographics							
Age	0.026	0.017	2.297	1.027	0.130		
Sex ^a^	−0.115	0.531	0.047	0.891	0.828		
Race ^b^	0.399	0.606	0.434	1.491	0.510		
Ethnicity ^c^	−0.049	0.830	0.003	0.953	0.953		
Background							
Accessibility	0.920	0.340	7.334	2.508 **	0.007		
Knowledge Rating	−0.205	0.971	0.044	0.815	0.833		
Key Theoretical Predictors							
Status Quo	−0.109	0.377	0.083	0.897	0.774		
Gist Principles	1.274	0.516	6.081	3.574 *	0.014		
Global Benefit	0.760	0.360	4.461	2.139 *	0.035		
Global Risk	−0.350	0.366	0.911	0.705	0.340		
Quant Risk	0.021	0.023	0.804	1.021	0.370		
SNS	−0.334	0.351	0.907	0.716	0.341		

Note: Nagelkerke R^2^ is reported. Asterisks for ΔR^2^ represent significance for the likelihood ratio test comparing the models. OR = odds ratio; Quant = quantitative; SNS = Subjective Numeracy Scale. ^a^ Male = 0, female = 1. ^b^ Non-White = 0, White = 1. ^c^ Non-Hispanic = 0, Hispanic = 1. Bold text indicates significant results. * *p* < 0.05. ** *p* < 0.01. *** *p* < 0.001.

**Table 6:** Fuzzy-trace theory regressions for intentions and behavior for young adult and community samples.

	Young Adults												
**A**	Intentions						Behavior						
	*B*	SE *B*	*β*	*t*	*p*	R^2^		*B*	SE *B*	Wald	*OR*	*p*	R^2^
						0.571							0.299
Constant	1.090	0.249		4.369	<0.001		Constant	−2.218	0.851	6.787	0.109	0.009	
Status Quo	−0.305	0.036	−0.249 ***	−8.450	<0.001		Status Quo	−0.686	0.126	29.514	0.503 ***	<0.001	
Gist Principles	0.658	0.052	0.417 ***	12.583	<0.001		Gist Principles	0.966	0.188	26.376	2.627 ***	<0.001	
Global Benefit	0.308	0.038	0.246 ***	8.156	<0.001		Global Benefit	0.341	0.126	7.330	1.407 **	0.007	
Global Risk	−0.157	0.045	−0.093 ***	−3.499	<0.001		Global Risk	−0.405	0.155	6.803	0.667 **	0.009	
Quant Risk	0.008	0.002	0.099 ***	3.646	<0.001		Quant Risk	0.026	0.007	12.764	1.026 ***	<0.001	
	Community												
**B**	Intentions						Behavior						
	*B*	SE *B*	*β*	*t*	*p*	R^2^		*B*	SE *B*	Wald	*OR*	*p*	R^2^
						0.786							0.501
Constant	1.029	0.466		2.208	0.029		Constant	−6.661	2.233	8.896	0.001	0.003	
Status Quo	−0.292	0.079	−0.203 ***	−3.693	<0.001		Status Quo	−0.087	0.323	0.073	0.917	0.787	
Gist Principles	0.487	0.092	0.378 ***	5.319	<0.001		Gist Principles	1.103	0.394	7.844	3.012 **	0.005	
Global Benefit	0.496	0.083	0.362 ***	6.006	<0.001		Global Benefit	0.825	0.305	7.293	2.281 **	0.007	
Global Risk	−0.156	0.075	−0.092 *	−2.064	0.040		Global Risk	−0.051	0.303	0.028	0.950	0.867	
Quant Risk	0.005	0.004	0.057	1.256	0.211		Quant Risk	0.003	0.019	0.031	1.003	0.860	

*Note:* Quant = Quantitative. For behavior, Nagelkerke R^2^ is reported. Bold text indicates significant results. * *p* < 0.05. ** *p* < 0.01. *** *p* < 0.001.

**Table 7:** Summary of predictions of fuzzy-trace theory and expectancy value models with relevant results.

	Fuzzy-Trace Theory (FTT)	Expectancy Value (EV) Models
Status Quo Gist	Predicted by FTT and supported by evidence from framing problems for which expectancy-value models have been disconfirmed (see Reyna et al., 2023, for critical tests of expectancy-value models); also supported by other FTT research on health decision-making (e.g., on antibiotic decisions, Marti et al., 2022; see Reyna et al., 2022). Results here confirmed FTT’s predictions.	Status-quo gist does not exist in EV models and would not have any predicted relation to vaccination behaviors. Results here disconfirmed EV models.
Gist Principles	The operationalization of gist principles as simple gist representations of core values, and their relation to vaccination intentions and behaviors, are predicted by FTT and supported by prior FTT research on decision-making about concussions, HIV-prevention, COVID-19, and other health decision-making, including causal designs (e.g., Edelson et al., 2024; Garavito et al., 2021; Mills et al., 2008; Reyna et al., 2011; Reyna & Mills, 2014). Gist principles predicted intentions and behaviors significantly, controlling for social norms (which were also significant), indicating that gist principles capture variance not explained by social norms.	Gist principles—simple core values mentally represented as gist—do not exist in EV models. The closest but not matching concept is social norms. Social norms are depicted as more specific than gist principles in virtually all EV models and incorporate social conformity. Here we distinguished gist principles from social norms. Social norms predicted intentions and behaviors, as predicted by EV models. Gist principles predicted intentions and behaviors, too, significantly beyond effects of social norms, which is not consistent with EV models.
Global Benefits from Flu Vax	The operationalization of benefits as global gist representations with categorical responses, and their relation to vaccination intentions and behaviors, are predicted by FTT and supported by prior FTT research on decision-making (e.g., Mills et al., 2008; Reyna et al., 2011; for a review, see Blalock & Reyna, 2016). Results here confirmed FTT’s overall predictions that global benefits would predict vaccination intentions and behaviors, and the greater impact of global benefits over global risks (e.g., Reyna & Farley, 2006).	Global gist representations of benefits do not exist in EV models, contrary to extensive evidence about mental representations. Benefits in EV models are conceptualized in terms of specific outcomes (consequences) and their probabilities, such as efficacy (e.g., perceived probability of preventing hospitalization, death, missing work due to the flu, and in some models, probability of preventing these outcomes for others). EV models would not predict that global, non-specific measures would adequately capture and predict perceptions of the benefits of a flu vaccine, disconfirmed by robust results here for the global benefits item. Moreover, specific benefits from the flu vaccine are unlikely to be sufficiently large or evaluable to encourage vaccine uptake by individuals: “Vaccines are administered as prophylactics to healthy individuals and the risks of vaccines (real or alleged) are visible while their benefits are impossible to evaluate from an individual perspective.” (Dubé et al., 2013, p. 1769).
Global Risks from Flu Vax	The operationalization of risks as global gist representations with categorical responses, and their relation to vaccination intentions and behaviors, are predicted by FTT and supported by prior FTT research on decision-making (see references above). Results here confirmed FTT’s overall predictions that global risks would predict vaccination intentions and behaviors, and the greater impact of global risks over specific quantities of risks of specific outcomes (i.e., Quantitative Risks).	Global gist representations of risks do not exist in EV models, contrary to extensive evidence about mental representations. Risks in EV models are conceptualized in terms of specific outcomes (consequences) and their probabilities for the individual, such as safety (e.g., perceived probability of hospitalization, death, allergic reaction, or other consequences from the flu vaccine), and in some models, the probability of these outcomes for other people. EV models would not predict that global, non-specific measures would adequately capture and predict perceptions of the risks of a flu vaccine, disconfirmed by results here for the global risks item.
Quantitative Risks from Flu Vax	The current study compares the predictive value of different types of measures: global risk of the flu vaccine (overall gist, above) to quantitative risk of the flu vaccine (specific outcomes assessed on a 0–100% scale). Results here confirmed FTT’s prediction that global measures of risk out-predict specific quantitative measures of risk. Quantitative risk remained nonsignificant when controlling for numeracy.	EV models would predict that perceived risk of specific outcomes (judged as precisely as possible) would be more likely to predict vaccination intentions and behaviors than vague global risk categorizations (e.g., Fishbein, 2008). Modern dual-process theories have predicted that those higher in System 2 processes (CRT and numeracy) should be less subject to misinformation about vaccination and other health behaviors and otherwise be better able to trade off risks and benefits (e.g., E. Peters, 2020; Scherer & Pennycook, 2020). Results here did not support these predictions.

Note: Vax = vaccination. Expectancy value theories, as discussed in the introduction, include the health belief model, theory of reasoned action, theory of planned behavior, protection–motivation theory, and other variants. For all theories, we are not counting post hoc speculation as theoretically motivated prediction. Analyses controlled for background variables such as demographics, accessibility (self-efficacy, perceived behavioral control, and affordability), and knowledge (assessed objectively), with FTT predictors accounting for unique variance beyond these background variables. These results, too, are consistent with FTT’s predictions, for example, that knowledge of specific facts about flu and vaccination is not sufficient by itself to predict intentions and behaviors; mental representations of gist add predictive value. EV models do not make the latter prediction.

## Data Availability

The data presented in this study are available upon request from the corresponding author.

## Note

1 Additional items were included in both surveys, to be published elsewhere.

## Supplementary Materials

Supporting information and analyses can be downloaded at https://www.mdpi.com/article/10.3390/bs15121645/s1.
