# Supplementary material for: A New Look at Vaccination Behaviors and Intentions: The Case of Influenza
Source: Behav Sci (Basel). 2025 Nov 30;15(12):1645. doi: 10.3390/bs15121645 (PMC12729583; doi:10.3390/bs15121645)
Supplement: Supplementary file 1 [file behavsci-15-01645-s001.zip › behavsci-3920179-supplementary.pdf]

Supplemental Materials
A New Look at Vaccination Behaviors and Intentions: The Case of Influenza

Table of Contents

YOUNG ADULT SAMPLE 3

BIVARIATE CORRELATIONS 3
LINEAR REGRESSIONS FOR INTENTIONS WITH DEMOGRAPHICS – INCLUDING QUANTITATIVE AND SOCIAL NORMS 5
Friend Descriptive *5*
Adult Descriptive *6*
Friends Injunctive *7*
Adult Injunctive *9*
Average Descriptive Norms (Friend and Adult) *10*
Average Injunctive Norms (Friend and Adult) *12*
LOGISTIC REGRESSION FOR BEHAVIOR WITH DEMOGRAPHICS – INCLUDING QUANTITATIVE AND SOCIAL NORMS 13
Friend Descriptive *13*
Adult Descriptive *16*
Friend Injunctive *17*
Adult Injunctive *18*
Average Descriptive Norms (Friend and Adult) *19*
Average Injunctive Norms (Friend and Adult) *20*
LINEAR REGRESSION FOR INTENTIONS WITHOUT DEMOGRAPHICS 21
Friend Descriptive *21*
Adult Descriptive *22*
Friend Injunctive *23*
Adult Injunctive *24*
LOGISTIC REGRESSION FOR BEHAVIOR WITHOUT DEMOGRAPHICS 25
Friend Descriptive *25*
Adult Descriptive *27*
Friend Injunctive *28*
Adult Injunctive *29*
LINEAR REGRESSION FOR INTENTIONS (ONLY FTT PREDICTORS) 30
LOGISTIC REGRESSION FOR BEHAVIOR (ONLY FTT PREDICTORS) 31
LINEAR REGRESSION FOR INTENTIONS WITHOUT QUANTITATIVE RISK (FRIEND DESCRIPTIVE NORMS) 32
LINEAR REGRESSION FOR INTENTIONS WITHOUT QUANTITATIVE RISK (ADULT DESCRIPTIVE NORMS) 34
LINEAR REGRESSION FOR INTENTIONS WITHOUT QUANTITATIVE RISK (FRIEND INJUNCTIVE NORMS) 35
LINEAR REGRESSION FOR INTENTIONS WITHOUT QUANTITATIVE RISK (ADULT INJUNCTIVE NORMS) 37
LOGISTIC REGRESSION FOR BEHAVIOR WITHOUT QUANTITATIVE RISK (FRIEND DESCRIPTIVE NORMS) 39

Logistic Regression for Behavior Without Quantitative Risk (Adult Descriptive Norms) 42
Logistic Regression for Behavior Without Quantitative Risk (Friend Injunctive Norms) 44
Logistic Regression for Behavior Without Quantitative Risk (Adult Injunctive Norms) 46
Linear Regression for Intentions Without Knowledge (Friend Descriptive Norms) 49
Linear Regression for Intentions Without Knowledge (Adult Descriptive Norms) 51
Linear Regression for Intentions Without Knowledge (Friend Injunctive Norms) 52
Linear Regression for Intentions Without Knowledge (Adult Injunctive Norms) 54
Logistic Regression for Behavior Without Knowledge (Friend Descriptive Norms) 56
Logistic Regression for Behavior Without Knowledge (Adult Descriptive Norms) 58
Logistic Regression for Behavior Without Knowledge (Friend Injunctive Norms) 61
Logistic Regression for Behavior Without Knowledge (Adult Injunctive Norms) 63

COMMUNITY SAMPLE 66

Bivariate Correlations 66
Linear Regression for Intentions with Demographics – Including Quantitative and SNS 68
Logistic Regression for Behavior with Demographics – Including Quantitative and SNS 69
Linear Regression for Intentions WITHOUT Demographics 71
Logistic Regression for Behavior WITHOUT Demographics 73
Linear Regression for Intentions (Only FTT Predictors) 75
Logistic Regression for Behavior (Only FTT Predictors) 76
Linear Regression for Intentions Without Quantitative Risk 77
Logistic Regression for Behavior without Quantitative Risk 79
Linear Regression for Intentions Without Knowledge 81
Logistic Regression for Behavior Without Knowledge 83

MATERIALS 86

Young Adult Sample

Bivariate Correlations

	Age	Sex	White	Black	Asian	Ethnicity	Access	Knowledge	KnowledgeCorrect
Age	1.000	-0.026	**-0.095***	-0.022	**0.129****	-0.010	-0.003	0.027	0.028
Sex[a]	-0.026	1.000	0.064	-0.022	-0.020	-0.056	**0.148****	**0.122****	**0.142****
White[b]	**-0.095***	0.064	1.000	**-0.346****	**-0.774****	0.040	**0.147****	**0.176****	**0.161****
Black[c]	-0.022	-0.022	**-0.346****	1.000	**-0.181****	-0.048	**-0.087***	**-0.103****	**-0.101****
Asian[d]	**0.129****	-0.020	**-0.774****	**-0.181****	1.000	**-0.172****	**-0.094***	**-0.106****	**-0.085***
Ethnicity[e]	-0.010	-0.056	0.040	-0.048	**-0.172****	1.000	**-0.103****	**-0.105****	**-0.100****
Access	-0.003	**0.148****	**0.147****	**-0.087***	**-0.094***	**-0.103****	1.000	**0.555****	**0.520****
Knowledge	0.027	**0.122****	**0.176****	**-0.103****	**-0.106****	**-0.105****	**0.555****	1.000	**0.946****
KnowledgeCorrect	0.028	**0.142****	**0.161****	**-0.101****	**-0.085***	**-0.100****	**0.520****	**0.946****	1
StatusQuo	-0.026	**-0.080***	**-0.167****	**0.101****	**0.112****	0.049	**-0.390****	**-0.556****	**-0.503****
GistPrinciples	0.019	0.068	0.070	**-0.147****	0.021	-0.064	**0.319****	**0.459****	**0.413****
GlobalBenefits	-0.015	**0.135****	**0.079***	**-0.096***	-0.018	-0.051	**0.270****	**0.362****	**0.358****
GlobalRisks	0.004	0.062	-0.061	**0.154****	-0.034	0.009	**-0.194****	**-0.320****	**-0.317****
QuantRisk	0.013	-0.008	-0.009	**0.098****	-0.064	0.046	**-0.256****	**-0.430****	**-0.471****
FriendDescr	**-0.078***	0.047	**0.126****	**-0.114****	-0.065	-0.021	**0.145****	**0.135****	**0.117****
AdultDescr	-0.066	0.051	**0.139****	**-0.100****	**-0.104****	-0.038	**0.159****	**0.205****	**0.192****
FriendInjunct	0.035	0.062	**0.112****	**-0.117****	-0.039	-0.021	**0.188****	**0.313****	**0.303****
AdultInjunct	-0.071	0.049	**0.121****	**-0.088***	-0.073	0.019	**0.286****	**0.341****	**0.319****
Intentions	0.047	**0.101****	**0.145****	**-0.150****	-0.032	-0.035	**0.249****	**0.361****	**0.321****
Behavior	0.009	0.010	**0.092***	**-0.122****	-0.011	-0.069	**0.124****	**0.181****	**0.161****

	StatusQuo	GistPrinciples	GlobalBenefits	GlobalRisks	QuantRisk	FriendDescr	AdultDescr	FriendInjunct	AdultInjunct	Intentions	Behavior
Age	-0.026	0.019	-0.015	0.004	0.013	**-0.078***	-0.066	0.035	-0.071	0.047	0.009
Sex[a]	**-0.080***	0.068	**0.135****	0.062	-0.008	0.047	0.051	0.062	0.049	**0.101****	0.010
White[b]	**-0.167****	0.070	**0.079***	-0.061	-0.009	**0.126****	**0.139****	**0.112****	**0.121****	**0.145****	**0.092***
Black[c]	**0.101****	**-0.147****	**-0.096***	**0.154****	**0.098****	**-0.114****	**-0.100****	**-0.117****	**-0.088***	**-0.150****	**-0.122****
Asian[d]	**0.112****	0.021	-0.018	-0.034	-0.064	-0.065	**-0.104****	-0.039	-0.073	-0.032	-0.011
Ethnicity[e]	0.049	-0.064	-0.051	0.009	0.046	-0.021	-0.038	-0.021	0.019	-0.035	-0.069
Access	**-0.390****	**0.319****	**0.270****	**-0.194****	**-0.256****	**0.145****	**0.159****	**0.188****	**0.286****	**0.249****	**0.124****
Knowledge	**-0.556****	**0.459****	**0.362****	**-0.320****	**-0.430****	**0.135****	**0.205****	**0.313****	**0.341****	**0.361****	**0.181****
KnowledgeCorrect	**-0.503****	**0.413****	**0.358****	**-0.317****	**-0.471****	**0.117****	**0.192****	**0.303****	**0.319****	**0.321****	**0.161****

	StatusQuo	GistPrinciples	GlobalBenefits	GlobalRisks	QuantRisk	FriendDescr	AdultDescr	FriendInjunct	AdultInjunct	Intentions	Behavior
StatusQuo	1.000	-0.532**	-0.399**	0.217**	0.230**	-0.242**	-0.320**	-0.336**	-0.394**	-0.567**	-0.382**
GistPrinciples	-0.532**	1.000	0.568**	-0.225**	-0.294**	0.235**	0.331**	0.443**	0.545**	0.681**	0.399**
GlobalBenefits	-0.399**	0.568**	1.000	-0.113**	-0.223**	0.180**	0.263**	0.404**	0.472**	0.571**	0.323**
GlobalRisks	0.217**	-0.225**	-0.113**	1.000	0.365**	-0.054	-0.107**	-0.147**	-0.152**	-0.233**	-0.163**
QuantRisk	0.230**	-0.294**	-0.223**	0.365**	1.000	-0.040	-0.047	-0.213**	-0.239**	-0.169**	-0.051
FriendDescr	-0.242**	0.235**	0.180**	-0.054	-0.040	1.000	0.425**	0.348**	0.259**	0.271**	0.314**
AdultDescr	-0.320**	0.331**	0.263**	-0.107**	-0.047	0.425**	1.000	0.254**	0.429**	0.427**	0.358**
FriendInjunct	-0.336**	0.443**	0.404**	-0.147**	-0.213**	0.348**	0.254**	1.000	0.571**	0.439**	0.286**
AdultInjunct	-0.394**	0.545**	0.472**	-0.152**	-0.239**	0.259**	0.429**	0.571**	1.000	0.590**	0.369**
Intentions	-0.567**	0.681**	0.571**	-0.233**	-0.169**	0.271**	0.427**	0.439**	0.590**	1.000	0.569**
Behavior	-0.382**	0.399**	0.323**	-0.163**	-0.051	0.314**	0.358**	0.286**	0.369**	0.569**	1.000

**p < .01, *p < .05

[a] 0 = male, 1 = female

[b] 0 = non-White, 1 = While

[c] 0 = non-Black, 1 = Black

[d] 0 = non-Asian, 1 = Asian

[e] 0 = not Hispanic, 1 = Hispanic

Note. N = 722 for each bivariate correlation pair except for correlations with GlobalBenefits where N = 720.

Linear Regressions for Intentions with Demographics – Including Quantitative and Social Norms

Friend Descriptive

Model Summary

					Change Statistics				
Model	R	R Square	Adjusted R Square	Std. Error of the Estimate	R Square Change	F Change	df1	df2	Sig. F Change
1	0.380	0.144	0.137	0.94441	0.144	20.046	6	713	<0.001
2	0.762	0.581	0.575	0.66299	0.437	147.758	5	708	<0.001
3	0.766	0.586	0.579	0.65970	0.005	8.066	1	707	0.005

Coefficients[a]

		Unstandardized Coefficients		Standardized Coefficients	t	Sig.	Collinearity Statistics	
Model		B	Std. Error	Beta			Tolerance	VIF
1	(Constant)	-0.562	0.588		-0.954	0.340		
	Age	0.035	0.025	0.048	1.386	0.166	0.989	1.011
	Sex	0.113	0.078	0.051	1.444	0.149	0.971	1.029
	White	0.171	0.074	**0.083**	2.329	0.020	0.952	1.050
	Hispanic	0.011	0.114	0.004	0.100	0.920	0.980	1.020
	Access	0.079	0.056	0.060	1.427	0.154	0.683	1.465
	Knowledge	0.781	0.107	**0.306**	7.272	<0.001	0.679	1.473
2	(Constant)	1.321	0.539		2.452	0.014		
	Age	0.032	0.018	0.044	1.781	0.075	0.986	1.014
	Sex	0.078	0.055	0.035	1.408	0.160	0.951	1.052
	White	0.132	0.052	**0.064**	2.540	0.011	0.938	1.066
	Hispanic	0.014	0.080	0.004	0.173	0.862	0.979	1.021
	Access	-0.047	0.039	-0.035	-1.193	0.233	0.671	1.491
	Knowledge	-0.203	0.091	**-0.079**	-2.237	0.026	0.470	2.126
	StatusQuo	-0.337	0.039	**-0.275**	-8.589	<0.001	0.576	1.735
	GistPrinciples	0.679	0.052	**0.430**	12.993	<0.001	0.540	1.853
	GlobalBenefits	0.313	0.038	**0.250**	8.274	<0.001	0.648	1.544
	GlobalRisks	-0.176	0.045	**-0.105**	-3.902	<0.001	0.820	1.219
	QuantRisk	0.005	0.002	**0.071**	2.503	0.013	0.741	1.350

Coefficients[a]

Model		Unstandardized Coefficients B	Unstandardized Coefficients Std. Error	Standardized Coefficients Beta	t	Sig.	Collinearity Statistics Tolerance	Collinearity Statistics VIF
3	(Constant)	1.203	0.538		2.238	0.026		
	Age	0.036	0.018	**0.049**	2.020	0.044	0.979	1.021
	Sex	0.076	0.055	0.034	1.386	0.166	0.951	1.052
	White	0.120	0.052	**0.058**	2.304	0.021	0.931	1.074
	Hispanic	0.016	0.080	0.005	0.198	0.843	0.979	1.021
	Access	-0.052	0.039	-0.039	-1.333	0.183	0.669	1.494
	Knowledge	-0.191	0.090	**-0.075**	-2.120	0.034	0.469	2.130
	StatusQuo	-0.323	0.039	**-0.264**	-8.210	<0.001	0.567	1.762
	GistPrinciples	0.662	0.052	**0.420**	12.664	<0.001	0.533	1.876
	GlobalBenefits	0.309	0.038	**0.247**	8.206	<0.001	0.647	1.546
	GlobalRisks	-0.176	0.045	**-0.105**	-3.919	<0.001	0.820	1.219
	QuantRisk	0.005	0.002	**0.068**	2.403	0.017	0.740	1.352
	FriendDescr	0.148	0.052	**0.072**	2.840	0.005	0.904	1.106

a. Dependent Variable: Intentions

Adult Descriptive

Model Summary

Model	R	R Square	Adjusted R Square	Std. Error of the Estimate	Change Statistics: R Square Change	F Change	df1	df2	Sig. F Change
1	0.380	0.144	0.137	0.94441	0.144	20.046	6	713	<0.001
2	0.762	0.581	0.575	0.66299	0.437	147.758	5	708	<0.001
3	0.777	0.604	0.597	0.64520	0.023	40.579	1	707	<0.001

Coefficients[a]

Model		Unstandardized Coefficients B	Unstandardized Coefficients Std. Error	Standardized Coefficients Beta	t	Sig.	Collinearity Statistics Tolerance	Collinearity Statistics VIF
1	(Constant)	-0.562	0.588		-0.954	0.340		
	Age	0.035	0.025	0.048	1.386	0.166	0.989	1.011
	Sex	0.113	0.078	0.051	1.444	0.149	0.971	1.029
	White	0.171	0.074	**0.083**	2.329	0.020	0.952	1.050

Coefficients[a]

Model		Unstandardized Coefficients B	Std. Error	Standardized Coefficients Beta	t	Sig.	Collinearity Statistics Tolerance	VIF
	Hispanic	0.011	0.114	0.004	0.100	0.920	0.980	1.020
	Access	0.079	0.056	0.060	1.427	0.154	0.683	1.465
	Knowledge	0.781	0.107	**0.306**	7.272	<0.001	0.679	1.473
2	(Constant)	1.321	0.539		2.452	0.014		
	Age	0.032	0.018	0.044	1.781	0.075	0.986	1.014
	Sex	0.078	0.055	0.035	1.408	0.160	0.951	1.052
	White	0.132	0.052	**0.064**	2.540	0.011	0.938	1.066
	Hispanic	0.014	0.080	0.004	0.173	0.862	0.979	1.021
	Access	-0.047	0.039	-0.035	-1.193	0.233	0.671	1.491
	Knowledge	-0.203	0.091	**-0.079**	-2.237	0.026	0.470	2.126
	StatusQuo	-0.337	0.039	**-0.275**	-8.589	<0.001	0.576	1.735
	GistPrinciples	0.679	0.052	**0.430**	12.993	<0.001	0.540	1.853
	GlobalBenefits	0.313	0.038	**0.250**	8.274	<0.001	0.648	1.544
	GlobalRisks	-0.176	0.045	**-0.105**	-3.902	<0.001	0.820	1.219
	QuantRisk	0.005	0.002	**0.071**	2.503	0.013	0.741	1.350
3	(Constant)	1.145	0.525		2.181	0.030		
	Age	0.040	0.017	**0.055**	2.310	0.021	0.980	1.020
	Sex	0.076	0.054	0.034	1.403	0.161	0.951	1.052
	White	0.104	0.051	**0.050**	2.045	0.041	0.931	1.074
	Hispanic	0.025	0.078	0.008	0.314	0.753	0.979	1.022
	Access	-0.049	0.038	-0.037	-1.269	0.205	0.671	1.491
	Knowledge	-0.197	0.088	**-0.077**	-2.237	0.026	0.470	2.126
	StatusQuo	-0.300	0.039	**-0.245**	-7.779	<0.001	0.564	1.774
	GistPrinciples	0.627	0.051	**0.397**	12.171	<0.001	0.526	1.901
	GlobalBenefits	0.295	0.037	**0.236**	8.002	<0.001	0.644	1.553
	GlobalRisks	-0.165	0.044	**-0.098**	-3.740	<0.001	0.819	1.222
	QuantRisk	0.004	0.002	**0.056**	2.017	0.044	0.736	1.360
	AdultDescr	0.335	0.053	**0.165**	6.370	<0.001	0.837	1.195

a. Dependent Variable: Intentions

Friends Injunctive

Model Summary

Model	R	R Square	Adjusted R Square	Std. Error of the Estimate	Change Statistics				
					R Square Change	F Change	df1	df2	Sig. F Change
1	0.380	0.144	0.137	0.94441	0.144	20.046	6	713	<0.001
2	0.762	0.581	0.575	0.66299	0.437	147.758	5	708	<0.001
3	0.767	0.589	0.582	0.65738	0.008	13.122	1	707	<0.001

Coefficients[a]

Model		Unstandardized Coefficients		Standardized Coefficients	t	Sig.	Collinearity Statistics	
		B	Std. Error	Beta			Tolerance	VIF
1	(Constant)	-0.562	0.588		-0.954	0.340		
	Age	0.035	0.025	0.048	1.386	0.166	0.989	1.011
	Sex	0.113	0.078	0.051	1.444	0.149	0.971	1.029
	White	0.171	0.074	**0.083**	2.329	0.020	0.952	1.050
	Hispanic	0.011	0.114	0.004	0.100	0.920	0.980	1.020
	Access	0.079	0.056	0.060	1.427	0.154	0.683	1.465
	Knowledge	0.781	0.107	**0.306**	7.272	<0.001	0.679	1.473
2	(Constant)	1.321	0.539		2.452	0.014		
	Age	0.032	0.018	0.044	1.781	0.075	0.986	1.014
	Sex	0.078	0.055	0.035	1.408	0.160	0.951	1.052
	White	0.132	0.052	**0.064**	2.540	0.011	0.938	1.066
	Hispanic	0.014	0.080	0.004	0.173	0.862	0.979	1.021
	Access	-0.047	0.039	-0.035	-1.193	0.233	0.671	1.491
	Knowledge	-0.203	0.091	**-0.079**	-2.237	0.026	0.470	2.126
	StatusQuo	-0.337	0.039	**-0.275**	-8.589	<0.001	0.576	1.735
	GistPrinciples	0.679	0.052	**0.430**	12.993	<0.001	0.540	1.853
	GlobalBenefits	0.313	0.038	**0.250**	8.274	<0.001	0.648	1.544
	GlobalRisks	-0.176	0.045	**-0.105**	-3.902	<0.001	0.820	1.219
	QuantRisk	0.005	0.002	**0.071**	2.503	0.013	0.741	1.350
3	(Constant)	1.176	0.536		2.195	0.028		
	Age	0.029	0.018	0.040	1.650	0.099	0.984	1.016
	Sex	0.076	0.055	0.034	1.388	0.166	0.951	1.052
	White	0.119	0.052	**0.058**	2.309	0.021	0.934	1.071
	Hispanic	0.010	0.080	0.003	0.120	0.905	0.979	1.021
	Access	-0.042	0.039	-0.032	-1.078	0.281	0.670	1.493
	Knowledge	-0.219	0.090	**-0.086**	-2.440	0.015	0.469	2.132

StatusQuo	-0.328	0.039	**-0.268**	-8.405	<0.001	0.574	1.743
GistPrinciples	0.639	0.053	**0.405**	12.085	<0.001	0.517	1.934
GlobalBenefits	0.287	0.038	**0.230**	7.529	<0.001	0.625	1.599
GlobalRisks	-0.174	0.045	**-0.103**	-3.875	<0.001	0.820	1.220
QuantRisk	0.006	0.002	**0.076**	2.711	0.007	0.739	1.353
FriendInjunct	0.118	0.032	**0.101**	3.622	<0.001	0.749	1.336

a. Dependent Variable: Intentions

Adults Injunctive

Model Summary

					Change Statistics				
Model	R	R Square	Adjusted R Square	Std. Error of the Estimate	R Square Change	F Change	df1	df2	Sig. F Change
1	0.380[a]	0.144	0.137	0.94441	0.144	20.046	6	713	<0.001
2	0.762[b]	0.581	0.575	0.66299	0.437	147.758	5	708	<0.001
3	0.789[c]	0.622	0.616	0.63032	0.041	76.295	1	707	<0.001

Coefficients[a]

		Unstandardized Coefficients		Standardized Coefficients			Collinearity Statistics	
Model		B	Std. Error	Beta	t	Sig.	Tolerance	VIF
1	(Constant)	-0.562	0.588		-0.954	0.340		
	Age	0.035	0.025	0.048	1.386	0.166	0.989	1.011
	Sex	0.113	0.078	0.051	1.444	0.149	0.971	1.029
	White	0.171	0.074	**0.083**	2.329	0.020	0.952	1.050
	Hispanic	0.011	0.114	0.004	0.100	0.920	0.980	1.020
	Access	0.079	0.056	0.060	1.427	0.154	0.683	1.465
	Knowledge	0.781	0.107	**0.306**	7.272	<0.001	0.679	1.473
2	(Constant)	1.321	0.539		2.452	0.014		
	Age	0.032	0.018	0.044	1.781	0.075	0.986	1.014
	Sex	0.078	0.055	0.035	1.408	0.160	0.951	1.052
	White	0.132	0.052	**0.064**	2.540	0.011	0.938	1.066
	Hispanic	0.014	0.080	0.004	0.173	0.862	0.979	1.021
	Access	-0.047	0.039	-0.035	-1.193	0.233	0.671	1.491
	Knowledge	-0.203	0.091	**-0.079**	-2.237	0.026	0.470	2.126
	StatusQuo	-0.337	0.039	**-0.275**	-8.589	<0.001	0.576	1.735

	GistPrinciples	0.679	0.052	**0.430**	12.993	<0.001	0.540	1.853
	GlobalBenefits	0.313	0.038	**0.250**	8.274	<0.001	0.648	1.544
	GlobalRisks	-0.176	0.045	**-0.105**	-3.902	<0.001	0.820	1.219
	QuantRisk	0.005	0.002	**0.071**	2.503	0.013	0.741	1.350
3	(Constant)	0.806	0.516		1.563	0.119		
	Age	0.045	0.017	**0.062**	2.648	0.008	0.978	1.023
	Sex	0.091	0.053	0.041	1.729	0.084	0.950	1.053
	White	0.107	0.050	**0.052**	2.158	0.031	0.935	1.070
	Hispanic	-0.038	0.077	-0.012	-0.496	0.620	0.973	1.027
	Access	-0.073	0.038	-0.055	-1.937	0.053	0.667	1.500
	Knowledge	-0.201	0.086	**-0.079**	-2.339	0.020	0.470	2.126
	StatusQuo	-0.311	0.037	**-0.254**	-8.305	<0.001	0.573	1.746
	GistPrinciples	0.541	0.052	**0.343**	10.381	<0.001	0.490	2.040
	GlobalBenefits	0.245	0.037	**0.196**	6.667	<0.001	0.619	1.616
	GlobalRisks	-0.179	0.043	**-0.107**	-4.173	<0.001	0.820	1.219
	QuantRisk	0.006	0.002	**0.085**	3.148	0.002	0.738	1.354
	AdultInjunct	0.262	0.030	**0.254**	8.735	<0.001	0.634	1.577

a. Dependent Variable: Intentions

Average Descriptive Norms (Friend and Adult)

Model Summary

					Change Statistics				
Model	R	R Square	Adjusted R Square	Std. Error of the Estimate	R Square Change	F Change	df1	df2	Sig. F Change
1	0.380[a]	0.144	0.137	0.94441	0.144	20.046	6	713	<0.001
2	0.762[b]	0.581	0.575	0.66299	0.437	147.758	5	708	<0.001
3	0.774[c]	0.599	0.592	0.64930	0.018	31.156	1	707	<0.001

a. Predictors: (Constant), Knowledge, Age, Hispanic, Sex, White, Access

b. Predictors: (Constant), Knowledge, Age, Hispanic, Sex, White, Access, GlobalRisks, GlobalBenefits, QuantRisk, StatusQuo, GistPrinciples

c. Predictors: (Constant), Knowledge, Age, Hispanic, Sex, White, Access, GlobalRisks, GlobalBenefits, QuantRisk, StatusQuo, GistPrinciples, DescriptiveNorm_2items

Coefficients[a]

Model	Unstandardized Coefficients	Standardized Coefficients	t	Sig.	Collinearity Statistics

		B	Std. Error	Beta			Tolerance	VIF
1	(Constant)	-0.562	0.588		-0.954	0.340		
	Age	0.035	0.025	0.048	1.386	0.166	0.989	1.011
	Sex	0.113	0.078	0.051	1.444	0.149	0.971	1.029
	White	0.171	0.074	**0.083**	2.329	0.020	0.952	1.050
	Hispanic	0.011	0.114	0.004	0.100	0.920	0.980	1.020
	Access	0.079	0.056	0.060	1.427	0.154	0.683	1.465
	Knowledge	0.781	0.107	**0.306**	7.272	<0.001	0.679	1.473
2	(Constant)	1.321	0.539		2.452	0.014		
	Age	0.032	0.018	0.044	1.781	0.075	0.986	1.014
	Sex	0.078	0.055	0.035	1.408	0.160	0.951	1.052
	White	0.132	0.052	**0.064**	2.540	0.011	0.938	1.066
	Hispanic	0.014	0.080	0.004	0.173	0.862	0.979	1.021
	Access	-0.047	0.039	-0.035	-1.193	0.233	0.671	1.491
	Knowledge	-0.203	0.091	**-0.079**	-2.237	0.026	0.470	2.126
	StatusQuo	-0.337	0.039	**-0.275**	-8.589	<0.001	0.576	1.735
	GistPrinciples	0.679	0.052	**0.430**	12.993	<0.001	0.540	1.853
	GlobalBenefits	0.313	0.038	**0.250**	8.274	<0.001	0.648	1.544
	GlobalRisks	-0.176	0.045	**-0.105**	-3.902	<0.001	0.820	1.219
	QuantRisk	0.005	0.002	**0.071**	2.503	0.013	0.741	1.350
3	(Constant)	1.087	0.529		2.054	0.040		
	Age	0.041	0.018	**0.057**	2.351	0.019	0.977	1.024
	Sex	0.075	0.054	0.034	1.379	0.168	0.951	1.052
	White	0.102	0.051	**0.049**	1.998	0.046	0.928	1.078
	Hispanic	0.022	0.079	0.007	0.277	0.782	0.979	1.022
	Access	-0.054	0.039	-0.041	-1.406	0.160	0.670	1.493
	Knowledge	-0.186	0.089	**-0.073**	-2.097	0.036	0.470	2.128
	StatusQuo	-0.301	0.039	**-0.246**	-7.724	<0.001	0.561	1.784
	GistPrinciples	0.632	0.052	**0.400**	12.180	<0.001	0.525	1.903
	GlobalBenefits	0.299	0.037	**0.239**	8.053	<0.001	0.645	1.551
	GlobalRisks	-0.170	0.044	**-0.101**	-3.840	<0.001	0.820	1.220
	QuantRisk	0.005	0.002	**0.059**	2.128	0.034	0.737	1.357
	DescriptiveNorm_2items	0.354	0.063	**0.146**	5.582	<0.001	0.824	1.214

a. Dependent Variable: Intentions

Average Injunctive Norms (Friend and Adult)

Model Summary

					Change Statistics				
Model	R	R Square	Adjusted R Square	Std. Error of the Estimate	R Square Change	F Change	df1	df2	Sig. F Change
1	0.380[a]	0.144	0.137	0.94441	0.144	20.046	6	713	<0.001
2	0.762[b]	0.581	0.575	0.66299	0.437	147.758	5	708	<0.001
3	0.782[c]	0.611	0.605	0.63908	0.030	54.966	1	707	<0.001

a. Predictors: (Constant), Knowledge, Age, Hispanic, Sex, White, Access
b. Predictors: (Constant), Knowledge, Age, Hispanic, Sex, White, Access, GlobalRisks, GlobalBenefits, QuantRisk, StatusQuo, GistPrinciples
c. Predictors: (Constant), Knowledge, Age, Hispanic, Sex, White, Access, GlobalRisks, GlobalBenefits, QuantRisk, StatusQuo, GistPrinciples, InjunctiveNorm_2items

Coefficients[a]

		Unstandardized Coefficients		Standardized Coefficients			Collinearity Statistics	
Model		B	Std. Error	Beta	t	Sig.	Tolerance	VIF
1	(Constant)	-0.562	0.588		-0.954	0.340		
	Age	0.035	0.025	0.048	1.386	0.166	0.989	1.011
	Sex	0.113	0.078	0.051	1.444	0.149	0.971	1.029
	White	0.171	0.074	**0.083**	2.329	0.020	0.952	1.050
	Hispanic	0.011	0.114	0.004	0.100	0.920	0.980	1.020
	Access	0.079	0.056	0.060	1.427	0.154	0.683	1.465
	Knowledge	0.781	0.107	**0.306**	7.272	<0.001	0.679	1.473
2	(Constant)	1.321	0.539		2.452	0.014		
	Age	0.032	0.018	0.044	1.781	0.075	0.986	1.014
	Sex	0.078	0.055	0.035	1.408	0.160	0.951	1.052
	White	0.132	0.052	**0.064**	2.540	0.011	0.938	1.066
	Hispanic	0.014	0.080	0.004	0.173	0.862	0.979	1.021
	Access	-0.047	0.039	-0.035	-1.193	0.233	0.671	1.491
	Knowledge	-0.203	0.091	**-0.079**	-2.237	0.026	0.470	2.126
	StatusQuo	-0.337	0.039	**-0.275**	-8.589	<0.001	0.576	1.735
	GistPrinciples	0.679	0.052	**0.430**	12.993	<0.001	0.540	1.853
	GlobalBenefits	0.313	0.038	**0.250**	8.274	<0.001	0.648	1.544
	GlobalRisks	-0.176	0.045	**-0.105**	-3.902	<0.001	0.820	1.219
	QuantRisk	0.005	0.002	**0.071**	2.503	0.013	0.741	1.350

3	(Constant)	0.882	0.523		1.688	0.092		
	Age	0.036	0.017	**0.049**	2.077	0.038	0.985	1.015
	Sex	0.083	0.053	0.037	1.550	0.122	0.951	1.052
	White	0.104	0.050	**0.050**	2.070	0.039	0.933	1.072
	Hispanic	-0.018	0.078	-0.006	-0.236	0.813	0.976	1.024
	Access	-0.055	0.038	-0.041	-1.443	0.149	0.670	1.492
	Knowledge	-0.221	0.087	**-0.087**	-2.537	0.011	0.470	2.128
	StatusQuo	-0.312	0.038	**-0.255**	-8.231	<0.001	0.572	1.748
	GistPrinciples	0.561	0.053	**0.356**	10.626	<0.001	0.491	2.037
	GlobalBenefits	0.248	0.037	**0.198**	6.605	<0.001	0.612	1.634
	GlobalRisks	-0.175	0.044	**-0.104**	-4.011	<0.001	0.820	1.219
	QuantRisk	0.006	0.002	**0.084**	3.086	0.002	0.738	1.356
	InjunctiveNorm_2items	0.274	0.037	**0.222**	7.414	<0.001	0.614	1.629

a. Dependent Variable: Intentions

Logistic Regression for Behavior with Demographics – Including Quantitative and Social Norms

Friend Descriptive

Block 1

Omnibus Tests of Model Coefficients

		Chi-square	df	Sig.
Step 1	Step	28.835	6	.000
	Block	28.835	6	.000
	Model	28.835	6	.000

Model Summary

Step	-2 Log likelihood	Cox & Snell R Square	Nagelkerke R Square
1	962.480[a]	0.039	0.053

a. Estimation terminated at iteration number 3 because parameter estimates changed by less than 0.001.

Variables in the Equation

		B	S.E.	Wald	df	Sig.	Exp(B)	95% C.I.for EXP(B) Lower	Upper
Step 1[a]	Age	0.022	0.056	0.157	1	0.692	1.022	0.917	1.140
	Sex	-0.081	0.170	0.228	1	0.633	0.922	0.661	1.286
	White	0.267	0.159	2.836	1	0.092	1.307	0.957	1.784
	Hispanic	-0.363	0.247	2.157	1	0.142	0.695	0.428	1.129
	Access	0.072	0.120	0.356	1	0.551	1.074	0.849	1.360
	Knowledge	**0.781**	0.236	11.009	1	<0.001	2.185	1.377	3.466
	Constant	-3.441	1.293	7.078	1	0.008	0.032		

a. Variable(s) entered on step 1: Age, Sex, White, Hispanic, Access, Knowledge.

Block 2

Omnibus Tests of Model Coefficients

		Chi-square	df	Sig.
Step 1	Step	167.627	5	<0.001
	Block	167.627	5	<0.001
	Model	196.463	11	<0.001

Model Summary

Step	-2 Log likelihood	Cox & Snell R Square	Nagelkerke R Square
1	794.853[a]	0.239	0.319

a. Estimation terminated at iteration number 5 because parameter estimates changed by less than 0.001.

Variables in the Equation

		B	S.E.	Wald	df	Sig.	Exp(B)	95% C.I.for EXP(B) Lower	Upper
Step 1[a]	Age	0.005	0.062	0.006	1	0.940	1.005	0.890	1.134
	Sex	-0.142	0.193	0.538	1	0.463	0.868	0.595	1.267
	White	0.236	0.180	1.718	1	0.190	1.266	0.890	1.800
	Hispanic	-0.481	0.274	3.084	1	0.079	0.618	0.361	1.057
	Access	-0.134	0.138	0.940	1	0.332	0.875	0.668	1.146
	Knowledge	**-0.824**	0.321	6.588	1	0.010	0.439	0.234	0.823

	StatusQuo	**-0.877**	0.147	35.733	1	<0.001	0.416	0.312	0.555
	GistPrinciples	**1.053**	0.193	29.669	1	<0.001	2.867	1.963	4.189
	GlobalBenefits	**0.398**	0.128	9.646	1	0.002	1.489	1.158	1.915
	GlobalRisks	**-0.475**	0.161	8.726	1	0.003	0.622	0.454	0.852
	QuantRisk	**0.019**	0.008	5.923	1	0.015	1.019	1.004	1.034
	Constant	1.461	1.896	0.593	1	0.441	4.309		

a. Variable(s) entered on step 1: StatusQuo, GistPrinciples, GlobalBenefits, GlobalRisks, QuantRisk.

Block 3

Omnibus Tests of Model Coefficients

		Chi-square	df	Sig.
Step 1	Step	33.881	1	<0.001
	Block	33.881	1	<0.001
	Model	230.344	12	<0.001

Model Summary

Step	-2 Log likelihood	Cox & Snell R Square	Nagelkerke R Square
1	760.972[a]	0.274	0.366

a. Estimation terminated at iteration number 5 because parameter estimates changed by less than 0.001.

Variables in the Equation

								95% C.I.for EXP(B)	
		B	S.E.	Wald	df	Sig.	Exp(B)	Lower	Upper
Step 1[a]	Age	0.040	0.063	0.391	1	0.532	1.040	0.919	1.178
	Sex	-0.140	0.197	0.503	1	0.478	0.869	0.590	1.280
	White	0.159	0.185	0.741	1	0.389	1.173	0.816	1.684
	Hispanic	-0.497	0.282	3.099	1	0.078	0.608	0.350	1.058
	Access	-0.176	0.142	1.531	1	0.216	0.839	0.635	1.108
	Knowledge	**-0.805**	0.330	5.941	1	0.015	0.447	0.234	0.854
	StatusQuo	**-0.819**	0.150	29.806	1	<0.001	0.441	0.329	0.592
	GistPrinciples	**0.991**	0.197	25.324	1	<0.001	2.693	1.831	3.961
	GlobalBenefits	**0.382**	0.133	8.262	1	0.004	1.465	1.129	1.900
	GlobalRisks	**-0.522**	0.167	9.810	1	0.002	0.593	0.428	0.822

		B	S.E.	Wald	df	Sig.	Exp(B)	Lower	Upper
	QuantRisk	**0.017**	0.008	4.748	1	0.029	1.017	1.002	1.033
	FriendDescr	**1.056**	0.184	32.901	1	<0.001	2.876	2.005	4.126
	Constant	0.738	1.949	0.143	1	0.705	2.092		

a. Variable(s) entered on step 1: FriendDescr.

Adult Descriptive

Block 3 (Block 1 and 2 are identical with Friend Descriptive Norms)

Omnibus Tests of Model Coefficients

		Chi-square	df	Sig.
Step 1	Step	29.392	1	<0.001
	Block	29.392	1	<0.001
	Model	225.854	12	<0.001

Model Summary

Step	-2 Log likelihood	Cox & Snell R Square	Nagelkerke R Square
1	765.462[a]	0.269	0.360

a. Estimation terminated at iteration number 5 because parameter estimates changed by less than 0.001.

Variables in the Equation

								95% C.I.for EXP(B)	
		B	S.E.	Wald	df	Sig.	Exp(B)	Lower	Upper
Step 1[a]	Age	0.034	0.063	0.294	1	0.587	1.035	0.914	1.172
	Sex	-0.136	0.196	0.478	1	0.489	0.873	0.594	1.283
	White	0.164	0.184	0.793	1	0.373	1.178	0.821	1.690
	Hispanic	-0.462	0.280	2.721	1	0.099	0.630	0.364	1.091
	Access	-0.136	0.142	0.925	1	0.336	0.872	0.661	1.152
	Knowledge	**-0.825**	0.330	6.256	1	0.012	0.438	0.229	0.836
	StatusQuo	**-0.783**	0.150	27.415	1	<0.001	0.457	0.341	0.613
	GistPrinciples	**0.915**	0.197	21.514	1	<0.001	2.497	1.696	3.675
	GlobalBenefits	**0.357**	0.132	7.376	1	0.007	1.430	1.105	1.850
	GlobalRisks	**-0.457**	0.163	7.833	1	0.005	0.633	0.460	0.872
	QuantRisk	**0.015**	0.008	3.884	1	0.049	1.015	1.000	1.031

		B	S.E.	Wald	df	Sig.	Exp(B)	Lower	Upper
	AdultDescr	**0.988**	0.183	29.194	1	<0.001	2.687	1.877	3.845
	Constant	0.883	1.943	0.206	1	0.650	2.418		

a. Variable(s) entered on step 1: AdultDescr.

Friend Injunctive

Block 3 (Block 1 and 2 are identical with Friend Descriptive Norms)

Omnibus Tests of Model Coefficients

		Chi-square	df	Sig.
Step 1	Step	6.451	1	0.011
	Block	6.451	1	0.011
	Model	202.913	12	<0.001

Model Summary

Step	-2 Log likelihood	Cox & Snell R Square	Nagelkerke R Square
1	788.403[a]	0.246	0.328

a. Estimation terminated at iteration number 5 because parameter estimates changed by less than 0.001.

Variables in the Equation

								95% C.I.for EXP(B)	
		B	S.E.	Wald	df	Sig.	Exp(B)	Lower	Upper
Step 1[a]	Age	-0.002	0.062	0.001	1	0.972	0.998	0.884	1.127
	Sex	-0.142	0.194	0.537	1	0.464	0.868	0.593	1.269
	White	0.199	0.182	1.203	1	0.273	1.220	0.855	1.742
	Hispanic	-0.496	0.275	3.259	1	0.071	0.609	0.356	1.043
	Access	-0.119	0.139	0.728	1	0.393	0.888	0.676	1.166
	Knowledge	**-0.881**	0.324	7.387	1	0.007	0.415	0.220	0.782
	StatusQuo	**-0.860**	0.148	33.902	1	<0.001	0.423	0.317	0.565
	GistPrinciples	**0.959**	0.197	23.756	1	<0.001	2.610	1.775	3.838
	GlobalBenefits	**0.335**	0.131	6.515	1	0.011	1.398	1.081	1.809
	GlobalRisks	**-0.472**	0.162	8.466	1	0.004	0.624	0.454	0.857
	QuantRisk	**0.020**	0.008	6.539	1	0.011	1.020	1.005	1.035
	FriendInjunct	**0.299**	0.118	6.428	1	0.011	1.349	1.070	1.699

		B	S.E.	Wald	df	Sig.	Exp(B)
	Constant	1.135	1.909	0.354	1	0.552	3.112

a. Variable(s) entered on step 1: FriendInjunct.

Adult Injunctive

Block 3 (Block 1 and 2 are identical with Friend Descriptive Norms)

Omnibus Tests of Model Coefficients

		Chi-square	df	Sig.
Step 1	Step	21.453	1	<0.001
	Block	21.453	1	<0.001
	Model	217.915	12	<0.001

Model Summary

Step	-2 Log likelihood	Cox & Snell R Square	Nagelkerke R Square
1	773.400[a]	0.261	0.349

a. Estimation terminated at iteration number 5 because parameter estimates changed by less than 0.001.

Variables in the Equation

								95% C.I.for EXP(B)	
		B	S.E.	Wald	df	Sig.	Exp(B)	Lower	Upper
Step 1[a]	Age	0.028	0.063	0.194	1	0.660	1.028	0.909	1.163
	Sex	-0.109	0.195	0.314	1	0.575	0.897	0.612	1.314
	White	0.197	0.183	1.155	1	0.283	1.217	0.850	1.742
	Hispanic	**-0.606**	0.280	4.702	1	0.030	0.545	0.315	0.943
	Access	-0.190	0.141	1.799	1	0.180	0.827	0.627	1.091
	Knowledge	**-0.842**	0.325	6.714	1	0.010	0.431	0.228	0.814
	StatusQuo	**-0.856**	0.148	33.497	1	<0.001	0.425	0.318	0.568
	GistPrinciples	**0.815**	0.202	16.296	1	<0.001	2.259	1.521	3.356
	GlobalBenefits	**0.281**	0.132	4.519	1	0.034	1.325	1.022	1.717
	GlobalRisks	**-0.480**	0.164	8.555	1	0.003	0.619	0.449	0.854
	QuantRisk	**0.022**	0.008	7.914	1	0.005	1.022	1.007	1.038
	AdultInjunct	**0.512**	0.113	20.544	1	<0.001	1.668	1.337	2.081
	Constant	0.496	1.930	0.066	1	0.797	1.642		

a. Variable(s) entered on step 1: AdultInjunct.

Average Descriptive Norms (Friend and Adult)

Block 3 (Block 1 and 2 are identical with Friend Descriptive Norms)

Omnibus Tests of Model Coefficients

		Chi-square	df	Sig.
Step 1	Step	47.004	1	<0.001
	Block	47.004	1	<0.001
	Model	243.467	12	<0.001

Model Summary

Step	-2 Log likelihood	Cox & Snell R Square	Nagelkerke R Square
1	747.849[a]	0.287	0.384

a. Estimation terminated at iteration number 5 because parameter estimates changed by less than 0.001.

Variables in the Equation

		B	S.E.	Wald	df	Sig.	Exp(B)	95% C.I.for EXP(B)	
								Lower	Upper
Step 1[a]	Age	0.056	0.064	0.748	1	0.387	1.057	0.932	1.199
	Sex	-0.141	0.199	0.500	1	0.479	0.869	0.588	1.283
	White	0.121	0.187	0.418	1	0.518	1.129	0.782	1.629
	Hispanic	-0.482	0.284	2.875	1	0.090	0.618	0.354	1.078
	Access	-0.168	0.144	1.358	1	0.244	0.845	0.637	1.121
	Knowledge	**-0.810**	0.336	5.820	1	0.016	0.445	0.230	0.859
	StatusQuo	**-0.755**	0.151	24.993	1	<0.001	0.470	0.350	0.632
	GistPrinciples	**0.901**	0.199	20.477	1	<0.001	2.463	1.667	3.639
	GlobalBenefits	**0.352**	0.134	6.901	1	0.009	1.422	1.094	1.850
	GlobalRisks	**-0.495**	0.167	8.776	1	0.003	0.610	0.440	0.846
	QuantRisk	0.015	0.008	3.465	1	0.063	1.015	0.999	1.031
	DescriptiveNorm_2items	**1.540**	0.231	44.476	1	<0.001	4.665	2.967	7.335
	Constant	0.437	1.968	0.049	1	0.824	1.547		

a. Variable(s) entered on step 1: DescriptiveNorm_2items.

Average Injunctive Norms (Friend and Adult)

Block 3 (Block 1 and 2 are identical with Friend Descriptive Norms)

Omnibus Tests of Model Coefficients

		Chi-square	df	Sig.
Step 1	Step	18.365	1	<0.001
	Block	18.365	1	<0.001
	Model	214.827	12	<0.001

Model Summary

Step	-2 Log likelihood	Cox & Snell R Square	Nagelkerke R Square
1	776.488[a]	0.258	0.345

a. Estimation terminated at iteration number 5 because parameter estimates changed by less than 0.001.

Variables in the Equation

		B	S.E.	Wald	df	Sig.	Exp(B)	95% C.I.for EXP(B)	
								Lower	Upper
Step 1[a]	Age	0.011	0.062	0.030	1	0.863	1.011	0.894	1.142
	Sex	-0.123	0.195	0.399	1	0.528	0.884	0.603	1.296
	White	0.181	0.183	0.977	1	0.323	1.198	0.837	1.716
	Hispanic	**-0.567**	0.278	4.164	1	0.041	0.567	0.329	0.978
	Access	-0.147	0.140	1.096	1	0.295	0.863	0.656	1.137
	Knowledge	**-0.892**	0.326	7.484	1	0.006	0.410	0.216	0.777
	StatusQuo	**-0.848**	0.149	32.584	1	<0.001	0.428	0.320	0.573
	GistPrinciples	**0.828**	0.202	16.839	1	<0.001	2.288	1.541	3.397
	GlobalBenefits	**0.271**	0.133	4.149	1	0.042	1.312	1.010	1.703
	GlobalRisks	**-0.472**	0.164	8.301	1	0.004	0.624	0.452	0.860
	QuantRisk	**0.022**	0.008	7.619	1	0.006	1.022	1.006	1.038
	InjunctiveNorm_2items	**0.578**	0.137	17.796	1	<0.001	1.783	1.363	2.333
	Constant	0.594	1.929	0.095	1	0.758	1.811		

a. Variable(s) entered on step 1: InjunctiveNorm_2items.

Linear Regression for Intentions WITHOUT Demographics

Friend Descriptive

Model Summary

					Change Statistics				
Model	R	R Square	Adjusted R Square	Std. Error of the Estimate	R Square Change	F Change	df1	df2	Sig. F Change
1	0.365[a]	0.133	0.131	0.94776	0.133	55.203	2	717	<0.001
2	0.758[b]	0.575	0.571	0.66624	0.441	147.786	5	712	<0.001
3	0.761[c]	0.580	0.575	0.66281	0.005	8.406	1	711	0.004

a. Predictors: (Constant), Knowledge, Access
b. Predictors: (Constant), Knowledge, Access, GlobalRisks, GlobalBenefits, QuantRisk, StatusQuo, GistPrinciples
c. Predictors: (Constant), Knowledge, Access, GlobalRisks, GlobalBenefits, QuantRisk, StatusQuo, GistPrinciples, FriendDescr

Coefficients[a]

		Unstandardized Coefficients		Standardized Coefficients			Collinearity Statistics	
Model		B	Std. Error	Beta	t	Sig.	Tolerance	VIF
1	(Constant)	0.106	0.327		0.325	0.745		
	Access	0.093	0.055	0.070	1.679	0.094	0.693	1.442
	Knowledge	0.822	0.107	**0.322**	7.708	<0.001	0.693	1.442
2	(Constant)	1.933	0.417		4.641	<0.001		
	Access	-0.038	0.039	-0.029	-0.976	0.330	0.681	1.469
	Knowledge	-0.169	0.090	**-0.066**	-1.876	0.061	0.478	2.090
	StatusQuo	-0.346	0.039	**-0.283**	-8.814	<0.001	0.581	1.721
	GistPrinciples	0.673	0.052	**0.426**	12.835	<0.001	0.542	1.847
	GlobalBenefits	0.317	0.038	**0.254**	8.396	<0.001	0.654	1.529
	GlobalRisks	-0.173	0.045	**-0.102**	-3.817	<0.001	0.829	1.207
	QuantRisk	0.006	0.002	**0.079**	2.782	0.006	0.747	1.339
3	(Constant)	1.895	0.415		4.569	<0.001		
	Access	-0.045	0.039	-0.034	-1.140	0.255	0.679	1.474
	Knowledge	-0.159	0.090	-0.062	-1.772	0.077	0.478	2.093

StatusQuo	-0.331	0.039	**-0.270**	-8.409	<0.001	0.571	1.751
GistPrinciples	0.657	0.052	**0.416**	12.531	<0.001	0.536	1.867
GlobalBenefits	0.313	0.038	**0.250**	8.312	<0.001	0.653	1.531
GlobalRisks	-0.172	0.045	**-0.102**	-3.830	<0.001	0.829	1.207
QuantRisk	0.006	0.002	**0.075**	2.669	0.008	0.745	1.342
FriendDescr	0.151	0.052	**0.074**	2.899	0.004	0.919	1.088

a. Dependent Variable: Intentions

Adult Descriptive

Model Summary

					Change Statistics				
Model	R	R Square	Adjusted R Square	Std. Error of the Estimate	R Square Change	F Change	df1	df2	Sig. F Change
1	0.365[a]	0.133	0.131	0.94776	0.133	55.203	2	717	<0.001
2	0.758[b]	0.575	0.571	0.66624	0.441	147.786	5	712	<0.001
3	0.773[c]	0.598	0.593	0.64831	0.023	40.936	1	711	<0.001

a. Predictors: (Constant), Knowledge, Access
b. Predictors: (Constant), Knowledge, Access, GlobalRisks, GlobalBenefits, QuantRisk, StatusQuo, GistPrinciples
c. Predictors: (Constant), Knowledge, Access, GlobalRisks, GlobalBenefits, QuantRisk, StatusQuo, GistPrinciples, AdultDescr

Coefficients[a]

		Unstandardized Coefficients		Standardized Coefficients			Collinearity Statistics	
Model		B	Std. Error	Beta	t	Sig.	Tolerance	VIF
1	(Constant)	0.106	0.327		0.325	0.745		
	Access	0.093	0.055	0.070	1.679	0.094	0.693	1.442
	Knowledge	0.822	0.107	**0.322**	7.708	<0.001	0.693	1.442
2	(Constant)	1.933	0.417		4.641	<0.001		
	Access	-0.038	0.039	-0.029	-0.976	0.330	0.681	1.469
	Knowledge	-0.169	0.090	-0.066	-1.876	0.061	0.478	2.090
	StatusQuo	-0.346	0.039	**-0.283**	-8.814	<0.001	0.581	1.721
	GistPrinciples	0.673	0.052	**0.426**	12.835	<0.001	0.542	1.847
	GlobalBenefits	0.317	0.038	**0.254**	8.396	<0.001	0.654	1.529
	GlobalRisks	-0.173	0.045	**-0.102**	-3.817	<0.001	0.829	1.207

	QuantRisk	0.006	0.002	**0.079**	2.782	0.006	0.747	1.339
3	(Constant)	1.921	0.405		4.740	<0.001		
	Access	-0.042	0.038	-0.032	-1.097	0.273	0.680	1.470
	Knowledge	-0.168	0.088	-0.066	-1.911	0.056	0.478	2.090
	StatusQuo	-0.308	0.039	**-0.252**	-7.966	<0.001	0.567	1.763
	GistPrinciples	0.622	0.052	**0.394**	12.061	<0.001	0.529	1.891
	GlobalBenefits	0.299	0.037	**0.239**	8.092	<0.001	0.650	1.538
	GlobalRisks	-0.160	0.044	**-0.095**	-3.644	<0.001	0.827	1.209
	QuantRisk	0.005	0.002	**0.063**	2.272	0.023	0.741	1.350
	AdultDescr	0.335	0.052	**0.165**	6.398	<0.001	0.850	1.177

a. Dependent Variable: Intentions

Friend Injunctive

Model Summary

					Change Statistics				
Model	R	R Square	Adjusted R Square	Std. Error of the Estimate	R Square Change	F Change	df1	df2	Sig. F Change
1	0.365[a]	0.133	0.131	0.94776	0.133	55.203	2	717	<0.001
2	0.758[b]	0.575	0.571	0.66624	0.441	147.786	5	712	<0.001
3	0.764[c]	0.583	0.579	0.65989	0.009	14.767	1	711	<0.001

a. Predictors: (Constant), Knowledge, Access

b. Predictors: (Constant), Knowledge, Access, GlobalRisks, GlobalBenefits, QuantRisk, StatusQuo, GistPrinciples

c. Predictors: (Constant), Knowledge, Access, GlobalRisks, GlobalBenefits, QuantRisk, StatusQuo, GistPrinciples, FriendInjunct

Coefficients[a]

		Unstandardized Coefficients		Standardized Coefficients			Collinearity Statistics	
Model		B	Std. Error	Beta	t	Sig.	Tolerance	VIF
1	(Constant)	0.106	0.327		0.325	0.745		
	Access	0.093	0.055	0.070	1.679	0.094	0.693	1.442
	Knowledge	0.822	0.107	**0.322**	7.708	<0.001	0.693	1.442
2	(Constant)	1.933	0.417		4.641	<0.001		
	Access	-0.038	0.039	-0.029	-0.976	0.330	0.681	1.469
	Knowledge	-0.169	0.090	-0.066	-1.876	0.061	0.478	2.090
	StatusQuo	-0.346	0.039	**-0.283**	-8.814	<0.001	0.581	1.721
	GistPrinciples	0.673	0.052	**0.426**	12.835	<0.001	0.542	1.847

Model		B	Std. Error	Beta	t	Sig.	Tolerance	VIF
	GlobalBenefits	0.317	0.038	**0.254**	8.396	<0.001	0.654	1.529
	GlobalRisks	-0.173	0.045	**-0.102**	-3.817	<0.001	0.829	1.207
	QuantRisk	0.006	0.002	**0.079**	2.782	0.006	0.747	1.339
3	(Constant)	1.723	0.416		4.139	<0.001		
	Access	-0.034	0.039	-0.025	-0.863	0.388	0.680	1.471
	Knowledge	-0.190	0.089	**-0.074**	-2.118	0.035	0.477	2.097
	StatusQuo	-0.335	0.039	**-0.274**	-8.598	<0.001	0.578	1.730
	GistPrinciples	0.632	0.053	**0.400**	11.917	<0.001	0.519	1.925
	GlobalBenefits	0.290	0.038	**0.232**	7.613	<0.001	0.631	1.584
	GlobalRisks	-0.170	0.045	**-0.101**	-3.786	<0.001	0.828	1.207
	QuantRisk	0.006	0.002	**0.084**	2.982	0.003	0.745	1.342
	FriendInjunct	0.125	0.033	**0.107**	3.843	<0.001	0.753	1.328

a. Dependent Variable: Intentions

Adult Injunctive

Model Summary

					Change Statistics				
Model	R	R Square	Adjusted R Square	Std. Error of the Estimate	R Square Change	F Change	df1	df2	Sig. F Change
1	0.365[a]	0.133	0.131	0.94776	0.133	55.203	2	717	<0.001
2	0.758[b]	0.575	0.571	0.66624	0.441	147.786	5	712	<0.001
3	0.784[c]	0.615	0.610	0.63469	0.040	73.556	1	711	<0.001

a. Predictors: (Constant), Knowledge, Access
b. Predictors: (Constant), Knowledge, Access, GlobalRisks, GlobalBenefits, QuantRisk, StatusQuo, GistPrinciples
c. Predictors: (Constant), Knowledge, Access, GlobalRisks, GlobalBenefits, QuantRisk, StatusQuo, GistPrinciples, AdultInjunct

Coefficients[a]

		Unstandardized Coefficients		Standardized Coefficients			Collinearity Statistics	
Model		B	Std. Error	Beta	t	Sig.	Tolerance	VIF
1	(Constant)	0.106	0.327		0.325	0.745		
	Access	0.093	0.055	0.070	1.679	0.094	0.693	1.442
	Knowledge	0.822	0.107	**0.322**	7.708	<0.001	0.693	1.442
2	(Constant)	1.933	0.417		4.641	<0.001		
	Access	-0.038	0.039	-0.029	-0.976	0.330	0.681	1.469

	Knowledge	-0.169	0.090	-0.066	-1.876	0.061	0.478	2.090
	StatusQuo	-0.346	0.039	**-0.283**	-8.814	<0.001	0.581	1.721
	GistPrinciples	0.673	0.052	**0.426**	12.835	<0.001	0.542	1.847
	GlobalBenefits	0.317	0.038	**0.254**	8.396	<0.001	0.654	1.529
	GlobalRisks	-0.173	0.045	**-0.102**	-3.817	<0.001	0.829	1.207
	QuantRisk	0.006	0.002	**0.079**	2.782	0.006	0.747	1.339
3	(Constant)	1.643	0.398		4.125	<0.001		
	Access	-0.063	0.038	-0.047	-1.674	0.095	0.677	1.478
	Knowledge	-0.165	0.086	-0.065	-1.923	0.055	0.478	2.090
	StatusQuo	-0.318	0.038	**-0.260**	-8.486	<0.001	0.577	1.734
	GistPrinciples	0.540	0.052	**0.342**	10.337	<0.001	0.494	2.023
	GlobalBenefits	0.251	0.037	**0.200**	6.806	<0.001	0.625	1.600
	GlobalRisks	-0.173	0.043	**-0.103**	-4.015	<0.001	0.829	1.207
	QuantRisk	0.007	0.002	**0.092**	3.409	<0.001	0.744	1.344
	AdultInjunct	0.257	0.030	**0.248**	8.576	<0.001	0.647	1.546

a. Dependent Variable: Intentions

Logistic Regression for Behavior WITHOUT Demographics

Friend Descriptive

Block 1

Omnibus Tests of Model Coefficients

		Chi-square	df	Sig.
Step 1	Step	23.943	2	<0.001
	Block	23.943	2	<0.001
	Model	23.943	2	<0.001

Model Summary

Step	-2 Log likelihood	Cox & Snell R Square	Nagelkerke R Square
1	967.373[a]	0.033	0.044

a. Estimation terminated at iteration number 3 because parameter estimates changed by less than 0.001.

Variables in the Equation

		B	S.E.	Wald	df	Sig.	Exp(B)	95% C.I.for EXP(B) Lower	Upper
Step 1[a]	Access	0.086	0.119	0.525	1	0.469	1.090	0.863	1.376
	Knowledge	**0.838**	0.233	12.966	1	<0.001	2.311	1.465	3.646
	Constant	-3.212	0.720	19.880	1	<0.001	0.040		

a. Variable(s) entered on step 1: Access, Knowledge.

Block 2

Omnibus Tests of Model Coefficients

		Chi-square	df	Sig.
Step 1	Step	167.626	5	<0.001
	Block	167.626	5	<0.001
	Model	191.568	7	<0.001

Model Summary

Step	-2 Log likelihood	Cox & Snell R Square	Nagelkerke R Square
1	799.747[a]	0.234	0.312

a. Estimation terminated at iteration number 5 because parameter estimates changed by less than 0.001.

Variables in the Equation

		B	S.E.	Wald	df	Sig.	Exp(B)	95% C.I.for EXP(B) Lower	Upper
Step 1[a]	Access	-0.125	0.136	0.838	1	0.360	0.883	0.676	1.153
	Knowledge	**-0.753**	0.316	5.700	1	0.017	0.471	0.254	0.874
	StatusQuo	**-0.880**	0.145	36.670	1	<0.001	0.415	0.312	0.551
	GistPrinciples	**1.040**	0.191	29.628	1	<0.001	2.830	1.946	4.116
	GlobalBenefits	**0.387**	0.127	9.266	1	0.002	1.473	1.148	1.891
	GlobalRisks	**-0.478**	0.159	9.095	1	0.003	0.620	0.454	0.846
	QuantRisk	**0.019**	0.008	6.331	1	0.012	1.019	1.004	1.034
	Constant	1.332	1.461	0.832	1	0.362	3.789		

a. Variable(s) entered on step 1: StatusQuo, GistPrinciples, GlobalBenefits, GlobalRisks, QuantRisk.

Block 3

Omnibus Tests of Model Coefficients

		Chi-square	df	Sig.
Step 1	Step	34.384	1	<0.001
	Block	34.384	1	<0.001
	Model	225.952	8	<0.001

Model Summary

Step	-2 Log likelihood	Cox & Snell R Square	Nagelkerke R Square
1	765.363[a]	0.269	0.360

a. Estimation terminated at iteration number 5 because parameter estimates changed by less than 0.001.

Variables in the Equation

		B	S.E.	Wald	df	Sig.	Exp(B)	95% C.I.for EXP(B)	
								Lower	Upper
Step 1[a]	Access	-0.171	0.141	1.473	1	0.225	0.843	0.640	1.111
	Knowledge	**-0.742**	0.325	5.217	1	0.022	0.476	0.252	0.900
	StatusQuo	**-0.818**	0.149	30.221	1	<0.001	0.441	0.329	0.591
	GistPrinciples	**0.988**	0.195	25.617	1	<0.001	2.685	1.832	3.936
	GlobalBenefits	**0.370**	0.132	7.872	1	0.005	1.447	1.118	1.873
	GlobalRisks	**-0.521**	0.164	10.066	1	0.002	0.594	0.430	0.819
	QuantRisk	**0.018**	0.008	5.108	1	0.024	1.018	1.002	1.033
	FriendDescr	**1.052**	0.182	33.408	1	<0.001	2.864	2.005	4.092
	Constant	1.245	1.499	0.689	1	0.406	3.472		

a. Variable(s) entered on step 1: FriendDescr.

Adult Descriptive

Block 3 (Block 1 and 2 identical to Friend Descriptive)

Omnibus Tests of Model Coefficients

		Chi-square	df	Sig.

Step 1	Step	30.289	1	<0.001
	Block	30.289	1	<0.001
	Model	221.858	8	<0.001

Model Summary

Step	-2 Log likelihood	Cox & Snell R Square	Nagelkerke R Square
1	769.458[a]	0.265	0.355

a. Estimation terminated at iteration number 5 because parameter estimates changed by less than 0.001.

Variables in the Equation

		B	S.E.	Wald	df	Sig.	Exp(B)	95% C.I.for EXP(B)	
								Lower	Upper
Step 1[a]	Access	-0.129	0.140	0.852	1	0.356	0.879	0.668	1.156
	Knowledge	**-0.767**	0.325	5.577	1	0.018	0.464	0.246	0.878
	StatusQuo	**-0.781**	0.148	27.728	1	<0.001	0.458	0.342	0.612
	GistPrinciples	**0.913**	0.195	21.816	1	<0.001	2.491	1.699	3.654
	GlobalBenefits	**0.345**	0.130	6.987	1	0.008	1.411	1.093	1.822
	GlobalRisks	**-0.460**	0.161	8.168	1	0.004	0.631	0.460	0.865
	QuantRisk	**0.016**	0.008	4.277	1	0.039	1.016	1.001	1.031
	AdultDescr	**0.993**	0.181	30.115	1	<0.001	2.699	1.893	3.848
	Constant	1.301	1.496	0.756	1	0.385	3.672		

a. Variable(s) entered on step 1: AdultDescr.

Friend Injunctive

Block 3 (Block 1 and 2 identical to Friend Descriptive)

Omnibus Tests of Model Coefficients

		Chi-square	df	Sig.
Step 1	Step	6.729	1	0.009
	Block	6.729	1	0.009
	Model	198.298	8	<0.001

Model Summary

Step	-2 Log likelihood	Cox & Snell R Square	Nagelkerke R Square
1	793.018[a]	0.241	0.322

a. Estimation terminated at iteration number 5 because parameter estimates changed by less than 0.001.

Variables in the Equation

		B	S.E.	Wald	df	Sig.	Exp(B)	95% C.I.for EXP(B)	
								Lower	Upper
Step 1[a]	Access	-0.110	0.137	0.645	1	0.422	0.896	0.684	1.172
	Knowledge	**-0.814**	0.318	6.543	1	0.011	0.443	0.237	0.827
	StatusQuo	**-0.861**	0.146	34.567	1	<0.001	0.423	0.317	0.563
	GistPrinciples	**0.946**	0.195	23.621	1	<0.001	2.575	1.758	3.770
	GlobalBenefits	**0.324**	0.130	6.165	1	0.013	1.382	1.071	1.785
	GlobalRisks	**-0.479**	0.160	8.954	1	0.003	0.620	0.453	0.848
	QuantRisk	**0.020**	0.008	6.990	1	0.008	1.020	1.005	1.036
	FriendInjunct	**0.302**	0.117	6.696	1	0.010	1.353	1.076	1.701
	Constant	0.863	1.481	0.339	1	0.560	2.369		

a. Variable(s) entered on step 1: FriendInjunct.

Adult Injunctive

Block 3 (Block 1 and 2 identical to Friend Descriptive)

Omnibus Tests of Model Coefficients

		Chi-square	df	Sig.
Step 1	Step	20.394	1	<0.001
	Block	20.394	1	<0.001
	Model	211.962	8	<0.001

Model Summary

Step	-2 Log likelihood	Cox & Snell R Square	Nagelkerke R Square
1	779.354[a]	0.255	0.341

a. Estimation terminated at iteration number 5 because parameter estimates changed by less than 0.001.

Variables in the Equation

		B	S.E.	Wald	df	Sig.	Exp(B)	95% C.I.for EXP(B) Lower	Upper
Step 1[a]	Access	-0.173	0.139	1.533	1	0.216	0.842	0.640	1.106
	Knowledge	**-0.768**	0.320	5.767	1	0.016	0.464	0.248	0.868
	StatusQuo	**-0.857**	0.147	34.093	1	<0.001	0.424	0.318	0.566
	GistPrinciples	**0.815**	0.199	16.780	1	<0.001	2.259	1.530	3.336
	GlobalBenefits	**0.274**	0.131	4.346	1	0.037	1.315	1.017	1.701
	GlobalRisks	**-0.480**	0.161	8.900	1	0.003	0.619	0.451	0.848
	QuantRisk	**0.022**	0.008	8.430	1	0.004	1.023	1.007	1.038
	AdultInjunct	**0.492**	0.111	19.588	1	<0.001	1.635	1.315	2.032
	Constant	0.764	1.478	0.267	1	0.605	2.148		

a. Variable(s) entered on step 1: AdultInjunct.

Linear Regression for Intentions (Only FTT Predictors)

Model Summary

					Change Statistics				
Model	R	R Square	Adjusted R Square	Std. Error of the Estimate	R Square Change	F Change	df1	df2	Sig. F Change
1	0.755[a]	0.571	0.568	0.66855	0.571	189.759	5	714	<0.001

a. Predictors: (Constant), QR_get_sick_from_flu_vac_mean_Scale_11, GlobalBenefits, GlobalRisks, StatusQuo, GistPrinciples

ANOVA[a]

Model		Sum of Squares	df	Mean Square	F	Sig.
1	Regression	424.079	5	84.816	189.759	<0.001[b]
	Residual	319.133	714	0.447		
	Total	743.212	719			

a. Dependent Variable: Intentions
b. Predictors: (Constant), QR_get_sick_from_flu_vac_mean_Scale_11, GlobalBenefits, GlobalRisks, StatusQuo, GistPrinciples

Coefficients[a]

Model		Unstandardized Coefficients B	Std. Error	Standardized Coefficients Beta	t	Sig.	Collinearity Statistics Tolerance	VIF
1	(Constant)	1.090	0.249		4.369	<0.001		
	StatusQuo	-0.305	0.036	**-0.249**	-8.450	<0.001	0.691	1.446
	GistPrinciples	0.658	0.052	**0.417**	12.583	<0.001	0.548	1.824
	GlobalBenefits	0.308	0.038	**0.246**	8.156	<0.001	0.660	1.515
	GlobalRisks	-0.157	0.045	**-0.093**	-3.499	<0.001	0.844	1.185
	QR_get_sick_from_flu_vac_mean_Scale_11	0.008	0.002	**0.099**	3.646	<0.001	0.815	1.227

a. Dependent Variable: Intentions

Logistic Regression for Behavior (Only FTT Predictors)

Block 1: Method = Enter

Omnibus Tests of Model Coefficients

		Chi-square	df	Sig.
Step 1	Step	182.044	5	<0.001
	Block	182.044	5	<0.001
	Model	182.044	5	<0.001

Model Summary

Step	-2 Log likelihood	Cox & Snell R Square	Nagelkerke R Square
1	809.271[a]	0.223	0.299

a. Estimation terminated at iteration number 4 because parameter estimates changed by less than 0.001.

Variables in the Equation

		B	S.E.	Wald	df	Sig.	Exp(B)	95% C.I.for EXP(B) Lower	Upper
Step 1[a]	StatusQuo	**-0.686**	0.126	29.514	1	<0.001	0.503	0.393	0.645
	GistPrinciples	**0.966**	0.188	26.376	1	<0.001	2.627	1.817	3.797
	GlobalBenefits	**0.341**	0.126	7.330	1	0.007	1.407	1.099	1.801
	GlobalRisks	**-0.405**	0.155	6.803	1	0.009	0.667	0.492	0.904
	QR_get_sick_from_flu_vac_mean_Scale_11	**0.026**	0.007	12.764	1	<0.001	1.026	1.012	1.040

Constant	-2.218	0.851	6.787	1	0.009	0.109

a. Variable(s) entered on step 1: StatusQuo, GistPrinciples, GlobalBenefits, GlobalRisks, QR_get_sick_from_flu_vac_mean_Scale_11.

Linear Regression for Intentions Without Quantitative Risk (Friend Descriptive Norms)

Model Summary

					Change Statistics				
Model	R	R Square	Adjusted R Square	Std. Error of the Estimate	R Square Change	F Change	df1	df2	Sig. F Change
1	0.380[a]	0.144	0.137	0.94441	0.144	20.046	6	713	<0.001
2	0.760[b]	0.578	0.572	0.66544	0.433	181.780	4	709	<0.001
3	0.763[c]	0.583	0.576	0.66192	0.005	8.561	1	708	0.004

a. Predictors: (Constant), Knowledge, Age, Hispanic, Sex, White, Access
b. Predictors: (Constant), Knowledge, Age, Hispanic, Sex, White, Access, GlobalRisks, GlobalBenefits, StatusQuo, GistPrinciples
c. Predictors: (Constant), Knowledge, Age, Hispanic, Sex, White, Access, GlobalRisks, GlobalBenefits, StatusQuo, GistPrinciples, FriendDescr

ANOVA[a]

Model		Sum of Squares	df	Mean Square	F	Sig.
1	Regression	107.275	6	17.879	20.046	<0.001[b]
	Residual	635.938	713	0.892		
	Total	743.212	719			
2	Regression	429.256	10	42.926	96.938	<0.001[c]
	Residual	313.957	709	0.443		
	Total	743.212	719			
3	Regression	433.007	11	39.364	89.843	<0.001[d]
	Residual	310.205	708	0.438		
	Total	743.212	719			

a. Dependent Variable: Intentions
b. Predictors: (Constant), Knowledge, Age, Hispanic, Sex, White, Access
c. Predictors: (Constant), Knowledge, Age, Hispanic, Sex, White, Access, GlobalRisks, GlobalBenefits, StatusQuo, GistPrinciples
d. Predictors: (Constant), Knowledge, Age, Hispanic, Sex, White, Access, GlobalRisks, GlobalBenefits, StatusQuo, GistPrinciples, FriendDescr

Coefficients[a]

Model		Unstandardized Coefficients		Standardized Coefficients			Collinearity Statistics	
		B	Std. Error	Beta	t	Sig.	Tolerance	VIF
1	(Constant)	-0.562	0.588		-0.954	0.340		
	Age	0.035	0.025	0.048	1.386	0.166	0.989	1.011
	Sex	0.113	0.078	0.051	1.444	0.149	0.971	1.029
	White	0.171	0.074	**0.083**	2.329	0.020	0.952	1.050
	Hispanic	0.011	0.114	0.004	0.100	0.920	0.980	1.020
	Access	0.079	0.056	0.060	1.427	0.154	0.683	1.465
	Knowledge	0.781	0.107	**0.306**	7.272	<0.001	0.679	1.473
2	(Constant)	1.608	0.528		3.043	0.002		
	Age	0.033	0.018	0.046	1.864	0.063	0.987	1.013
	Sex	0.082	0.056	0.037	1.470	0.142	0.951	1.051
	White	0.142	0.052	**0.069**	2.730	0.006	0.944	1.060
	Hispanic	0.014	0.081	0.004	0.176	0.860	0.979	1.021
	Access	-0.049	0.040	-0.037	-1.232	0.218	0.671	1.490
	Knowledge	-0.263	0.088	**-0.103**	-3.007	0.003	0.507	1.973
	StatusQuo	-0.343	0.039	**-0.281**	-8.745	<0.001	0.579	1.727
	GistPrinciples	0.668	0.052	**0.423**	12.782	<0.001	0.543	1.840
	GlobalBenefits	0.308	0.038	**0.246**	8.132	<0.001	0.649	1.541
	GlobalRisks	-0.148	0.044	**-0.088**	-3.363	<0.001	0.877	1.140
3	(Constant)	1.473	0.528		2.792	0.005		
	Age	0.038	0.018	**0.052**	2.106	0.036	0.980	1.020
	Sex	0.080	0.055	0.036	1.445	0.149	0.951	1.051
	White	0.129	0.052	**0.062**	2.479	0.013	0.937	1.068
	Hispanic	0.016	0.080	0.005	0.202	0.840	0.979	1.021
	Access	-0.054	0.039	-0.041	-1.376	0.169	0.669	1.494
	Knowledge	-0.249	0.087	**-0.097**	-2.852	0.004	0.505	1.980
	StatusQuo	-0.329	0.039	**-0.269**	-8.347	<0.001	0.570	1.756
	GistPrinciples	0.651	0.052	**0.413**	12.461	<0.001	0.537	1.862
	GlobalBenefits	0.304	0.038	**0.243**	8.069	<0.001	0.648	1.543
	GlobalRisks	-0.149	0.044	**-0.088**	-3.408	<0.001	0.877	1.140
	FriendDescr	0.153	0.052	**0.075**	2.926	0.004	0.906	1.104

a. Dependent Variable: Intentions

Linear Regression for Intentions Without Quantitative Risk (Adult Descriptive Norms)

Model Summary

Model	R	R Square	Adjusted R Square	Std. Error of the Estimate	Change Statistics R Square Change	F Change	df1	df2	Sig. F Change
1	0.380[a]	0.144	0.137	0.94441	0.144	20.046	6	713	<0.001
2	0.760[b]	0.578	0.572	0.66544	0.433	181.780	4	709	<0.001
3	0.776[c]	0.602	0.596	0.64659	0.024	42.941	1	708	<0.001

a. Predictors: (Constant), Knowledge, Age, Hispanic, Sex, White, Access
b. Predictors: (Constant), Knowledge, Age, Hispanic, Sex, White, Access, GlobalRisks, GlobalBenefits, StatusQuo, GistPrinciples
c. Predictors: (Constant), Knowledge, Age, Hispanic, Sex, White, Access, GlobalRisks, GlobalBenefits, StatusQuo, GistPrinciples, AdultDescr

ANOVA[a]

Model		Sum of Squares	df	Mean Square	F	Sig.
1	Regression	107.275	6	17.879	20.046	<0.001[b]
	Residual	635.938	713	0.892		
	Total	743.212	719			
2	Regression	429.256	10	42.926	96.938	<0.001[c]
	Residual	313.957	709	0.443		
	Total	743.212	719			
3	Regression	447.209	11	40.655	97.242	<0.001[d]
	Residual	296.003	708	0.418		
	Total	743.212	719			

a. Dependent Variable: Intentions
b. Predictors: (Constant), Knowledge, Age, Hispanic, Sex, White, Access
c. Predictors: (Constant), Knowledge, Age, Hispanic, Sex, White, Access, GlobalRisks, GlobalBenefits, StatusQuo, GistPrinciples
d. Predictors: (Constant), Knowledge, Age, Hispanic, Sex, White, Access, GlobalRisks, GlobalBenefits, StatusQuo, GistPrinciples, AdultDescr

Coefficients[a]

Model		Unstandardized Coefficients B	Std. Error	Standardized Coefficients Beta	t	Sig.	Collinearity Statistics Tolerance	VIF
1	(Constant)	-0.562	0.588		-0.954	0.340		
	Age	0.035	0.025	0.048	1.386	0.166	0.989	1.011

	Sex	0.113	0.078	0.051	1.444	0.149	0.971	1.029
	White	0.171	0.074	**0.083**	2.329	0.020	0.952	1.050
	Hispanic	0.011	0.114	0.004	0.100	0.920	0.980	1.020
	Access	0.079	0.056	0.060	1.427	0.154	0.683	1.465
	Knowledge	0.781	0.107	**0.306**	7.272	<0.001	0.679	1.473
2	(Constant)	1.608	0.528		3.043	0.002		
	Age	0.033	0.018	0.046	1.864	0.063	0.987	1.013
	Sex	0.082	0.056	0.037	1.470	0.142	0.951	1.051
	White	0.142	0.052	**0.069**	2.730	0.006	0.944	1.060
	Hispanic	0.014	0.081	0.004	0.176	0.860	0.979	1.021
	Access	-0.049	0.040	-0.037	-1.232	0.218	0.671	1.490
	Knowledge	-0.263	0.088	**-0.103**	-3.007	0.003	0.507	1.973
	StatusQuo	-0.343	0.039	**-0.281**	-8.745	<0.001	0.579	1.727
	GistPrinciples	0.668	0.052	**0.423**	12.782	<0.001	0.543	1.840
	GlobalBenefits	0.308	0.038	**0.246**	8.132	<0.001	0.649	1.541
	GlobalRisks	-0.148	0.044	**-0.088**	-3.363	<0.001	0.877	1.140
3	(Constant)	1.364	0.515		2.650	0.008		
	Age	0.042	0.017	**0.057**	2.391	0.017	0.982	1.019
	Sex	0.078	0.054	0.035	1.453	0.147	0.951	1.051
	White	0.111	0.051	**0.054**	2.184	0.029	0.935	1.069
	Hispanic	0.025	0.078	0.008	0.320	0.749	0.979	1.022
	Access	-0.050	0.038	-0.038	-1.303	0.193	0.671	1.490
	Knowledge	-0.244	0.085	**-0.096**	-2.872	0.004	0.506	1.975
	StatusQuo	-0.304	0.039	**-0.249**	-7.881	<0.001	0.565	1.769
	GistPrinciples	0.617	0.051	**0.391**	12.008	<0.001	0.531	1.884
	GlobalBenefits	0.291	0.037	**0.233**	7.886	<0.001	0.646	1.548
	GlobalRisks	-0.142	0.043	**-0.084**	-3.326	<0.001	0.877	1.141
	AdultDescr	0.344	0.052	**0.169**	6.553	<0.001	0.843	1.186

a. Dependent Variable: Intentions

Linear Regression for Intentions Without Quantitative Risk (Friend Injunctive Norms)

Model Summary

					Change Statistics				
Model	R	R Square	Adjusted R Square	Std. Error of the Estimate	R Square Change	F Change	df1	df2	Sig. F Change
1	0.380[a]	0.144	0.137	0.94441	0.144	20.046	6	713	<0.001
2	0.760[b]	0.578	0.572	0.66544	0.433	181.780	4	709	<0.001

3	0.765[c]	0.585	0.578	0.66032	0.007	12.041	1	708	<0.001

a. Predictors: (Constant), Knowledge, Age, Hispanic, Sex, White, Access
b. Predictors: (Constant), Knowledge, Age, Hispanic, Sex, White, Access, GlobalRisks, GlobalBenefits, StatusQuo, GistPrinciples
c. Predictors: (Constant), Knowledge, Age, Hispanic, Sex, White, Access, GlobalRisks, GlobalBenefits, StatusQuo, GistPrinciples, FriendInjunct

ANOVA[a]

Model		Sum of Squares	df	Mean Square	F	Sig.
1	Regression	107.275	6	17.879	20.046	<0.001[b]
	Residual	635.938	713	0.892		
	Total	743.212	719			
2	Regression	429.256	10	42.926	96.938	<0.001[c]
	Residual	313.957	709	0.443		
	Total	743.212	719			
3	Regression	434.506	11	39.501	90.592	<0.001[d]
	Residual	308.706	708	0.436		
	Total	743.212	719			

a. Dependent Variable: Intentions
b. Predictors: (Constant), Knowledge, Age, Hispanic, Sex, White, Access
c. Predictors: (Constant), Knowledge, Age, Hispanic, Sex, White, Access, GlobalRisks, GlobalBenefits, StatusQuo, GistPrinciples
d. Predictors: (Constant), Knowledge, Age, Hispanic, Sex, White, Access, GlobalRisks, GlobalBenefits, StatusQuo, GistPrinciples, FriendInjunct

Coefficients[a]

		Unstandardized Coefficients		Standardized Coefficients			Collinearity Statistics	
Model		B	Std. Error	Beta	t	Sig.	Tolerance	VIF
1	(Constant)	-0.562	0.588		-0.954	0.340		
	Age	0.035	0.025	0.048	1.386	0.166	0.989	1.011
	Sex	0.113	0.078	0.051	1.444	0.149	0.971	1.029
	White	0.171	0.074	**0.083**	2.329	0.020	0.952	1.050
	Hispanic	0.011	0.114	0.004	0.100	0.920	0.980	1.020
	Access	0.079	0.056	0.060	1.427	0.154	0.683	1.465
	Knowledge	0.781	0.107	**0.306**	7.272	<0.001	0.679	1.473
2	(Constant)	1.608	0.528		3.043	0.002		

	Age	0.033	0.018	0.046	1.864	0.063	0.987	1.013
	Sex	0.082	0.056	0.037	1.470	0.142	0.951	1.051
	White	0.142	0.052	**0.069**	2.730	0.006	0.944	1.060
	Hispanic	0.014	0.081	0.004	0.176	0.860	0.979	1.021
	Access	-0.049	0.040	-0.037	-1.232	0.218	0.671	1.490
	Knowledge	-0.263	0.088	**-0.103**	-3.007	0.003	0.507	1.973
	StatusQuo	-0.343	0.039	**-0.281**	-8.745	<0.001	0.579	1.727
	GistPrinciples	0.668	0.052	**0.423**	12.782	<0.001	0.543	1.840
	GlobalBenefits	0.308	0.038	**0.246**	8.132	<0.001	0.649	1.541
	GlobalRisks	-0.148	0.044	**-0.088**	-3.363	<0.001	0.877	1.140
3	(Constant)	1.489	0.525		2.833	0.005		
	Age	0.031	0.018	0.043	1.744	0.082	0.986	1.015
	Sex	0.080	0.055	0.036	1.455	0.146	0.951	1.051
	White	0.131	0.052	**0.063**	2.524	0.012	0.940	1.064
	Hispanic	0.010	0.080	0.003	0.126	0.900	0.979	1.021
	Access	-0.044	0.039	-0.033	-1.126	0.261	0.670	1.492
	Knowledge	-0.284	0.087	**-0.111**	-3.259	0.001	0.504	1.982
	StatusQuo	-0.335	0.039	**-0.274**	-8.579	<0.001	0.577	1.734
	GistPrinciples	0.629	0.053	**0.399**	11.872	<0.001	0.520	1.924
	GlobalBenefits	0.283	0.038	**0.226**	7.398	<0.001	0.626	1.597
	GlobalRisks	-0.143	0.044	**-0.085**	-3.280	0.001	0.876	1.141
	FriendInjunct	0.113	0.033	**0.097**	3.470	<0.001	0.751	1.332

a. Dependent Variable: Intentions

Linear Regression for Intentions Without Quantitative Risk (Adult Injunctive Norms)

Model Summary

					Change Statistics				
Model	R	R Square	Adjusted R Square	Std. Error of the Estimate	R Square Change	F Change	df1	df2	Sig. F Change
1	0.380[a]	0.144	0.137	0.94441	0.144	20.046	6	713	<0.001
2	0.760[b]	0.578	0.572	0.66544	0.433	181.780	4	709	<0.001
3	0.785[c]	0.617	0.611	0.63427	0.039	72.407	1	708	<0.001

a. Predictors: (Constant), Knowledge, Age, Hispanic, Sex, White, Access

b. Predictors: (Constant), Knowledge, Age, Hispanic, Sex, White, Access, GlobalRisks, GlobalBenefits, StatusQuo, GistPrinciples

c. Predictors: (Constant), Knowledge, Age, Hispanic, Sex, White, Access, GlobalRisks, GlobalBenefits, StatusQuo, GistPrinciples, AdultInjunct

ANOVA[a]

Model		Sum of Squares	df	Mean Square	F	Sig.
1	Regression	107.275	6	17.879	20.046	<0.001[b]
	Residual	635.938	713	0.892		
	Total	743.212	719			
2	Regression	429.256	10	42.926	96.938	<0.001[c]
	Residual	313.957	709	0.443		
	Total	743.212	719			
3	Regression	458.385	11	41.671	103.583	<0.001[d]
	Residual	284.827	708	0.402		
	Total	743.212	719			

a. Dependent Variable: Intentions
b. Predictors: (Constant), Knowledge, Age, Hispanic, Sex, White, Access
c. Predictors: (Constant), Knowledge, Age, Hispanic, Sex, White, Access, GlobalRisks, GlobalBenefits, StatusQuo, GistPrinciples
d. Predictors: (Constant), Knowledge, Age, Hispanic, Sex, White, Access, GlobalRisks, GlobalBenefits, StatusQuo, GistPrinciples, AdultInjunct

Coefficients[a]

Model		Unstandardized Coefficients		Standardized Coefficients			Collinearity Statistics	
		B	Std. Error	Beta	t	Sig.	Tolerance	VIF
1	(Constant)	-0.562	0.588		-0.954	0.340		
	Age	0.035	0.025	0.048	1.386	0.166	0.989	1.011
	Sex	0.113	0.078	0.051	1.444	0.149	0.971	1.029
	White	0.171	0.074	**0.083**	2.329	0.020	0.952	1.050
	Hispanic	0.011	0.114	0.004	0.100	0.920	0.980	1.020
	Access	0.079	0.056	0.060	1.427	0.154	0.683	1.465
	Knowledge	0.781	0.107	**0.306**	7.272	<0.001	0.679	1.473
2	(Constant)	1.608	0.528		3.043	0.002		
	Age	0.033	0.018	0.046	1.864	0.063	0.987	1.013
	Sex	0.082	0.056	0.037	1.470	0.142	0.951	1.051
	White	0.142	0.052	**0.069**	2.730	0.006	0.944	1.060
	Hispanic	0.014	0.081	0.004	0.176	0.860	0.979	1.021
	Access	-0.049	0.040	-0.037	-1.232	0.218	0.671	1.490
	Knowledge	-0.263	0.088	**-0.103**	-3.007	0.003	0.507	1.973

	StatusQuo	-0.343	0.039	**-0.281**	-8.745	<0.001	0.579	1.727
	GistPrinciples	0.668	0.052	**0.423**	12.782	<0.001	0.543	1.840
	GlobalBenefits	0.308	0.038	**0.246**	8.132	<0.001	0.649	1.541
	GlobalRisks	-0.148	0.044	**-0.088**	-3.363	<0.001	0.877	1.140
3	(Constant)	1.159	0.506		2.288	0.022		
	Age	0.047	0.017	**0.064**	2.726	0.007	0.979	1.022
	Sex	0.095	0.053	0.043	1.796	0.073	0.951	1.052
	White	0.119	0.050	**0.058**	2.403	0.017	0.941	1.063
	Hispanic	-0.037	0.077	-0.011	-0.474	0.636	0.973	1.027
	Access	-0.074	0.038	**-0.056**	-1.965	0.050	0.667	1.500
	Knowledge	-0.274	0.083	**-0.107**	-3.282	0.001	0.507	1.974
	StatusQuo	-0.319	0.038	**-0.261**	-8.499	<0.001	0.576	1.737
	GistPrinciples	0.531	0.052	**0.337**	10.146	<0.001	0.492	2.032
	GlobalBenefits	0.241	0.037	**0.193**	6.520	<0.001	0.620	1.614
	GlobalRisks	-0.145	0.042	**-0.086**	-3.464	<0.001	0.877	1.140
	AdultInjunct	0.257	0.030	**0.248**	8.509	<0.001	0.637	1.571

a. Dependent Variable: Intentions

Logistic Regression for Behavior Without Quantitative Risk (Friend Descriptive Norms)

Block 1: Method = Enter

Omnibus Tests of Model Coefficients

		Chi-square	df	Sig.
Step 1	Step	28.835	6	<0.001
	Block	28.835	6	<0.001
	Model	28.835	6	<0.001

Model Summary

Step	-2 Log likelihood	Cox & Snell R Square	Nagelkerke R Square
1	962.480[a]	0.039	0.053

a. Estimation terminated at iteration number 3 because parameter estimates changed by less than 0.001.

Variables in the Equation

		B	S.E.	Wald	df	Sig.	Exp(B)	95% C.I.for EXP(B) Lower	Upper
Step 1[a]	Age	0.022	0.056	0.157	1	0.692	1.022	0.917	1.140
	Sex	-0.081	0.170	0.228	1	0.633	0.922	0.661	1.286
	White	0.267	0.159	2.836	1	0.092	1.307	0.957	1.784
	Hispanic	-0.363	0.247	2.157	1	0.142	0.695	0.428	1.129
	Access	0.072	0.120	0.356	1	0.551	1.074	0.849	1.360
	Knowledge	**0.781**	0.236	11.009	1	<0.001	2.185	1.377	3.466
	Constant	**-3.441**	1.293	7.078	1	0.008	0.032		

a. Variable(s) entered on step 1: Age, Sex, White, Hispanic, Access, Knowledge.

Block 2: Method = Enter

Omnibus Tests of Model Coefficients

		Chi-square	df	Sig.
Step 1	Step	161.578	4	<0.001
	Block	161.578	4	<0.001
	Model	190.413	10	<0.001

Model Summary

Step	-2 Log likelihood	Cox & Snell R Square	Nagelkerke R Square
1	800.902[a]	0.232	0.311

a. Estimation terminated at iteration number 5 because parameter estimates changed by less than 0.001.

Variables in the Equation

		B	S.E.	Wald	df	Sig.	Exp(B)	95% C.I.for EXP(B) Lower	Upper
Step 1[a]	Age	0.010	0.061	0.024	1	0.876	1.010	0.895	1.139
	Sex	-0.138	0.192	0.520	1	0.471	0.871	0.598	1.268
	White	0.267	0.178	2.238	1	0.135	1.306	0.921	1.852
	Hispanic	-0.479	0.274	3.050	1	0.081	0.620	0.362	1.060
	Access	-0.147	0.137	1.144	1	0.285	0.864	0.660	1.130
	Knowledge	**-1.027**	0.309	11.063	1	<0.001	0.358	0.195	0.656
	StatusQuo	**-0.895**	0.146	37.404	1	<0.001	0.409	0.307	0.544

		B	S.E.	Wald	df	Sig.	Exp(B)	Lower	Upper
	GistPrinciples	**1.013**	0.192	27.857	1	<0.001	2.754	1.890	4.012
	GlobalBenefits	**0.382**	0.127	8.975	1	0.003	1.465	1.141	1.880
	GlobalRisks	**-0.371**	0.154	5.809	1	0.016	0.690	0.510	0.933
	Constant	2.462	1.845	1.781	1	0.182	11.728		

a. Variable(s) entered on step 1: StatusQuo, GistPrinciples, GlobalBenefits, GlobalRisks.

Block 3: Method = Enter

Omnibus Tests of Model Coefficients

		Chi-square	df	Sig.
Step 1	Step	35.100	1	<0.001
	Block	35.100	1	<0.001
	Model	225.513	11	<0.001

Model Summary

Step	-2 Log likelihood	Cox & Snell R Square	Nagelkerke R Square
1	765.803[a]	0.269	0.360

a. Estimation terminated at iteration number 5 because parameter estimates changed by less than 0.001.

Variables in the Equation

		B	S.E.	Wald	df	Sig.	Exp(B)	95% C.I.for EXP(B) Lower	Upper
Step 1[a]	Age	0.045	0.063	0.507	1	0.477	1.046	0.924	1.184
	Sex	-0.143	0.197	0.531	1	0.466	0.867	0.590	1.274
	White	0.187	0.183	1.036	1	0.309	1.205	0.841	1.727
	Hispanic	-0.497	0.282	3.094	1	0.079	0.609	0.350	1.058
	Access	-0.187	0.142	1.750	1	0.186	0.829	0.628	1.094
	Knowledge	**-0.990**	0.318	9.688	1	0.002	0.371	0.199	0.693
	StatusQuo	**-0.838**	0.150	31.346	1	<0.001	0.433	0.323	0.580
	GistPrinciples	**0.949**	0.195	23.741	1	<0.001	2.584	1.764	3.785
	GlobalBenefits	**0.368**	0.132	7.714	1	0.005	1.445	1.114	1.873
	GlobalRisks	**-0.427**	0.160	7.127	1	0.008	0.652	0.477	0.893
	FriendDescr	**1.070**	0.183	34.019	1	<0.001	2.915	2.035	4.177
	Constant	1.655	1.898	0.760	1	0.383	5.231		

a. Variable(s) entered on step 1: FriendDescr.

Logistic Regression for Behavior Without Quantitative Risk (Adult Descriptive Norms)

Block 1: Method = Enter

Omnibus Tests of Model Coefficients

		Chi-square	df	Sig.
Step 1	Step	28.835	6	<0.001
	Block	28.835	6	<0.001
	Model	28.835	6	<0.001

Model Summary

Step	-2 Log likelihood	Cox & Snell R Square	Nagelkerke R Square
1	962.480[a]	0.039	0.053

a. Estimation terminated at iteration number 3 because parameter estimates changed by less than 0.001.

Variables in the Equation

		B	S.E.	Wald	df	Sig.	Exp(B)	95% C.I.for EXP(B)	
								Lower	Upper
Step 1[a]	Age	0.022	0.056	0.157	1	0.692	1.022	0.917	1.140
	Sex	-0.081	0.170	0.228	1	0.633	0.922	0.661	1.286
	White	0.267	0.159	2.836	1	0.092	1.307	0.957	1.784
	Hispanic	-0.363	0.247	2.157	1	0.142	0.695	0.428	1.129
	Access	0.072	0.120	0.356	1	0.551	1.074	0.849	1.360
	Knowledge	**0.781**	0.236	11.009	1	<0.001	2.185	1.377	3.466
	Constant	-3.441	1.293	7.078	1	0.008	0.032		

a. Variable(s) entered on step 1: Age, Sex, White, Hispanic, Access, Knowledge.

Block 2: Method = Enter

Omnibus Tests of Model Coefficients

		Chi-square	df	Sig.
Step 1	Step	161.578	4	<0.001
	Block	161.578	4	<0.001
	Model	190.413	10	<0.001

Model Summary

Step	-2 Log likelihood	Cox & Snell R Square	Nagelkerke R Square
1	800.902[a]	0.232	0.311

a. Estimation terminated at iteration number 5 because parameter estimates changed by less than 0.001.

Variables in the Equation

		B	S.E.	Wald	df	Sig.	Exp(B)	95% C.I.for EXP(B) Lower	Upper
Step 1[a]	Age	0.010	0.061	0.024	1	0.876	1.010	0.895	1.139
	Sex	-0.138	0.192	0.520	1	0.471	0.871	0.598	1.268
	White	0.267	0.178	2.238	1	0.135	1.306	0.921	1.852
	Hispanic	-0.479	0.274	3.050	1	0.081	0.620	0.362	1.060
	Access	-0.147	0.137	1.144	1	0.285	0.864	0.660	1.130
	Knowledge	**-1.027**	0.309	11.063	1	<0.001	0.358	0.195	0.656
	StatusQuo	**-0.895**	0.146	37.404	1	<0.001	0.409	0.307	0.544
	GistPrinciples	**1.013**	0.192	27.857	1	<0.001	2.754	1.890	4.012
	GlobalBenefits	**0.382**	0.127	8.975	1	0.003	1.465	1.141	1.880
	GlobalRisks	**-0.371**	0.154	5.809	1	0.016	0.690	0.510	0.933
	Constant	2.462	1.845	1.781	1	0.182	11.728		

a. Variable(s) entered on step 1: StatusQuo, GistPrinciples, GlobalBenefits, GlobalRisks.

Block 3: Method = Enter

Omnibus Tests of Model Coefficients

		Chi-square	df	Sig.
Step 1	Step	31.504	1	<0.001
	Block	31.504	1	<0.001
	Model	221.917	11	<0.001

Model Summary

Step	-2 Log likelihood	Cox & Snell R Square	Nagelkerke R Square
1	769.399[a]	0.265	0.355

a. Estimation terminated at iteration number 5 because parameter estimates changed by less than 0.001.

Variables in the Equation

		B	S.E.	Wald	df	Sig.	Exp(B)	95% C.I.for EXP(B) Lower	Upper
Step 1[a]	Age	0.040	0.063	0.395	1	0.530	1.041	0.919	1.178
	Sex	-0.139	0.195	0.504	1	0.478	0.870	0.593	1.277
	White	0.190	0.183	1.074	1	0.300	1.209	0.845	1.729
	Hispanic	-0.465	0.280	2.761	1	0.097	0.628	0.363	1.087
	Access	-0.144	0.141	1.045	1	0.307	0.866	0.656	1.142
	Knowledge	**-1.005**	0.317	10.041	1	0.002	0.366	0.197	0.682
	StatusQuo	**-0.800**	0.149	28.846	1	<0.001	0.449	0.335	0.602
	GistPrinciples	**0.874**	0.195	20.022	1	<0.001	2.396	1.634	3.513
	GlobalBenefits	**0.344**	0.131	6.906	1	0.009	1.411	1.091	1.824
	GlobalRisks	**-0.376**	0.157	5.721	1	0.017	0.687	0.504	0.934
	AdultDescr	**1.017**	0.182	31.213	1	<0.001	2.765	1.935	3.950
	Constant	1.748	1.892	0.853	1	0.356	5.744		

a. Variable(s) entered on step 1: AdultDescr.

Logistic Regression for Behavior Without Quantitative Risk (Friend Injunctive Norms)

Block 1: Method = Enter

Omnibus Tests of Model Coefficients

		Chi-square	df	Sig.
Step 1	Step	28.835	6	<0.001
	Block	28.835	6	<0.001
	Model	28.835	6	<0.001

Model Summary

Step	-2 Log likelihood	Cox & Snell R Square	Nagelkerke R Square
1	962.480[a]	0.039	0.053

a. Estimation terminated at iteration number 3 because parameter estimates changed by less than 0.001.

Variables in the Equation

		B	S.E.	Wald	df	Sig.	Exp(B)	95% C.I.for EXP(B) Lower	Upper

		B	S.E.	Wald	df	Sig.	Exp(B)	Lower	Upper
Step 1[a]	Age	0.022	0.056	0.157	1	0.692	1.022	0.917	1.140
	Sex	-0.081	0.170	0.228	1	0.633	0.922	0.661	1.286
	White	0.267	0.159	2.836	1	0.092	1.307	0.957	1.784
	Hispanic	-0.363	0.247	2.157	1	0.142	0.695	0.428	1.129
	Access	0.072	0.120	0.356	1	0.551	1.074	0.849	1.360
	Knowledge	**0.781**	0.236	11.009	1	<0.001	2.185	1.377	3.466
	Constant	-3.441	1.293	7.078	1	0.008	0.032		

a. Variable(s) entered on step 1: Age, Sex, White, Hispanic, Access, Knowledge.

Block 2: Method = Enter

Omnibus Tests of Model Coefficients

		Chi-square	df	Sig.
Step 1	Step	161.578	4	<0.001
	Block	161.578	4	<0.001
	Model	190.413	10	<0.001

Model Summary

Step	-2 Log likelihood	Cox & Snell R Square	Nagelkerke R Square
1	800.902[a]	0.232	0.311

a. Estimation terminated at iteration number 5 because parameter estimates changed by less than 0.001.

Variables in the Equation

		B	S.E.	Wald	df	Sig.	Exp(B)	95% C.I.for EXP(B)	
								Lower	Upper
Step 1[a]	Age	0.010	0.061	0.024	1	0.876	1.010	0.895	1.139
	Sex	-0.138	0.192	0.520	1	0.471	0.871	0.598	1.268
	White	0.267	0.178	2.238	1	0.135	1.306	0.921	1.852
	Hispanic	-0.479	0.274	3.050	1	0.081	0.620	0.362	1.060
	Access	-0.147	0.137	1.144	1	0.285	0.864	0.660	1.130
	Knowledge	**-1.027**	0.309	11.063	1	<0.001	0.358	0.195	0.656
	StatusQuo	**-0.895**	0.146	37.404	1	<0.001	0.409	0.307	0.544
	GistPrinciples	**1.013**	0.192	27.857	1	<0.001	2.754	1.890	4.012
	GlobalBenefits	**0.382**	0.127	8.975	1	0.003	1.465	1.141	1.880
	GlobalRisks	**-0.371**	0.154	5.809	1	0.016	0.690	0.510	0.933
	Constant	2.462	1.845	1.781	1	0.182	11.728		

a. Variable(s) entered on step 1: StatusQuo, GistPrinciples, GlobalBenefits, GlobalRisks.

Block 3: Method = Enter

Omnibus Tests of Model Coefficients

		Chi-square	df	Sig.
Step 1	Step	5.826	1	0.016
	Block	5.826	1	0.016
	Model	196.239	11	<0.001

Model Summary

Step	-2 Log likelihood	Cox & Snell R Square	Nagelkerke R Square
1	795.076[a]	0.239	0.319

a. Estimation terminated at iteration number 5 because parameter estimates changed by less than 0.001.

Variables in the Equation

		B	S.E.	Wald	df	Sig.	Exp(B)	95% C.I.for EXP(B) Lower	Upper
Step 1[a]	Age	0.003	0.062	0.003	1	0.958	1.003	0.889	1.132
	Sex	-0.140	0.193	0.527	1	0.468	0.869	0.596	1.269
	White	0.235	0.180	1.712	1	0.191	1.265	0.889	1.800
	Hispanic	-0.499	0.275	3.293	1	0.070	0.607	0.354	1.041
	Access	-0.133	0.138	0.924	1	0.336	0.876	0.668	1.148
	Knowledge	**-1.096**	0.312	12.330	1	<0.001	0.334	0.181	0.616
	StatusQuo	**-0.879**	0.147	35.611	1	<0.001	0.415	0.311	0.554
	GistPrinciples	**0.923**	0.196	22.243	1	<0.001	2.517	1.715	3.694
	GlobalBenefits	**0.324**	0.131	6.137	1	0.013	1.382	1.070	1.786
	GlobalRisks	**-0.360**	0.155	5.392	1	0.020	0.698	0.515	0.945
	FriendInjunct	**0.283**	0.117	5.798	1	0.016	1.327	1.054	1.670
	Constant	2.203	1.853	1.414	1	0.234	9.054		

a. Variable(s) entered on step 1: FriendInjunct.

Logistic Regression for Behavior Without Quantitative Risk (Adult Injunctive Norms)

Block 1: Method = Enter

Omnibus Tests of Model Coefficients

		Chi-square	df	Sig.
Step 1	Step	28.835	6	<0.001
	Block	28.835	6	<0.001
	Model	28.835	6	<0.001

Model Summary

Step	-2 Log likelihood	Cox & Snell R Square	Nagelkerke R Square
1	962.480[a]	0.039	0.053

a. Estimation terminated at iteration number 3 because parameter estimates changed by less than 0.001.

Variables in the Equation

		B	S.E.	Wald	df	Sig.	Exp(B)	95% C.I.for EXP(B) Lower	Upper
Step 1[a]	Age	0.022	0.056	0.157	1	0.692	1.022	0.917	1.140
	Sex	-0.081	0.170	0.228	1	0.633	0.922	0.661	1.286
	White	0.267	0.159	2.836	1	0.092	1.307	0.957	1.784
	Hispanic	-0.363	0.247	2.157	1	0.142	0.695	0.428	1.129
	Access	0.072	0.120	0.356	1	0.551	1.074	0.849	1.360
	Knowledge	**0.781**	0.236	11.009	1	<0.001	2.185	1.377	3.466
	Constant	-3.441	1.293	7.078	1	0.008	0.032		

a. Variable(s) entered on step 1: Age, Sex, White, Hispanic, Access, Knowledge.

Block 2: Method = Enter

Omnibus Tests of Model Coefficients

		Chi-square	df	Sig.
Step 1	Step	161.578	4	<0.001
	Block	161.578	4	<0.001
	Model	190.413	10	<0.001

Model Summary

Step	-2 Log likelihood	Cox & Snell R Square	Nagelkerke R Square
1	800.902[a]	0.232	0.311

a. Estimation terminated at iteration number 5 because parameter estimates changed by less than 0.001.

Variables in the Equation

		B	S.E.	Wald	df	Sig.	Exp(B)	95% C.I.for EXP(B) Lower	Upper
Step 1[a]	Age	0.010	0.061	0.024	1	0.876	1.010	0.895	1.139
	Sex	-0.138	0.192	0.520	1	0.471	0.871	0.598	1.268
	White	0.267	0.178	2.238	1	0.135	1.306	0.921	1.852
	Hispanic	-0.479	0.274	3.050	1	0.081	0.620	0.362	1.060
	Access	-0.147	0.137	1.144	1	0.285	0.864	0.660	1.130
	Knowledge	**-1.027**	0.309	11.063	1	<0.001	0.358	0.195	0.656
	StatusQuo	**-0.895**	0.146	37.404	1	<0.001	0.409	0.307	0.544
	GistPrinciples	**1.013**	0.192	27.857	1	<0.001	2.754	1.890	4.012
	GlobalBenefits	**0.382**	0.127	8.975	1	0.003	1.465	1.141	1.880
	GlobalRisks	**-0.371**	0.154	5.809	1	0.016	0.690	0.510	0.933
	Constant	2.462	1.845	1.781	1	0.182	11.728		

a. Variable(s) entered on step 1: StatusQuo, GistPrinciples, GlobalBenefits, GlobalRisks.

Block 3: Method = Enter

Omnibus Tests of Model Coefficients

		Chi-square	df	Sig.
Step 1	Step	19.398	1	<0.001
	Block	19.398	1	<0.001
	Model	209.811	11	<0.001

Model Summary

Step	-2 Log likelihood	Cox & Snell R Square	Nagelkerke R Square
1	781.505[a]	0.253	0.338

a. Estimation terminated at iteration number 5 because parameter estimates changed by less than 0.001.

Variables in the Equation

		B	S.E.	Wald	df	Sig.	Exp(B)	95% C.I.for EXP(B) Lower	Upper
Step 1[a]	Age	0.032	0.063	0.254	1	0.614	1.032	0.913	1.167
	Sex	-0.109	0.194	0.318	1	0.573	0.896	0.613	1.311
	White	0.242	0.181	1.789	1	0.181	1.274	0.893	1.817
	Hispanic	**-0.602**	0.280	4.638	1	0.031	0.548	0.317	0.947
	Access	-0.202	0.141	2.060	1	0.151	0.817	0.620	1.077
	Knowledge	**-1.084**	0.313	12.000	1	<0.001	0.338	0.183	0.625

	B	S.E.	Wald	df	Sig.	Exp(B)	Lower	Upper
StatusQuo	**-0.876**	0.148	35.204	1	<0.001	0.417	0.312	0.556
GistPrinciples	**0.781**	0.201	15.113	1	<0.001	2.184	1.473	3.239
GlobalBenefits	**0.271**	0.132	4.221	1	0.040	1.311	1.013	1.697
GlobalRisks	**-0.349**	0.156	4.999	1	0.025	0.705	0.520	0.958
AdultInjunct	**0.483**	0.112	18.602	1	<0.001	1.622	1.302	2.020
Constant	1.716	1.874	0.838	1	0.360	5.560		

a. Variable(s) entered on step 1: AdultInjunct.

Linear Regression for Intentions Without Knowledge (Friend Descriptive Norms)

Model Summary

					Change Statistics				
Model	R	R Square	Adjusted R Square	Std. Error of the Estimate	R Square Change	F Change	df1	df2	Sig. F Change
1	0.284[a]	0.081	0.074	0.97813	0.081	12.564	5	714	<0.001
2	0.760[b]	0.578	0.572	0.66486	0.497	167.276	5	709	<0.001
3	0.764[c]	0.583	0.577	0.66133	0.005	8.584	1	708	0.004

a. Predictors: (Constant), Access, Age, Hispanic, Sex, White
b. Predictors: (Constant), Access, Age, Hispanic, Sex, White, GlobalRisks, GlobalBenefits, QuantRisk, StatusQuo, GistPrinciples
c. Predictors: (Constant), Access, Age, Hispanic, Sex, White, GlobalRisks, GlobalBenefits, QuantRisk, StatusQuo, GistPrinciples, FriendDescr

ANOVA[a]

Model		Sum of Squares	df	Mean Square	F	Sig.
1	Regression	60.104	5	12.021	12.564	<0.001[b]
	Residual	683.108	714	0.957		
	Total	743.212	719			
2	Regression	429.811	10	42.981	97.235	<0.001[c]
	Residual	313.401	709	0.442		
	Total	743.212	719			
3	Regression	433.565	11	39.415	90.121	<0.001[d]
	Residual	309.647	708	0.437		
	Total	743.212	719			

a. Dependent Variable: Intentions
b. Predictors: (Constant), Access, Age, Hispanic, Sex, White

c. Predictors: (Constant), Access, Age, Hispanic, Sex, White, GlobalRisks, GlobalBenefits, QuantRisk, StatusQuo, GistPrinciples
d. Predictors: (Constant), Access, Age, Hispanic, Sex, White, GlobalRisks, GlobalBenefits, QuantRisk, StatusQuo, GistPrinciples, FriendDescr

Coefficients[a]

		Unstandardized Coefficients		Standardized Coefficients			Collinearity Statistics	
Model		B	Std. Error	Beta	t	Sig.	Tolerance	VIF
1	(Constant)	1.229	0.554		2.221	0.027		
	Age	0.044	0.026	0.060	1.667	0.096	0.991	1.009
	Sex	0.137	0.081	0.062	1.694	0.091	0.973	1.028
	White	0.236	0.076	**0.114**	3.115	0.002	0.966	1.035
	Hispanic	-0.041	0.118	-0.013	-0.348	0.728	0.984	1.016
	Access	0.294	0.049	**0.221**	6.011	<0.001	0.949	1.054
2	(Constant)	0.695	0.462		1.506	0.132		
	Age	0.030	0.018	0.041	1.679	0.094	0.988	1.012
	Sex	0.071	0.055	0.032	1.281	0.201	0.954	1.049
	White	0.121	0.052	**0.058**	2.329	0.020	0.947	1.056
	Hispanic	0.024	0.080	0.007	0.305	0.761	0.983	1.018
	Access	-0.080	0.037	**-0.060**	-2.175	0.030	0.778	1.285
	StatusQuo	-0.309	0.037	**-0.253**	-8.283	<0.001	0.639	1.565
	GistPrinciples	0.668	0.052	**0.423**	12.802	<0.001	0.545	1.836
	GlobalBenefits	0.308	0.038	**0.247**	8.145	<0.001	0.649	1.540
	GlobalRisks	-0.164	0.045	**-0.097**	-3.643	<0.001	0.833	1.201
	QuantRisk	0.007	0.002	**0.088**	3.212	0.001	0.798	1.253
3	(Constant)	0.610	0.460		1.325	0.185		
	Age	0.035	0.018	0.047	1.931	0.054	0.981	1.020
	Sex	0.070	0.055	0.031	1.265	0.206	0.954	1.049
	White	0.109	0.052	**0.052**	2.099	0.036	0.941	1.063
	Hispanic	0.026	0.080	0.008	0.323	0.747	0.983	1.018
	Access	-0.083	0.036	**-0.063**	-2.284	0.023	0.778	1.286
	StatusQuo	-0.297	0.037	**-0.242**	-7.928	<0.001	0.630	1.586
	GistPrinciples	0.651	0.052	**0.413**	12.485	<0.001	0.538	1.857
	GlobalBenefits	0.305	0.038	**0.244**	8.084	<0.001	0.649	1.542
	GlobalRisks	-0.165	0.045	**-0.098**	-3.676	<0.001	0.833	1.201
	QuantRisk	0.006	0.002	**0.083**	3.070	0.002	0.796	1.256
	FriendDescr	0.153	0.052	**0.075**	2.930	0.004	0.906	1.103

a. Dependent Variable: Intentions

Linear Regression for Intentions Without Knowledge (Adult Descriptive Norms)

Model Summary

					Change Statistics				
Model	R	R Square	Adjusted R Square	Std. Error of the Estimate	R Square Change	F Change	df1	df2	Sig. F Change
1	0.284[a]	0.081	0.074	0.97813	0.081	12.564	5	714	<0.001
2	0.760[b]	0.578	0.572	0.66486	0.497	167.276	5	709	<0.001
3	0.775[c]	0.601	0.595	0.64702	0.023	40.627	1	708	<0.001

a. Predictors: (Constant), Access, Age, Hispanic, Sex, White
b. Predictors: (Constant), Access, Age, Hispanic, Sex, White, GlobalRisks, GlobalBenefits, QuantRisk, StatusQuo, GistPrinciples
c. Predictors: (Constant), Access, Age, Hispanic, Sex, White, GlobalRisks, GlobalBenefits, QuantRisk, StatusQuo, GistPrinciples, AdultDescr

ANOVA[a]

Model		Sum of Squares	df	Mean Square	F	Sig.
1	Regression	60.104	5	12.021	12.564	<0.001[b]
	Residual	683.108	714	0.957		
	Total	743.212	719			
2	Regression	429.811	10	42.981	97.235	<0.001[c]
	Residual	313.401	709	0.442		
	Total	743.212	719			
3	Regression	446.819	11	40.620	97.029	<0.001[d]
	Residual	296.393	708	0.419		
	Total	743.212	719			

a. Dependent Variable: Intentions
b. Predictors: (Constant), Access, Age, Hispanic, Sex, White
c. Predictors: (Constant), Access, Age, Hispanic, Sex, White, GlobalRisks, GlobalBenefits, QuantRisk, StatusQuo, GistPrinciples
d. Predictors: (Constant), Access, Age, Hispanic, Sex, White, GlobalRisks, GlobalBenefits, QuantRisk, StatusQuo, GistPrinciples, AdultDescr

Coefficients[a]

Model		Unstandardized Coefficients B	Std. Error	Standardized Coefficients Beta	t	Sig.	Collinearity Statistics Tolerance	VIF
1	(Constant)	1.229	0.554		2.221	0.027		
	Age	0.044	0.026	0.060	1.667	0.096	0.991	1.009
	Sex	0.137	0.081	0.062	1.694	0.091	0.973	1.028
	White	0.236	0.076	**0.114**	3.115	0.002	0.966	1.035
	Hispanic	-0.041	0.118	-0.013	-0.348	0.728	0.984	1.016
	Access	0.294	0.049	**0.221**	6.011	<0.001	0.949	1.054
2	(Constant)	0.695	0.462		1.506	0.132		
	Age	0.030	0.018	0.041	1.679	0.094	0.988	1.012
	Sex	0.071	0.055	0.032	1.281	0.201	0.954	1.049
	White	0.121	0.052	**0.058**	2.329	0.020	0.947	1.056
	Hispanic	0.024	0.080	0.007	0.305	0.761	0.983	1.018
	Access	-0.080	0.037	**-0.060**	-2.175	0.030	0.778	1.285
	StatusQuo	-0.309	0.037	**-0.253**	-8.283	<0.001	0.639	1.565
	GistPrinciples	0.668	0.052	**0.423**	12.802	<0.001	0.545	1.836
	GlobalBenefits	0.308	0.038	**0.247**	8.145	<0.001	0.649	1.540
	GlobalRisks	-0.164	0.045	**-0.097**	-3.643	<0.001	0.833	1.201
	QuantRisk	0.007	0.002	**0.088**	3.212	0.001	0.798	1.253
3	(Constant)	0.535	0.450		1.190	0.235		
	Age	0.039	0.018	**0.053**	2.209	0.027	0.982	1.018
	Sex	0.069	0.054	0.031	1.276	0.202	0.954	1.049
	White	0.093	0.051	0.045	1.832	0.067	0.940	1.064
	Hispanic	0.035	0.078	0.011	0.446	0.656	0.982	1.018
	Access	-0.081	0.036	**-0.061**	-2.257	0.024	0.778	1.285
	StatusQuo	-0.273	0.037	**-0.223**	-7.432	<0.001	0.624	1.602
	GistPrinciples	0.616	0.051	**0.390**	11.979	<0.001	0.531	1.884
	GlobalBenefits	0.291	0.037	**0.233**	7.872	<0.001	0.646	1.549
	GlobalRisks	-0.153	0.044	**-0.091**	-3.480	<0.001	0.831	1.203
	QuantRisk	0.006	0.002	**0.072**	2.703	0.007	0.792	1.263
	AdultDescr	0.336	0.053	**0.165**	6.374	<0.001	0.837	1.195

a. Dependent Variable: Intentions

Linear Regression for Intentions Without Knowledge (Friend Injunctive Norms)

Model Summary

Model	R	R Square	Change Statistics

			Adjusted R Square	Std. Error of the Estimate	R Square Change	F Change	df1	df2	Sig. F Change
1	0.284[a]	0.081	0.074	0.97813	0.081	12.564	5	714	<0.001
2	0.760[b]	0.578	0.572	0.66486	0.497	167.276	5	709	<0.001
3	0.765[c]	0.585	0.579	0.65968	0.007	12.172	1	708	<0.001

a. Predictors: (Constant), Access, Age, Hispanic, Sex, White
b. Predictors: (Constant), Access, Age, Hispanic, Sex, White, GlobalRisks, GlobalBenefits, QuantRisk, StatusQuo, GistPrinciples
c. Predictors: (Constant), Access, Age, Hispanic, Sex, White, GlobalRisks, GlobalBenefits, QuantRisk, StatusQuo, GistPrinciples, FriendInjunct

ANOVA[a]

Model		Sum of Squares	df	Mean Square	F	Sig.
1	Regression	60.104	5	12.021	12.564	<0.001[b]
	Residual	683.108	714	0.957		
	Total	743.212	719			
2	Regression	429.811	10	42.981	97.235	<0.001[c]
	Residual	313.401	709	0.442		
	Total	743.212	719			
3	Regression	435.108	11	39.555	90.895	<0.001[d]
	Residual	308.104	708	0.435		
	Total	743.212	719			

a. Dependent Variable: Intentions
b. Predictors: (Constant), Access, Age, Hispanic, Sex, White
c. Predictors: (Constant), Access, Age, Hispanic, Sex, White, GlobalRisks, GlobalBenefits, QuantRisk, StatusQuo, GistPrinciples
d. Predictors: (Constant), Access, Age, Hispanic, Sex, White, GlobalRisks, GlobalBenefits, QuantRisk, StatusQuo, GistPrinciples, FriendInjunct

Coefficients[a]

		Unstandardized Coefficients		Standardized Coefficients			Collinearity Statistics	
Model		B	Std. Error	Beta	t	Sig.	Tolerance	VIF
1	(Constant)	1.229	0.554		2.221	0.027		
	Age	0.044	0.026	0.060	1.667	0.096	0.991	1.009
	Sex	0.137	0.081	0.062	1.694	0.091	0.973	1.028
	White	0.236	0.076	**0.114**	3.115	0.002	0.966	1.035

	Hispanic	-0.041	0.118	-0.013	-0.348	0.728	0.984	1.016
	Access	0.294	0.049	**0.221**	6.011	<0.001	0.949	1.054
2	(Constant)	0.695	0.462		1.506	0.132		
	Age	0.030	0.018	0.041	1.679	0.094	0.988	1.012
	Sex	0.071	0.055	0.032	1.281	0.201	0.954	1.049
	White	0.121	0.052	**0.058**	2.329	0.020	0.947	1.056
	Hispanic	0.024	0.080	0.007	0.305	0.761	0.983	1.018
	Access	-0.080	0.037	**-0.060**	-2.175	0.030	0.778	1.285
	StatusQuo	-0.309	0.037	**-0.253**	-8.283	<0.001	0.639	1.565
	GistPrinciples	0.668	0.052	**0.423**	12.802	<0.001	0.545	1.836
	GlobalBenefits	0.308	0.038	**0.247**	8.145	<0.001	0.649	1.540
	GlobalRisks	-0.164	0.045	**-0.097**	-3.643	<0.001	0.833	1.201
	QuantRisk	0.007	0.002	**0.088**	3.212	0.001	0.798	1.253
3	(Constant)	0.505	0.461		1.095	0.274		
	Age	0.027	0.018	0.038	1.543	0.123	0.986	1.014
	Sex	0.069	0.055	0.031	1.250	0.212	0.954	1.049
	White	0.108	0.052	**0.052**	2.086	0.037	0.942	1.062
	Hispanic	0.021	0.080	0.006	0.265	0.791	0.983	1.018
	Access	-0.078	0.036	**-0.059**	-2.136	0.033	0.778	1.285
	StatusQuo	-0.298	0.037	**-0.244**	-8.017	<0.001	0.634	1.576
	GistPrinciples	0.629	0.053	**0.399**	11.884	<0.001	0.521	1.921
	GlobalBenefits	0.283	0.038	**0.226**	7.408	<0.001	0.626	1.596
	GlobalRisks	-0.160	0.045	**-0.095**	-3.590	<0.001	0.832	1.201
	QuantRisk	0.007	0.002	**0.094**	3.468	<0.001	0.795	1.258
	FriendInjunct	0.114	0.033	**0.097**	3.489	<0.001	0.751	1.332

a. Dependent Variable: Intentions

Linear Regression for Intentions Without Knowledge (Adult Injunctive Norms)

Model Summary

					Change Statistics				
Model	R	R Square	Adjusted R Square	Std. Error of the Estimate	R Square Change	F Change	df1	df2	Sig. F Change
1	0.284[a]	0.081	0.074	0.97813	0.081	12.564	5	714	<0.001
2	0.760[b]	0.578	0.572	0.66486	0.497	167.276	5	709	<0.001
3	0.787[c]	0.619	0.613	0.63230	0.041	75.880	1	708	<0.001

a. Predictors: (Constant), Access, Age, Hispanic, Sex, White

b. Predictors: (Constant), Access, Age, Hispanic, Sex, White, GlobalRisks, GlobalBenefits, QuantRisk, StatusQuo, GistPrinciples
c. Predictors: (Constant), Access, Age, Hispanic, Sex, White, GlobalRisks, GlobalBenefits, QuantRisk, StatusQuo, GistPrinciples, AdultInjunct

ANOVA[a]

Model		Sum of Squares	df	Mean Square	F	Sig.
1	Regression	60.104	5	12.021	12.564	<0.001[b]
	Residual	683.108	714	0.957		
	Total	743.212	719			
2	Regression	429.811	10	42.981	97.235	<0.001[c]
	Residual	313.401	709	0.442		
	Total	743.212	719			
3	Regression	460.148	11	41.832	104.629	<0.001[d]
	Residual	283.064	708	0.400		
	Total	743.212	719			

a. Dependent Variable: Intentions
b. Predictors: (Constant), Access, Age, Hispanic, Sex, White
c. Predictors: (Constant), Access, Age, Hispanic, Sex, White, GlobalRisks, GlobalBenefits, QuantRisk, StatusQuo, GistPrinciples
d. Predictors: (Constant), Access, Age, Hispanic, Sex, White, GlobalRisks, GlobalBenefits, QuantRisk, StatusQuo, GistPrinciples, AdultInjunct

Coefficients[a]

		Unstandardized Coefficients		Standardized Coefficients			Collinearity Statistics	
Model		B	Std. Error	Beta	t	Sig.	Tolerance	VIF
1	(Constant)	1.229	0.554		2.221	0.027		
	Age	0.044	0.026	0.060	1.667	0.096	0.991	1.009
	Sex	0.137	0.081	0.062	1.694	0.091	0.973	1.028
	White	0.236	0.076	**0.114**	3.115	0.002	0.966	1.035
	Hispanic	-0.041	0.118	-0.013	-0.348	0.728	0.984	1.016
	Access	0.294	0.049	**0.221**	6.011	<0.001	0.949	1.054
2	(Constant)	0.695	0.462		1.506	0.132		
	Age	0.030	0.018	0.041	1.679	0.094	0.988	1.012
	Sex	0.071	0.055	0.032	1.281	0.201	0.954	1.049
	White	0.121	0.052	**0.058**	2.329	0.020	0.947	1.056

	Hispanic	0.024	0.080	0.007	0.305	0.761	0.983	1.018
	Access	-0.080	0.037	**-0.060**	-2.175	0.030	0.778	1.285
	StatusQuo	-0.309	0.037	**-0.253**	-8.283	<0.001	0.639	1.565
	GistPrinciples	0.668	0.052	**0.423**	12.802	<0.001	0.545	1.836
	GlobalBenefits	0.308	0.038	**0.247**	8.145	<0.001	0.649	1.540
	GlobalRisks	-0.164	0.045	**-0.097**	-3.643	<0.001	0.833	1.201
	QuantRisk	0.007	0.002	**0.088**	3.212	0.001	0.798	1.253
3	(Constant)	0.183	0.443		0.414	0.679		
	Age	0.044	0.017	**0.060**	2.540	0.011	0.980	1.021
	Sex	0.084	0.053	0.038	1.595	0.111	0.953	1.049
	White	0.096	0.049	0.046	1.936	0.053	0.944	1.060
	Hispanic	-0.028	0.077	-0.008	-0.359	0.720	0.977	1.024
	Access	-0.105	0.035	**-0.079**	-3.010	0.003	0.773	1.294
	StatusQuo	-0.283	0.036	**-0.231**	-7.948	<0.001	0.635	1.576
	GistPrinciples	0.530	0.052	**0.336**	10.179	<0.001	0.494	2.023
	GlobalBenefits	0.241	0.037	**0.192**	6.535	<0.001	0.620	1.612
	GlobalRisks	-0.167	0.043	**-0.099**	-3.901	<0.001	0.833	1.201
	QuantRisk	0.008	0.002	**0.102**	3.905	<0.001	0.795	1.257
	AdultInjunct	0.262	0.030	**0.254**	8.711	<0.001	0.634	1.577

a. Dependent Variable: Intentions

Logistic Regression for Behavior Without Knowledge (Friend Descriptive Norms)

Block 1: Method = Enter

Omnibus Tests of Model Coefficients

		Chi-square	df	Sig.
Step 1	Step	17.616	5	0.003
	Block	17.616	5	0.003
	Model	17.616	5	0.003

Model Summary

Step	-2 Log likelihood	Cox & Snell R Square	Nagelkerke R Square
1	973.700[a]	0.024	0.032

a. Estimation terminated at iteration number 3 because parameter estimates changed by less than 0.001.

Variables in the Equation

		B	S.E.	Wald	df	Sig.	Exp(B)	95% C.I.for EXP(B)	
								Lower	Upper
Step 1[a]	Age	0.030	0.055	0.299	1	0.585	1.031	0.925	1.148
	Sex	-0.054	0.168	0.103	1	0.748	0.947	0.682	1.317
	White	**0.326**	0.157	4.336	1	0.037	1.386	1.019	1.884
	Hispanic	-0.410	0.245	2.795	1	0.095	0.664	0.410	1.073
	Access	**0.282**	0.102	7.627	1	0.006	1.326	1.085	1.620
	Constant	-1.624	1.157	1.969	1	0.161	0.197		

a. Variable(s) entered on step 1: Age, Sex, White, Hispanic, Access.

Block 2: Method = Enter

Omnibus Tests of Model Coefficients

		Chi-square	df	Sig.
Step 1	Step	172.139	5	<0.001
	Block	172.139	5	<0.001
	Model	189.754	10	<0.001

Model Summary

Step	-2 Log likelihood	Cox & Snell R Square	Nagelkerke R Square
1	801.561[a]	0.232	0.310

a. Estimation terminated at iteration number 4 because parameter estimates changed by less than 0.001.

Variables in the Equation

		B	S.E.	Wald	df	Sig.	Exp(B)	95% C.I.for EXP(B)	
								Lower	Upper
Step 1[a]	Age	0.000	0.062	0.000	1	0.995	1.000	0.886	1.129
	Sex	-0.165	0.192	0.741	1	0.389	0.848	0.582	1.235
	White	0.186	0.178	1.095	1	0.295	1.204	0.850	1.706
	Hispanic	-0.430	0.272	2.505	1	0.114	0.651	0.382	1.108
	Access	**-0.257**	0.129	3.963	1	0.047	0.773	0.600	0.996
	StatusQuo	**-0.745**	0.135	30.612	1	<0.001	0.475	0.365	0.618
	GistPrinciples	**0.996**	0.191	27.317	1	<0.001	2.708	1.864	3.934
	GlobalBenefits	**0.374**	0.128	8.512	1	0.004	1.453	1.130	1.867

		B	S.E.	Wald	df	Sig.	Exp(B)	Lower	Upper
	GlobalRisks	**-0.414**	0.159	6.820	1	0.009	0.661	0.484	0.902
	QuantRisk	**0.024**	0.007	10.380	1	0.001	1.024	1.009	1.039
	Constant	-1.180	1.594	0.548	1	0.459	0.307		

a. Variable(s) entered on step 1: StatusQuo, GistPrinciples, GlobalBenefits, GlobalRisks, QuantRisk.

Block 3: Method = Enter

Omnibus Tests of Model Coefficients

		Chi-square	df	Sig.
Step 1	Step	34.548	1	<0.001
	Block	34.548	1	<0.001
	Model	224.302	11	<0.001

Model Summary

Step	-2 Log likelihood	Cox & Snell R Square	Nagelkerke R Square
1	767.013[a]	0.268	0.358

a. Estimation terminated at iteration number 5 because parameter estimates changed by less than 0.001.

Variables in the Equation

		B	S.E.	Wald	df	Sig.	Exp(B)	95% C.I.for EXP(B) Lower	Upper
Step 1[a]	Age	0.036	0.063	0.330	1	0.566	1.037	0.916	1.174
	Sex	-0.160	0.197	0.666	1	0.415	0.852	0.579	1.252
	White	0.111	0.183	0.367	1	0.545	1.117	0.781	1.599
	Hispanic	-0.444	0.280	2.519	1	0.113	0.642	0.371	1.110
	Access	**-0.296**	0.133	4.922	1	0.027	0.744	0.573	0.966
	StatusQuo	**-0.686**	0.137	25.016	1	<0.001	0.504	0.385	0.659
	GistPrinciples	**0.932**	0.194	23.057	1	<0.001	2.540	1.736	3.716
	GlobalBenefits	**0.356**	0.132	7.247	1	0.007	1.428	1.102	1.850
	GlobalRisks	**-0.461**	0.164	7.883	1	0.005	0.631	0.457	0.870
	QuantRisk	**0.022**	0.008	8.499	1	0.004	1.022	1.007	1.038
	FriendDescr	**1.062**	0.183	33.574	1	<0.001	2.893	2.020	4.144
	Constant	-1.865	1.633	1.304	1	0.253	0.155		

a. Variable(s) entered on step 1: FriendDescr.

Logistic Regression for Behavior Without Knowledge (Adult Descriptive Norms)

Block 1: Method = Enter

Omnibus Tests of Model Coefficients

		Chi-square	df	Sig.
Step 1	Step	17.616	5	0.003
	Block	17.616	5	0.003
	Model	17.616	5	0.003

Model Summary

Step	-2 Log likelihood	Cox & Snell R Square	Nagelkerke R Square
1	973.700[a]	0.024	0.032

a. Estimation terminated at iteration number 3 because parameter estimates changed by less than 0.001.

Variables in the Equation

		B	S.E.	Wald	df	Sig.	Exp(B)	95% C.I.for EXP(B)	
								Lower	Upper
Step 1[a]	Age	0.030	0.055	0.299	1	0.585	1.031	0.925	1.148
	Sex	-0.054	0.168	0.103	1	0.748	0.947	0.682	1.317
	White	0.326	0.157	4.336	1	0.037	1.386	1.019	1.884
	Hispanic	-0.410	0.245	2.795	1	0.095	0.664	0.410	1.073
	Access	0.282	0.102	7.627	1	0.006	1.326	1.085	1.620
	Constant	-1.624	1.157	1.969	1	0.161	0.197		

a. Variable(s) entered on step 1: Age, Sex, White, Hispanic, Access.

Block 2: Method = Enter

Omnibus Tests of Model Coefficients

		Chi-square	df	Sig.
Step 1	Step	172.139	5	<0.001
	Block	172.139	5	<0.001
	Model	189.754	10	<0.001

Model Summary

Step	-2 Log likelihood	Cox & Snell R Square	Nagelkerke R Square
1	801.561[a]	0.232	0.310

a. Estimation terminated at iteration number 4 because parameter estimates changed by less than 0.001.

Variables in the Equation

		B	S.E.	Wald	df	Sig.	Exp(B)	95% C.I.for EXP(B) Lower	Upper
Step 1[a]	Age	0.000	0.062	0.000	1	0.995	1.000	0.886	1.129
	Sex	-0.165	0.192	0.741	1	0.389	0.848	0.582	1.235
	White	0.186	0.178	1.095	1	0.295	1.204	0.850	1.706
	Hispanic	-0.430	0.272	2.505	1	0.114	0.651	0.382	1.108
	Access	-0.257	0.129	3.963	1	0.047	0.773	0.600	0.996
	StatusQuo	-0.745	0.135	30.612	1	<0.001	0.475	0.365	0.618
	GistPrinciples	0.996	0.191	27.317	1	<0.001	2.708	1.864	3.934
	GlobalBenefits	0.374	0.128	8.512	1	0.004	1.453	1.130	1.867
	GlobalRisks	-0.414	0.159	6.820	1	0.009	0.661	0.484	0.902
	QuantRisk	0.024	0.007	10.380	1	0.001	1.024	1.009	1.039
	Constant	-1.180	1.594	0.548	1	0.459	0.307		

a. Variable(s) entered on step 1: StatusQuo, GistPrinciples, GlobalBenefits, GlobalRisks, QuantRisk.

Block 3: Method = Enter

Omnibus Tests of Model Coefficients

		Chi-square	df	Sig.
Step 1	Step	29.731	1	<0.001
	Block	29.731	1	<0.001
	Model	219.485	11	<0.001

Model Summary

Step	-2 Log likelihood	Cox & Snell R Square	Nagelkerke R Square
1	771.830[a]	0.263	0.351

a. Estimation terminated at iteration number 5 because parameter estimates changed by less than 0.001.

Variables in the Equation

		B	S.E.	Wald	df	Sig.	Exp(B)	95% C.I.for EXP(B) Lower	Upper
	Age	0.031	0.063	0.238	1	0.626	1.031	0.911	1.168

		B	S.E.	Wald	df	Sig.	Exp(B)	Lower	Upper
Step 1[a]	Sex	-0.160	0.195	0.667	1	0.414	0.852	0.581	1.250
	White	0.112	0.182	0.380	1	0.538	1.119	0.783	1.599
	Hispanic	-0.412	0.277	2.207	1	0.137	0.662	0.385	1.141
	Access	**-0.261**	0.133	3.867	1	0.049	0.770	0.594	0.999
	StatusQuo	**-0.653**	0.137	22.533	1	<0.001	0.521	0.398	0.682
	GistPrinciples	**0.857**	0.194	19.455	1	<0.001	2.356	1.610	3.449
	GlobalBenefits	**0.332**	0.131	6.382	1	0.012	1.393	1.077	1.802
	GlobalRisks	**-0.395**	0.161	6.002	1	0.014	0.674	0.491	0.924
	QuantRisk	**0.021**	0.007	7.702	1	0.006	1.021	1.006	1.036
	AdultDescr	**0.990**	0.182	29.560	1	<0.001	2.692	1.884	3.846
	Constant	-1.771	1.629	1.181	1	0.277	0.170		

a. Variable(s) entered on step 1: AdultDescr.

Logistic Regression for Behavior Without Knowledge (Friend Injunctive Norms)

Block 1: Method = Enter

Omnibus Tests of Model Coefficients

		Chi-square	df	Sig.
Step 1	Step	17.616	5	0.003
	Block	17.616	5	0.003
	Model	17.616	5	0.003

Model Summary

Step	-2 Log likelihood	Cox & Snell R Square	Nagelkerke R Square
1	973.700[a]	0.024	0.032

a. Estimation terminated at iteration number 3 because parameter estimates changed by less than 0.001.

Variables in the Equation

		B	S.E.	Wald	df	Sig.	Exp(B)	95% C.I.for EXP(B)	
								Lower	Upper
Step 1[a]	Age	0.030	0.055	0.299	1	0.585	1.031	0.925	1.148
	Sex	-0.054	0.168	0.103	1	0.748	0.947	0.682	1.317
	White	**0.326**	0.157	4.336	1	0.037	1.386	1.019	1.884
	Hispanic	-0.410	0.245	2.795	1	0.095	0.664	0.410	1.073

		B	S.E.	Wald	df	Sig.	Exp(B)	Lower	Upper
	Access	**0.282**	0.102	7.627	1	0.006	1.326	1.085	1.620
	Constant	-1.624	1.157	1.969	1	0.161	0.197		

a. Variable(s) entered on step 1: Age, Sex, White, Hispanic, Access.

Block 2: Method = Enter

Omnibus Tests of Model Coefficients

		Chi-square	df	Sig.
Step 1	Step	172.139	5	<0.001
	Block	172.139	5	<0.001
	Model	189.754	10	<0.001

Model Summary

Step	-2 Log likelihood	Cox & Snell R Square	Nagelkerke R Square
1	801.561[a]	0.232	0.310

a. Estimation terminated at iteration number 4 because parameter estimates changed by less than 0.001.

Variables in the Equation

		B	S.E.	Wald	df	Sig.	Exp(B)	95% C.I.for EXP(B) Lower	Upper
Step 1[a]	Age	0.000	0.062	0.000	1	0.995	1.000	0.886	1.129
	Sex	-0.165	0.192	0.741	1	0.389	0.848	0.582	1.235
	White	0.186	0.178	1.095	1	0.295	1.204	0.850	1.706
	Hispanic	-0.430	0.272	2.505	1	0.114	0.651	0.382	1.108
	Access	**-0.257**	0.129	3.963	1	0.047	0.773	0.600	0.996
	StatusQuo	**-0.745**	0.135	30.612	1	<0.001	0.475	0.365	0.618
	GistPrinciples	**0.996**	0.191	27.317	1	<0.001	2.708	1.864	3.934
	GlobalBenefits	**0.374**	0.128	8.512	1	0.004	1.453	1.130	1.867
	GlobalRisks	**-0.414**	0.159	6.820	1	0.009	0.661	0.484	0.902
	QuantRisk	**0.024**	0.007	10.380	1	0.001	1.024	1.009	1.039
	Constant	-1.180	1.594	0.548	1	0.459	0.307		

a. Variable(s) entered on step 1: StatusQuo, GistPrinciples, GlobalBenefits, GlobalRisks, QuantRisk.

Block 3: Method = Enter

Omnibus Tests of Model Coefficients

		Chi-square	df	Sig.
Step 1	Step	5.618	1	0.018
	Block	5.618	1	0.018
	Model	195.372	11	<0.001

Model Summary

Step	-2 Log likelihood	Cox & Snell R Square	Nagelkerke R Square
1	795.943[a]	0.238	0.318

a. Estimation terminated at iteration number 4 because parameter estimates changed by less than 0.001.

Variables in the Equation

		B	S.E.	Wald	df	Sig.	Exp(B)	95% C.I.for EXP(B)	
								Lower	Upper
Step 1[a]	Age	-0.006	0.062	0.010	1	0.922	0.994	0.880	1.122
	Sex	-0.167	0.193	0.750	1	0.387	0.846	0.580	1.235
	White	0.148	0.180	0.675	1	0.411	1.159	0.815	1.648
	Hispanic	-0.439	0.272	2.601	1	0.107	0.645	0.378	1.099
	Access	-0.251	0.130	3.746	1	0.053	0.778	0.603	1.003
	StatusQuo	**-0.721**	0.136	28.225	1	<0.001	0.486	0.373	0.634
	GistPrinciples	**0.906**	0.194	21.719	1	<0.001	2.474	1.690	3.620
	GlobalBenefits	**0.312**	0.131	5.667	1	0.017	1.367	1.057	1.767
	GlobalRisks	**-0.409**	0.160	6.543	1	0.011	0.664	0.485	0.909
	QuantRisk	**0.025**	0.007	11.498	1	<0.001	1.025	1.011	1.040
	FriendInjunct	**0.276**	0.116	5.610	1	0.018	1.318	1.049	1.655
	Constant	-1.641	1.613	1.034	1	0.309	0.194		

a. Variable(s) entered on step 1: FriendInjunct.

Logistic Regression for Behavior Without Knowledge (Adult Injunctive Norms)

Block 1: Method = Enter

Omnibus Tests of Model Coefficients

		Chi-square	df	Sig.
Step 1	Step	17.616	5	0.003
	Block	17.616	5	0.003
	Model	17.616	5	0.003

Model Summary

Step	-2 Log likelihood	Cox & Snell R Square	Nagelkerke R Square
1	973.700[a]	0.024	0.032

a. Estimation terminated at iteration number 3 because parameter estimates changed by less than 0.001.

Variables in the Equation

		B	S.E.	Wald	df	Sig.	Exp(B)	95% C.I.for EXP(B)	
								Lower	Upper
Step 1[a]	Age	0.030	0.055	0.299	1	0.585	1.031	0.925	1.148
	Sex	-0.054	0.168	0.103	1	0.748	0.947	0.682	1.317
	White	**0.326**	0.157	4.336	1	0.037	1.386	1.019	1.884
	Hispanic	-0.410	0.245	2.795	1	0.095	0.664	0.410	1.073
	Access	**0.282**	0.102	7.627	1	0.006	1.326	1.085	1.620
	Constant	-1.624	1.157	1.969	1	0.161	0.197		

a. Variable(s) entered on step 1: Age, Sex, White, Hispanic, Access.

Block 2: Method = Enter

Omnibus Tests of Model Coefficients

		Chi-square	df	Sig.
Step 1	Step	172.139	5	<0.001
	Block	172.139	5	<0.001
	Model	189.754	10	<0.001

Model Summary

Step	-2 Log likelihood	Cox & Snell R Square	Nagelkerke R Square
1	801.561[a]	0.232	0.310

a. Estimation terminated at iteration number 4 because parameter estimates changed by less than 0.001.

Variables in the Equation

	B	S.E.	Wald	df	Sig.	Exp(B)	95% C.I.for EXP(B)	
							Lower	Upper
Age	0.000	0.062	0.000	1	0.995	1.000	0.886	1.129

		B	S.E.	Wald	df	Sig.	Exp(B)	Lower	Upper
Step 1[a]	Sex	-0.165	0.192	0.741	1	0.389	0.848	0.582	1.235
	White	0.186	0.178	1.095	1	0.295	1.204	0.850	1.706
	Hispanic	-0.430	0.272	2.505	1	0.114	0.651	0.382	1.108
	Access	**-0.257**	0.129	3.963	1	0.047	0.773	0.600	0.996
	StatusQuo	**-0.745**	0.135	30.612	1	<0.001	0.475	0.365	0.618
	GistPrinciples	**0.996**	0.191	27.317	1	<0.001	2.708	1.864	3.934
	GlobalBenefits	**0.374**	0.128	8.512	1	0.004	1.453	1.130	1.867
	GlobalRisks	**-0.414**	0.159	6.820	1	0.009	0.661	0.484	0.902
	QuantRisk	**0.024**	0.007	10.380	1	0.001	1.024	1.009	1.039
	Constant	-1.180	1.594	0.548	1	0.459	0.307		

a. Variable(s) entered on step 1: StatusQuo, GistPrinciples, GlobalBenefits, GlobalRisks, QuantRisk.

Block 3: Method = Enter

Omnibus Tests of Model Coefficients

		Chi-square	df	Sig.
Step 1	Step	21.327	1	<0.001
	Block	21.327	1	<0.001
	Model	211.081	11	<0.001

Model Summary

Step	-2 Log likelihood	Cox & Snell R Square	Nagelkerke R Square
1	780.234[a]	0.254	0.340

a. Estimation terminated at iteration number 5 because parameter estimates changed by less than 0.001.

Variables in the Equation

								95% C.I.for EXP(B)	
		B	S.E.	Wald	df	Sig.	Exp(B)	Lower	Upper
Step 1[a]	Age	0.023	0.063	0.137	1	0.712	1.023	0.905	1.157
	Sex	-0.134	0.194	0.478	1	0.489	0.874	0.598	1.279
	White	0.145	0.181	0.645	1	0.422	1.156	0.811	1.649
	Hispanic	**-0.555**	0.277	4.014	1	0.045	0.574	0.333	0.988
	Access	**-0.317**	0.132	5.741	1	0.017	0.728	0.562	0.944
	StatusQuo	**-0.721**	0.136	28.183	1	<0.001	0.486	0.373	0.635
	GistPrinciples	**0.759**	0.200	14.474	1	<0.001	2.136	1.445	3.159

GlobalBenefits	0.255	0.132	3.736	1	0.053	1.291	0.996	1.672
GlobalRisks	**-0.425**	0.162	6.873	1	0.009	0.654	0.476	0.898
QuantRisk	**0.027**	0.008	13.175	1	<0.001	1.028	1.013	1.043
AdultInjunct	**0.507**	0.112	20.473	1	<0.001	1.660	1.333	2.068
Constant	-2.172	1.631	1.774	1	0.183	0.114		

a. Variable(s) entered on step 1: AdultInjunct.

Community Sample

Bivariate Correlations

	Age (N = 160)	Sex(N = 160)	Race(N=160)	Ethnicity (N = 160)	Access (N = 198)	Knowledge (N = 236)	Knowledge Correct (N = 236)	Status_Quo (N = 217)
Age (N = 160)	1	0.016	0.102	-0.026	0.128	**0.156**[*]	0.138	-0.130
Sex[a] (N = 160)	0.016	1	-0.018	0.078	0.113	0.084	0.114	-0.048
Race[b] (N = 160)	0.102	-0.018	1	-0.114	-0.031	0.138	0.132	**-0.169**[*]
Ethnicity[c] (N = 160)	-0.026	0.078	-0.114	1	0.056	0.038	0.027	-0.026
Access (N = 198)	0.128	0.113	-0.031	0.056	1	**0.264**[**]	**0.229**[**]	-0.117
Knowledge Rating (N = 236)	**0.156**[*]	0.084	0.138	0.038	**0.264**[**]	1	**0.965**[**]	**-0.728**[**]
KnowledgeCorrect (N = 236)	0.138	0.114	0.132	0.027	**0.229**[**]	**0.965**[**]	1	**-0.701**[**]
Status_Quo (N = 217)	-0.130	-0.048	**-0.169**[*]	-0.026	-0.117	**-0.728**[**]	**-0.701**[**]	1
GistPrinciples (N = 217)	0.126	0.062	0.039	0.074	**0.142**[*]	**0.790**[**]	**0.779**[**]	**-0.724**[**]
GlobalBenefits (N = 185)	0.113	-0.006	0.004	0.143	**0.145**[*]	**0.681**[**]	**0.676**[**]	**-0.671**[**]
GlobalRisks (N = 185)	-0.127	-0.092	**-0.230**[**]	-0.017	-0.081	**-0.528**[**]	**-0.536**[**]	**0.340**[**]
QuantRisk (N = 189)	-0.114	0.025	0.052	-0.077	-0.115	**-0.590**[**]	**-0.621**[**]	**0.384**[**]
SNS (N = 173)	0.087	**-0.210**[**]	0.135	-0.017	**0.158**[*]	0.034	-0.014	0.043
CRT (N = 166)	**-0.210**[**]	**-0.178**[*]	0.100	-0.087	0.026	-0.002	0.012	0.070
Intentions (N = 198)	**0.180**[*]	0.071	0.106	0.048	**0.189**[**]	**0.739**[**]	**0.729**[**]	**-0.753**[**]
Behavior (N = 236)	**0.211**[**]	0.056	0.088	0.076	**0.291**[**]	**0.479**[**]	**0.472**[**]	**-0.492**[**]

	GistPrinciples (N = 217)	GlobalBenefits (N = 185)	GlobalRisks (N = 185)	QuantRisk (N = 189)	SNS (N = 173)	CRT (N = 166)	Intentions (N = 198)	Behavior (N = 236)
Age (N = 160)	0.126	0.113	-0.127	-0.114	0.087	**-0.210**[**]	**0.180**[*]	**0.211**[**]

	GistPrinciples (N = 217)	GlobalBenefits (N = 185)	GlobalRisks (N = 185)	QuantRisk (N = 189)	SNS (N = 173)	CRT (N = 166)	Intentions (N = 198)	Behavior (N = 236)
Sex[a] (N = 160)	0.062	-0.006	-0.092	0.025	**-0.210****	**-0.178***	0.071	0.056
Race[b] (N = 160)	0.039	0.004	**-0.230****	0.052	0.135	0.100	0.106	0.088
Ethnicity[c] (N = 160)	0.074	0.143	-0.017	-0.077	-0.017	-0.087	0.048	0.076
Access (N = 198)	**0.142***	**0.145***	-0.081	-0.115	**0.158***	0.026	**0.189****	**0.291****
Knowledge Rating (N = 236)	**0.790****	**0.681****	**-0.528****	**-0.590****	0.034	-0.002	**0.739****	**0.479****
KnowledgeCorrect (N = 236)	**0.779****	**0.676****	**-0.536****	**-0.621****	-0.014	0.012	**0.729****	**0.472****
Status_Quo (N = 217)	**-0.724****	**-0.671****	**0.340****	**0.384****	0.043	0.070	**-0.753****	**-0.492****
GistPrinciples (N = 217)	1	**0.812****	**-0.449****	**-0.464****	-0.085	-0.089	**0.836****	**0.588****
GlobalBenefits (N = 185)	**0.812****	1	**-0.423****	**-0.446****	-0.114	**-0.169***	**0.819****	**0.589****
GlobalRisks (N = 185)	**-0.449****	**-0.423****	1	**0.598****	0.003	0.019	**-0.450****	**-0.272****
QuantRisk (N = 189)	**-0.464****	**-0.446****	**0.598****	1	0.005	-0.120	**-0.416****	**-0.280****
SNS (N = 173)	-0.085	-0.114	0.003	0.005	1	**0.374****	-0.037	-0.047
CRT (N = 166)	-0.089	**-0.169***	0.019	-0.120	**0.374****	1	-0.116	-0.104
Intentions (N = 198)	**0.836****	**0.819****	**-0.450****	**-0.416****	-0.037	-0.116	1	**0.729****
Behavior (N = 236)	**0.588****	**0.589****	**-0.272****	**-0.280****	-0.047	-0.104	**0.729****	1

**p < .01, *p < .05

[a]0 = male, 1 = female. [b]0 = Non-white, 1 = while. [c]0 = Not Hispanic, 1 = Hispanic

Linear Regression for Intentions with Demographics – Including Quantitative Risk and SNS

Model Summary

					Change Statistics				
Model	R	R Square	Adjusted R Square	Std. Error of the Estimate	R Square Change	F Change	df1	df2	Sig. F Change
1	0.758	0.574	0.558	0.95016	0.574	34.410	6	153	<0.001
2	0.894	0.800	0.785	0.66229	0.226	33.382	5	148	<0.001
3	0.895	0.800	0.784	0.66372	0.000	0.366	1	147	0.546

Coefficients[a]

		Unstandardized Coefficients		Standardized Coefficients			Collinearity Statistics	
Model		B	Std. Error	Beta	t	Sig.	Tolerance	VIF
1	(Constant)	-5.019	0.717		-6.997	<0.001		
	age	0.007	0.006	0.066	1.219	0.225	0.959	1.043
	Sex	0.020	0.167	0.006	0.117	0.907	0.979	1.022
	White	-0.005	0.199	-0.001	-0.025	0.980	0.956	1.046
	Hispanic	0.101	0.253	0.021	0.398	0.691	0.977	1.023
	Access	-0.012	0.109	-0.006	-0.111	0.912	0.918	1.089
	Knowledge	2.049	0.153	**0.745**	13.419	<0.001	0.903	1.108
2	(Constant)	-0.232	0.952		-0.243	0.808		
	age	0.005	0.004	0.049	1.289	0.199	0.955	1.048
	Sex	0.067	0.119	0.021	0.565	0.573	0.937	1.067
	White	0.071	0.153	0.019	0.463	0.644	0.790	1.266
	Hispanic	-0.153	0.179	-0.032	-0.855	0.394	0.947	1.055
	Access	0.123	0.078	0.063	1.588	0.114	0.872	1.146
	Knowledge	0.116	0.221	0.042	0.526	0.600	0.210	4.760
	Status_Quo	-0.273	0.090	**-0.191**	-3.039	0.003	0.343	2.913
	GistPrinciples	0.474	0.111	**0.363**	4.258	<0.001	0.186	5.383
	GlobalBenefits	0.472	0.089	**0.343**	5.324	<0.001	0.325	3.075
	GlobalRisks	-0.170	0.087	-0.101	-1.952	0.053	0.508	1.968
	QuantRisk	0.006	0.004	0.071	1.316	0.190	0.464	2.156
3	(Constant)	-0.383	0.986		-0.389	0.698		
	age	0.005	0.004	0.047	1.239	0.217	0.950	1.053
	Sex	0.086	0.123	0.027	0.697	0.487	0.878	1.138
	White	0.064	0.154	0.017	0.413	0.680	0.785	1.274

Hispanic	-0.159	0.180	-0.033	-0.881	0.380	0.945	1.058
Access	0.119	0.078	0.060	1.516	0.132	0.864	1.158
Knowledge	0.098	0.223	0.036	0.439	0.661	0.206	4.847
Status_Quo	-0.274	0.090	**-0.191**	-3.039	0.003	0.343	2.913
GistPrinciples	0.478	0.112	**0.366**	4.277	<0.001	0.185	5.402
GlobalBenefits	0.480	0.090	**0.349**	5.343	<0.001	0.318	3.149
GlobalRisks	-0.166	0.087	-0.099	-1.906	0.059	0.506	1.975
QuantRisk	0.006	0.004	0.070	1.294	0.198	0.463	2.158
SNS	0.043	0.072	0.024	0.605	0.546	0.866	1.154

a. Dependent Variable: Intentions

Logistic Regression for Behavior with Demographics – Including Quantitative Risk and SNS
Block 1

Omnibus Tests of Model Coefficients

		Chi-square	df	Sig.
Step 1	Step	59.393	6	<0.001
	Block	59.393	6	<0.001
	Model	59.393	6	<0.001

Model Summary

Step	-2 Log likelihood	Cox & Snell R Square	Nagelkerke R Square
1	158.800[a]	0.310	0.417

a. Estimation terminated at iteration number 5 because parameter estimates changed by less than 0.001.

Variables in the Equation

								95% C.I.for EXP(B)	
		B	S.E.	Wald	df	Sig.	Exp(B)	Lower	Upper
Step 1[a]	age	0.026	0.015	2.819	1	0.093	1.026	0.996	1.057
	Sex	-0.004	0.439	0.000	1	0.993	0.996	0.421	2.353
	White	-0.025	0.513	0.002	1	0.962	0.976	0.357	2.669
	Hispanic	0.593	0.698	0.724	1	0.395	1.810	0.461	7.103
	Access	**0.651**	0.284	5.271	1	0.022	1.918	1.100	3.345
	Knowledge	**2.811**	0.598	22.124	1	<0.001	16.624	5.153	53.630

Constant	-15.644	2.820	30.772	1	<0.001	0.000

a. Variable(s) entered on step 1: age, Sex, White, Hispanic, Access, Knowledge.

Block 2

Omnibus Tests of Model Coefficients

		Chi-square	df	Sig.
Step 1	Step	31.403	5	<0.001
	Block	31.403	5	<0.001
	Model	90.796	11	<0.001

Model Summary

Step	-2 Log likelihood	Cox & Snell R Square	Nagelkerke R Square
1	127.397[a]	0.433	0.582

a. Estimation terminated at iteration number 6 because parameter estimates changed by less than 0.001.

Variables in the Equation

								95% C.I.for EXP(B)	
		B	S.E.	Wald	df	Sig.	Exp(B)	Lower	Upper
Step 1[a]	age	0.026	0.017	2.223	1	0.136	1.026	0.992	1.061
	Sex	0.032	0.515	0.004	1	0.951	1.032	0.376	2.833
	White	0.308	0.604	0.261	1	0.609	1.361	0.417	4.444
	Hispanic	-0.070	0.819	0.007	1	0.932	0.933	0.187	4.642
	Access	**0.865**	0.330	6.846	1	0.009	2.374	1.242	4.538
	Knowledge	-0.358	0.952	0.142	1	0.707	0.699	0.108	4.516
	Status_Quo	-0.077	0.376	0.041	1	0.839	0.926	0.443	1.935
	GistPrinciples	**1.294**	0.518	6.245	1	0.012	3.647	1.322	10.059
	GlobalBenefits	**0.841**	0.356	5.590	1	0.018	2.318	1.155	4.652
	GlobalRisks	-0.314	0.365	0.738	1	0.390	0.731	0.357	1.494
	QuantRisk	0.021	0.023	0.835	1	0.361	1.021	0.976	1.069
	Constant	-11.129	4.251	6.853	1	0.009	0.000		

a. Variable(s) entered on step 1: Status_Quo, GistPrinciples, GlobalBenefits, GlobalRisks, QuantRisk.

Block 3

Omnibus Tests of Model Coefficients

		Chi-square	df	Sig.
Step 1	Step	0.924	1	0.336
	Block	0.924	1	0.336
	Model	91.720	12	<0.001

Model Summary

Step	-2 Log likelihood	Cox & Snell R Square	Nagelkerke R Square
1	126.473[a]	0.436	0.586

a. Estimation terminated at iteration number 6 because parameter estimates changed by less than 0.001.

Variables in the Equation

		B	S.E.	Wald	df	Sig.	Exp(B)	95% C.I.for EXP(B) Lower	95% C.I.for EXP(B) Upper
Step 1[a]	age	0.026	0.017	2.297	1	0.130	1.027	0.992	1.062
	Sex	-0.115	0.531	0.047	1	0.828	0.891	0.315	2.521
	White	0.399	0.606	0.434	1	0.510	1.491	0.455	4.886
	Hispanic	-0.049	0.830	0.003	1	0.953	0.953	0.187	4.847
	Access	**0.920**	0.340	7.334	1	0.007	2.508	1.289	4.879
	Knowledge	-0.205	0.971	0.044	1	0.833	0.815	0.121	5.470
	Status_Quo	-0.109	0.377	0.083	1	0.774	0.897	0.428	1.880
	GistPrinciples	**1.274**	0.516	6.081	1	0.014	3.574	1.299	9.835
	GlobalBenefits	**0.760**	0.360	4.461	1	0.035	2.139	1.056	4.330
	GlobalRisks	-0.350	0.366	0.911	1	0.340	0.705	0.344	1.445
	QuantRisk	0.021	0.023	0.804	1	0.370	1.021	0.976	1.069
	SNS	-0.334	0.351	0.907	1	0.341	0.716	0.360	1.424
	Constant	-9.984	4.356	5.252	1	0.022	0.000		

a. Variable(s) entered on step 1: SNS.

Linear Regression for Intentions WITHOUT Demographics – Including Quantitative risk and SNS

Model Summary

Model	R	R Square	Adjusted R Square	Std. Error of the Estimate	Change Statistics: R Square Change	F Change	df1	df2	Sig. F Change

1	0.741[a]	0.550	0.545	0.96102	0.550	103.810	2	170	<0.001
2	0.889[b]	0.791	0.782	0.66439	0.241	38.138	5	165	<0.001
3	0.890[c]	0.792	0.782	0.66456	0.001	0.915	1	164	0.340

a. Predictors: (Constant), Knowledge, Access
b. Predictors: (Constant), Knowledge, Access, GlobalRisks, QuantRisk, GlobalBenefits, Status_Quo, GistPrinciples
c. Predictors: (Constant), Knowledge, Access, GlobalRisks, QuantRisk, GlobalBenefits, Status_Quo, GistPrinciples, SNS

ANOVA[a]

Model		Sum of Squares	df	Mean Square	F	Sig.
1	Regression	191.749	2	95.874	103.810	<0.001[b]
	Residual	157.004	170	0.924		
	Total	348.753	172			
2	Regression	275.921	7	39.417	89.299	<0.001[c]
	Residual	72.832	165	0.441		
	Total	348.753	172			
3	Regression	276.325	8	34.541	78.211	<0.001[d]
	Residual	72.428	164	0.442		
	Total	348.753	172			

a. Dependent Variable: Intentions
b. Predictors: (Constant), Knowledge, Access
c. Predictors: (Constant), Knowledge, Access, GlobalRisks, QuantRisk, GlobalBenefits, Status_Quo, GistPrinciples
d. Predictors: (Constant), Knowledge, Access, GlobalRisks, QuantRisk, GlobalBenefits, Status_Quo, GistPrinciples, SNS

Coefficients[a]

		Unstandardized Coefficients		Standardized Coefficients			Collinearity Statistics	
Model		B	Std. Error	Beta	t	Sig.	Tolerance	VIF
1	(Constant)	-4.586	0.678		-6.769	<0.001		
	Access	-0.015	0.105	-0.007	-0.139	0.889	0.939	1.065
	Knowledge	2.018	0.144	**0.743**	13.995	<0.001	0.939	1.065
2	(Constant)	0.148	0.887		0.167	0.868		
	Access	0.118	0.074	0.060	1.593	0.113	0.893	1.119
	Knowledge	0.070	0.206	0.026	0.337	0.737	0.219	4.570
	Status_Quo	-0.262	0.085	**-0.182**	-3.086	0.002	0.364	2.749

	GistPrinciples	0.498	0.105	**0.385**	4.720	<0.001	0.191	5.244
	GlobalBenefits	0.478	0.085	**0.350**	5.651	<0.001	0.330	3.034
	GlobalRisks	-0.175	0.078	**-0.106**	-2.257	0.025	0.577	1.734
	QuantRisk	0.006	0.004	0.079	1.598	0.112	0.522	1.914
3	(Constant)	-0.036	0.907		-0.039	0.969		
	Access	0.109	0.075	0.055	1.453	0.148	0.878	1.139
	Knowledge	0.043	0.208	0.016	0.207	0.836	0.215	4.651
	Status_Quo	-0.262	0.085	**-0.182**	-3.090	0.002	0.364	2.749
	GistPrinciples	0.506	0.106	**0.391**	4.781	<0.001	0.189	5.278
	GlobalBenefits	0.487	0.085	**0.356**	5.716	<0.001	0.326	3.064
	GlobalRisks	-0.175	0.078	**-0.106**	-2.252	0.026	0.577	1.734
	QuantRisk	0.006	0.004	0.078	1.582	0.116	0.522	1.914
	SNS	0.060	0.063	0.035	0.956	0.340	0.935	1.070

a. Dependent Variable: Intentions

Logistic Regression for Behavior WITHOUT Demographics – Including Quantitative risk and SNS Block 1

Omnibus Tests of Model Coefficients

		Chi-square	df	Sig.
Step 1	Step	50.074	2	<0.001
	Block	50.074	2	<0.001
	Model	50.074	2	<0.001

Model Summary

Step	-2 Log likelihood	Cox & Snell R Square	Nagelkerke R Square
1	187.199[a]	0.251	0.337

a. Estimation terminated at iteration number 5 because parameter estimates changed by less than 0.001.

Variables in the Equation

							95% C.I.for EXP(B)	
		B	S.E.	Wald	df	Sig.	Exp(B)	Lower	Upper
Step 1[a]	Access	**0.661**	0.264	6.263	1	0.012	1.936	1.154	3.248

		B	S.E.	Wald	df	Sig.	Exp(B)	Lower	Upper
	Knowledge	**2.327**	0.481	23.365	1	<0.001	10.244	3.988	26.315
	Constant	-12.605	2.317	29.605	1	<0.001	0.000		

a. Variable(s) entered on step 1: Access, Knowledge.

Block 2

Omnibus Tests of Model Coefficients

		Chi-square	df	Sig.
Step 1	Step	38.882	5	<0.001
	Block	38.882	5	<0.001
	Model	88.956	7	<0.001

Model Summary

Step	-2 Log likelihood	Cox & Snell R Square	Nagelkerke R Square
1	148.317[a]	0.402	0.539

a. Estimation terminated at iteration number 6 because parameter estimates changed by less than 0.001.

Variables in the Equation

		B	S.E.	Wald	df	Sig.	Exp(B)	95% C.I.for EXP(B)	
								Lower	Upper
Step 1[a]	Access	**0.904**	0.306	8.738	1	0.003	2.469	1.356	4.495
	Knowledge	-1.085	0.869	1.560	1	0.212	0.338	0.062	1.855
	Status_Quo	-0.190	0.346	0.302	1	0.583	0.827	0.420	1.628
	GistPrinciples	**1.310**	0.475	7.598	1	0.006	3.708	1.460	9.413
	GlobalBenefits	**0.773**	0.327	5.578	1	0.018	2.166	1.140	4.113
	GlobalRisks	-0.180	0.319	0.318	1	0.573	0.835	0.447	1.561
	QuantRisk	0.002	0.021	0.010	1	0.922	1.002	0.962	1.043
	Constant	-6.532	3.618	3.261	1	0.071	0.001		

a. Variable(s) entered on step 1: Status_Quo, GistPrinciples, GlobalBenefits, GlobalRisks, QuantRisk.

Block 3

Omnibus Tests of Model Coefficients

		Chi-square	df	Sig.

Step 1	Step	0.111	1	0.739
	Block	0.111	1	0.739
	Model	89.067	8	<0.001

Model Summary

Step	-2 Log likelihood	Cox & Snell R Square	Nagelkerke R Square
1	148.207[a]	0.402	0.539

a. Estimation terminated at iteration number 6 because parameter estimates changed by less than 0.001.

Variables in the Equation

		B	S.E.	Wald	df	Sig.	Exp(B)	95% C.I.for EXP(B) Lower	Upper
Step 1[a]	Access	**0.918**	0.310	8.769	1	0.003	2.504	1.364	4.597
	Knowledge	-1.041	0.879	1.401	1	0.237	0.353	0.063	1.979
	Status_Quo	-0.196	0.346	0.321	1	0.571	0.822	0.418	1.619
	GistPrinciples	**1.300**	0.476	7.474	1	0.006	3.670	1.445	9.321
	GlobalBenefits	**0.755**	0.331	5.201	1	0.023	2.127	1.112	4.070
	GlobalRisks	-0.184	0.319	0.333	1	0.564	0.832	0.445	1.554
	QuantRisk	0.002	0.021	0.008	1	0.928	1.002	0.962	1.043
	SNS	-0.089	0.270	0.109	1	0.741	0.915	0.539	1.552
	Constant	-6.246	3.709	2.836	1	0.092	0.002		

a. Variable(s) entered on step 1: SNS.

Linear Regression for Intentions (Only FTT Predictors)

Model Summary

Model	R	R Square	Adjusted R Square	Std. Error of the Estimate	Change Statistics R Square Change	F Change	df1	df2	Sig. F Change
1	0.887[a]	0.786	0.780	0.66384	0.786	131.590	5	179	<0.001

a. Predictors: (Constant), QuantRisk, Status_Quo, GlobalRisks, GlobalBenefits, GistPrinciples

ANOVA[a]

Model		Sum of Squares	df	Mean Square	F	Sig.
1	Regression	289.952	5	57.990	131.590	<0.001[b]
	Residual	78.883	179	0.441		
	Total	368.836	184			

a. Dependent Variable: Intentions
b. Predictors: (Constant), QuantRisk, Status_Quo, GlobalRisks, GlobalBenefits, GistPrinciples

Coefficients[a]

		Unstandardized Coefficients		Standardized Coefficients			Collinearity Statistics	
Model		B	Std. Error	Beta	t	Sig.	Tolerance	VIF
1	(Constant)	1.029	0.466		2.208	0.029		
	Status_Quo	-0.292	0.079	**-0.203**	-3.693	<0.001	0.395	2.531
	GistPrinciples	0.487	0.092	**0.378**	5.319	<0.001	0.236	4.232
	GlobalBenefits	0.496	0.083	**0.362**	6.006	<0.001	0.329	3.041
	GlobalRisks	-0.156	0.075	**-0.092**	-2.064	0.040	0.601	1.664
	QuantRisk	0.005	0.004	0.057	1.256	0.211	0.589	1.699

a. Dependent Variable: Intentions

Logistic Regression for Behavior (Only FTT Predictors)

Block 1: Method = Enter

Omnibus Tests of Model Coefficients

		Chi-square	df	Sig.
Step 1	Step	86.196	5	<0.001
	Block	86.196	5	<0.001
	Model	86.196	5	<0.001

Model Summary

Step	-2 Log likelihood	Cox & Snell R Square	Nagelkerke R Square
1	165.049[a]	0.372	0.501

a. Estimation terminated at iteration number 6 because parameter estimates changed by less than 0.001.

Variables in the Equation

		B	S.E.	Wald	df	Sig.	Exp(B)	95% C.I.for EXP(B) Lower	Upper
Step 1[a]	Status_Quo	-0.087	0.323	0.073	1	0.787	0.917	0.487	1.725
	GistPrinciples	**1.103**	0.394	7.844	1	0.005	3.012	1.392	6.516
	GlobalBenefits	**0.825**	0.305	7.293	1	0.007	2.281	1.254	4.151
	GlobalRisks	-0.051	0.303	0.028	1	0.867	0.950	0.525	1.722
	QuantRisk	0.003	0.019	0.031	1	0.860	1.003	0.967	1.040
	Constant	-6.661	2.233	8.896	1	0.003	0.001		

a. Variable(s) entered on step 1: Status_Quo, GistPrinciples, GlobalBenefits, GlobalRisks, QuantRisk.

Linear Regression for Intentions Without Quantitative Risk – Including Demographics and SNS

Model Summary

Model	R	R Square	Adjusted R Square	Std. Error of the Estimate	Change Statistics: R Square Change	F Change	df1	df2	Sig. F Change
1	0.758[a]	0.574	0.558	0.95016	0.574	34.410	6	153	<0.001
2	0.893[b]	0.798	0.784	0.66392	0.223	41.092	4	149	<0.001
3	0.893[c]	0.798	0.783	0.66522	0.001	0.416	1	148	0.520

a. Predictors: (Constant), Knowledge, Hispanic, Sex, age, White, Access
b. Predictors: (Constant), Knowledge, Hispanic, Sex, age, White, Access, GlobalRisks, GlobalBenefits, Status_Quo, GistPrinciples
c. Predictors: (Constant), Knowledge, Hispanic, Sex, age, White, Access, GlobalRisks, GlobalBenefits, Status_Quo, GistPrinciples, SNS

ANOVA[a]

Model		Sum of Squares	df	Mean Square	F	Sig.
1	Regression	186.391	6	31.065	34.410	<0.001[b]
	Residual	138.129	153	0.903		
	Total	324.519	159			
2	Regression	258.842	10	25.884	58.722	<0.001[c]
	Residual	65.678	149	0.441		
	Total	324.519	159			

3	Regression	259.026	11	23.548	53.212	<0.001[d]
	Residual	65.494	148	0.443		
	Total	324.519	159			

a. Dependent Variable: Intentions
b. Predictors: (Constant), Knowledge, Hispanic, Sex, age, White, Access
c. Predictors: (Constant), Knowledge, Hispanic, Sex, age, White, Access, GlobalRisks, GlobalBenefits, Status_Quo, GistPrinciples
d. Predictors: (Constant), Knowledge, Hispanic, Sex, age, White, Access, GlobalRisks, GlobalBenefits, Status_Quo, GistPrinciples, SNS

Coefficients[a]

		Unstandardized Coefficients		Standardized Coefficients			Collinearity Statistics	
Model		B	Std. Error	Beta	t	Sig.	Tolerance	VIF
1	(Constant)	-5.019	0.717		-6.997	<0.001		
	age	0.007	0.006	0.066	1.219	0.225	0.959	1.043
	Sex	0.020	0.167	0.006	0.117	0.907	0.979	1.022
	White	-0.005	0.199	-0.001	-0.025	0.980	0.956	1.046
	Hispanic	0.101	0.253	0.021	0.398	0.691	0.977	1.023
	Access	-0.012	0.109	-0.006	-0.111	0.912	0.918	1.089
	Knowledge	2.049	0.153	**0.745**	13.419	<0.001	0.903	1.108
2	(Constant)	0.074	0.925		0.080	0.936		
	age	0.005	0.004	0.047	1.240	0.217	0.956	1.046
	Sex	0.090	0.118	0.029	0.764	0.446	0.958	1.044
	White	0.130	0.147	0.035	0.886	0.377	0.864	1.157
	Hispanic	-0.169	0.179	-0.036	-0.940	0.349	0.951	1.051
	Access	0.130	0.078	0.066	1.667	0.098	0.876	1.142
	Knowledge	0.010	0.206	0.004	0.047	0.962	0.243	4.123
	Status_Quo	-0.274	0.090	**-0.191**	-3.038	0.003	0.343	2.913
	GistPrinciples	0.492	0.111	**0.377**	4.435	<0.001	0.188	5.307
	GlobalBenefits	0.469	0.089	**0.342**	5.286	<0.001	0.325	3.074
	GlobalRisks	-0.115	0.077	-0.068	-1.502	0.135	0.658	1.519
3	(Constant)	-0.092	0.963		-0.096	0.924		
	age	0.005	0.004	0.045	1.188	0.237	0.951	1.052
	Sex	0.110	0.122	0.035	0.899	0.370	0.899	1.113
	White	0.121	0.147	0.033	0.822	0.413	0.857	1.167
	Hispanic	-0.174	0.180	-0.037	-0.966	0.336	0.949	1.053
	Access	0.124	0.078	0.063	1.589	0.114	0.866	1.154
	Knowledge	-0.008	0.208	-0.003	-0.038	0.970	0.238	4.195

Status_Quo	-0.274	0.090	**-0.192**	-3.039	0.003	0.343	2.913
GistPrinciples	0.496	0.111	**0.380**	4.456	<0.001	0.188	5.323
GlobalBenefits	0.478	0.090	**0.348**	5.312	<0.001	0.318	3.148
GlobalRisks	-0.112	0.077	-0.067	-1.463	0.146	0.656	1.523
SNS	0.046	0.072	0.026	0.645	0.520	0.867	1.153

a. Dependent Variable: Intentions

Logistic Regression for Behavior without Quantitative Risk – Including Demographics and SNS

Block 1: Method = Enter

Omnibus Tests of Model Coefficients

		Chi-square	df	Sig.
Step 1	Step	59.393	6	<0.001
	Block	59.393	6	<0.001
	Model	59.393	6	<0.001

Model Summary

Step	-2 Log likelihood	Cox & Snell R Square	Nagelkerke R Square
1	158.800[a]	0.310	0.417

a. Estimation terminated at iteration number 5 because parameter estimates changed by less than 0.001.

Classification Table[a]

			Predicted		
			Behavior		Percentage
	Observed		0.00	1.00	Correct
Step 1	Behavior	0.00	41	27	60.3
		1.00	13	79	85.9
	Overall Percentage				75.0

a. The cut value is 0.500

Variables in the Equation

								95% C.I.for EXP(B)	
		B	S.E.	Wald	df	Sig.	Exp(B)	Lower	Upper
Step 1[a]	age	0.026	0.015	2.819	1	0.093	1.026	0.996	1.057
	Sex	-0.004	0.439	0.000	1	0.993	0.996	0.421	2.353
	White	-0.025	0.513	0.002	1	0.962	0.976	0.357	2.669
	Hispanic	0.593	0.698	0.724	1	0.395	1.810	0.461	7.103

	B	S.E.	Wald	df	Sig.	Exp(B)	Lower	Upper
Access	**0.651**	0.284	5.271	1	0.022	1.918	1.100	3.345
Knowledge	**2.811**	0.598	22.124	1	<0.001	16.624	5.153	53.630
Constant	-15.644	2.820	30.772	1	<0.001	0.000		

a. Variable(s) entered on step 1: age, Sex, White, Hispanic, Access, Knowledge.

Block 2: Method = Enter

Omnibus Tests of Model Coefficients

		Chi-square	df	Sig.
Step 1	Step	30.601	4	<0.001
	Block	30.601	4	<0.001
	Model	89.994	10	<0.001

Model Summary

Step	-2 Log likelihood	Cox & Snell R Square	Nagelkerke R Square
1	128.200[a]	0.430	0.578

a. Estimation terminated at iteration number 6 because parameter estimates changed by less than 0.001.

Classification Table[a]

			Predicted		
			Behavior		Percentage
	Observed		0.00	1.00	Correct
Step 1	Behavior	0.00	49	19	72.1
		1.00	8	84	91.3
	Overall Percentage				83.1

a. The cut value is 0.500

Variables in the Equation

		B	S.E.	Wald	df	Sig.	Exp(B)	95% C.I.for EXP(B) Lower	Upper
Step 1[a]	age	0.026	0.017	2.245	1	0.134	1.026	0.992	1.062
	Sex	0.100	0.506	0.039	1	0.844	1.105	0.409	2.981
	White	0.420	0.594	0.501	1	0.479	1.522	0.475	4.875
	Hispanic	-0.065	0.804	0.007	1	0.936	0.937	0.194	4.528
	Access	**0.887**	0.332	7.124	1	0.008	2.427	1.266	4.654
	Knowledge	-0.495	0.952	0.271	1	0.603	0.609	0.094	3.938
	Status_Quo	-0.099	0.376	0.069	1	0.793	0.906	0.433	1.893
	GistPrinciples	**1.253**	0.509	6.060	1	0.014	3.500	1.291	9.489
	GlobalBenefits	**0.796**	0.353	5.100	1	0.024	2.217	1.111	4.426

		B	S.E.	Wald	df	Sig.	Exp(B)	Lower	Upper
	GlobalRisks	-0.184	0.333	0.305	1	0.581	0.832	0.434	1.597
	Constant	-10.336	4.157	6.181	1	0.013	0.000		

a. Variable(s) entered on step 1: Status_Quo, GistPrinciples, GlobalBenefits, GlobalRisks.

Block 3: Method = Enter

Omnibus Tests of Model Coefficients

		Chi-square	df	Sig.
Step 1	Step	0.951	1	0.329
	Block	0.951	1	0.329
	Model	90.945	11	<0.001

Model Summary

Step	-2 Log likelihood	Cox & Snell R Square	Nagelkerke R Square
1	127.248[a]	0.434	0.583

a. Estimation terminated at iteration number 6 because parameter estimates changed by less than 0.001.

Variables in the Equation

		B	S.E.	Wald	df	Sig.	Exp(B)	95% C.I.for EXP(B) Lower	Upper
Step 1[a]	age	0.027	0.017	2.342	1	0.126	1.027	0.993	1.063
	Sex	-0.051	0.523	0.009	1	0.923	0.950	0.341	2.650
	White	0.502	0.597	0.707	1	0.400	1.651	0.513	5.317
	Hispanic	-0.054	0.817	0.004	1	0.947	0.947	0.191	4.695
	Access	**0.944**	0.342	7.632	1	0.006	2.569	1.315	5.018
	Knowledge	-0.334	0.975	0.118	1	0.732	0.716	0.106	4.839
	Status_Quo	-0.136	0.378	0.130	1	0.719	0.873	0.416	1.831
	GistPrinciples	**1.228**	0.507	5.862	1	0.015	3.414	1.264	9.226
	GlobalBenefits	**0.717**	0.357	4.030	1	0.045	2.048	1.017	4.125
	GlobalRisks	-0.221	0.334	0.437	1	0.509	0.802	0.417	1.543
	SNS	-0.339	0.351	0.932	1	0.334	0.713	0.358	1.418
	Constant	-9.184	4.273	4.620	1	0.032	0.000		

a. Variable(s) entered on step 1: SNS.

Linear Regression for Intentions Without Knowledge—Including Quantitative Risk, Demographics, and SNS

Model Summary

Model	R	R Square	Adjusted R Square	Std. Error of the Estimate	Change Statistics R Square Change	F Change	df1	df2	Sig. F Change
1	0.271[a]	0.073	0.043	1.39737	0.073	2.439	5	154	0.037
2	0.894[b]	0.800	0.786	0.66068	0.726	107.980	5	149	<0.001
3	0.895[c]	0.800	0.785	0.66190	0.001	0.451	1	148	0.503

a. Predictors: (Constant), Access, White, Sex, Hispanic, age
b. Predictors: (Constant), Access, White, Sex, Hispanic, age, GistPrinciples, QuantRisk, GlobalRisks, Status_Quo, GlobalBenefits
c. Predictors: (Constant), Access, White, Sex, Hispanic, age, GistPrinciples, QuantRisk, GlobalRisks, Status_Quo, GlobalBenefits, SNS

ANOVA[a]

Model		Sum of Squares	df	Mean Square	F	Sig.
1	Regression	23.813	5	4.763	2.439	0.037[b]
	Residual	300.706	154	1.953		
	Total	324.519	159			
2	Regression	259.480	10	25.948	59.445	<0.001[c]
	Residual	65.039	149	0.437		
	Total	324.519	159			
3	Regression	259.678	11	23.607	53.883	<0.001[d]
	Residual	64.841	148	0.438		
	Total	324.519	159			

a. Dependent Variable: Intentions
b. Predictors: (Constant), Access, White, Sex, Hispanic, age
c. Predictors: (Constant), Access, White, Sex, Hispanic, age, GistPrinciples, QuantRisk, GlobalRisks, Status_Quo, GlobalBenefits
d. Predictors: (Constant), Access, White, Sex, Hispanic, age, GistPrinciples, QuantRisk, GlobalRisks, Status_Quo, GlobalBenefits, SNS

Coefficients[a]

Model		Unstandardized Coefficients B	Std. Error	Standardized Coefficients Beta	t	Sig.	Collinearity Statistics Tolerance	VIF
1	(Constant)	1.281	0.798		1.606	0.110		
	age	0.016	0.008	0.150	1.903	0.059	0.972	1.029
	Sex	0.150	0.245	0.048	0.611	0.542	0.982	1.018

	White	0.377	0.290	0.102	1.300	0.195	0.976	1.025
	Hispanic	0.241	0.372	0.051	0.647	0.518	0.979	1.022
	Access	0.318	0.156	**0.161**	2.040	0.043	0.967	1.034
2	(Constant)	0.126	0.664		0.190	0.849		
	age	0.005	0.004	0.049	1.296	0.197	0.955	1.047
	Sex	0.071	0.118	0.023	0.604	0.547	0.941	1.062
	White	0.084	0.150	0.023	0.559	0.577	0.812	1.231
	Hispanic	-0.158	0.179	-0.033	-0.886	0.377	0.950	1.053
	Access	0.135	0.074	0.068	1.810	0.072	0.946	1.057
	Status_Quo	-0.287	0.086	**-0.201**	-3.358	<0.001	0.377	2.652
	GistPrinciples	0.501	0.099	**0.384**	5.083	<0.001	0.236	4.240
	GlobalBenefits	0.472	0.088	**0.343**	5.337	<0.001	0.325	3.075
	GlobalRisks	-0.172	0.087	**-0.102**	-1.991	0.048	0.510	1.961
	QuantRisk	0.005	0.004	0.061	1.211	0.228	0.536	1.867
3	(Constant)	-0.101	0.746		-0.136	0.892		
	age	0.005	0.004	0.047	1.241	0.217	0.950	1.053
	Sex	0.091	0.122	0.029	0.746	0.457	0.887	1.127
	White	0.074	0.151	0.020	0.488	0.627	0.804	1.244
	Hispanic	-0.163	0.179	-0.034	-0.910	0.364	0.949	1.054
	Access	0.128	0.075	0.065	1.694	0.092	0.927	1.079
	Status_Quo	-0.285	0.086	**-0.199**	-3.330	0.001	0.377	2.654
	GistPrinciples	0.501	0.099	**0.384**	5.071	<0.001	0.236	4.240
	GlobalBenefits	0.481	0.090	**0.350**	5.368	<0.001	0.318	3.147
	GlobalRisks	-0.168	0.087	-0.100	-1.936	0.055	0.507	1.970
	QuantRisk	0.005	0.004	0.061	1.221	0.224	0.535	1.868
	SNS	0.048	0.071	0.026	0.672	0.503	0.882	1.134

a. Dependent Variable: Intentions

Logistic Regression for Behavior Without Knowledge—Including Quantitative Risk, Demographics, and SNS

Block 1: Method = Enter

Omnibus Tests of Model Coefficients

		Chi-square	df	Sig.
Step 1	Step	22.282	5	<0.001
	Block	22.282	5	<0.001
	Model	22.282	5	<0.001

Model Summary

Step	-2 Log likelihood	Cox & Snell R Square	Nagelkerke R Square
1	195.912[a]	0.130	0.175

a. Estimation terminated at iteration number 4 because parameter estimates changed by less than 0.001.

Variables in the Equation

		B	S.E.	Wald	df	Sig.	Exp(B)	95% C.I.for EXP(B) Lower	Upper
Step 1[a]	age	**0.031**	0.014	4.856	1	0.028	1.031	1.003	1.060
	Sex	0.090	0.380	0.056	1	0.812	1.094	0.520	2.304
	White	0.490	0.443	1.228	1	0.268	1.633	0.686	3.888
	Hispanic	0.589	0.624	0.892	1	0.345	1.803	0.531	6.124
	Access	**0.883**	0.280	9.986	1	0.002	2.419	1.399	4.184
	Constant	-5.517	1.479	13.914	1	<0.001	0.004		

a. Variable(s) entered on step 1: age, Sex, White, Hispanic, Access.

Block 2: Method = Enter

Omnibus Tests of Model Coefficients

		Chi-square	df	Sig.
Step 1	Step	68.373	5	<0.001
	Block	68.373	5	<0.001
	Model	90.655	10	<0.001

Model Summary

Step	-2 Log likelihood	Cox & Snell R Square	Nagelkerke R Square
1	127.538[a]	0.433	0.581

a. Estimation terminated at iteration number 6 because parameter estimates changed by less than 0.001.

Variables in the Equation

		B	S.E.	Wald	df	Sig.	Exp(B)	95% C.I.for EXP(B) Lower	Upper
Step 1[a]	age	0.025	0.017	2.198	1	0.138	1.026	0.992	1.061
	Sex	0.040	0.514	0.006	1	0.938	1.041	0.380	2.850
	White	0.259	0.589	0.193	1	0.660	1.295	0.409	4.106

		B	S.E.	Wald	df	Sig.	Exp(B)	Lower	Upper
	Hispanic	-0.061	0.817	0.006	1	0.941	0.941	0.190	4.668
	Access	**0.831**	0.316	6.923	1	0.009	2.296	1.236	4.265
	Status_Quo	-0.050	0.368	0.019	1	0.892	0.951	0.462	1.957
	GistPrinciples	**1.208**	0.457	6.976	1	0.008	3.346	1.365	8.199
	GlobalBenefits	**0.840**	0.355	5.580	1	0.018	2.315	1.154	4.647
	GlobalRisks	-0.298	0.364	0.670	1	0.413	0.743	0.364	1.515
	QuantRisk	0.023	0.023	0.981	1	0.322	1.023	0.978	1.071
	Constant	-12.202	3.206	14.485	1	<0.001	0.000		

a. Variable(s) entered on step 1: Status_Quo, GistPrinciples, GlobalBenefits, GlobalRisks, QuantRisk.

Block 3: Method = Enter

Omnibus Tests of Model Coefficients

		Chi-square	df	Sig.
Step 1	Step	1.021	1	0.312
	Block	1.021	1	0.312
	Model	91.676	11	<0.001

Model Summary

Step	-2 Log likelihood	Cox & Snell R Square	Nagelkerke R Square
1	126.518[a]	0.436	0.586

a. Estimation terminated at iteration number 6 because parameter estimates changed by less than 0.001.

Variables in the Equation

		B	S.E.	Wald	df	Sig.	Exp(B)	95% C.I.for EXP(B) Lower	Upper
Step 1[a]	age	0.026	0.017	2.289	1	0.130	1.027	0.992	1.062
	Sex	-0.117	0.530	0.048	1	0.826	0.890	0.315	2.517
	White	0.376	0.595	0.399	1	0.528	1.456	0.453	4.676
	Hispanic	-0.041	0.829	0.002	1	0.960	0.960	0.189	4.876
	Access	**0.904**	0.330	7.491	1	0.006	2.468	1.292	4.714
	Status_Quo	-0.096	0.372	0.066	1	0.797	0.909	0.438	1.884
	GistPrinciples	**1.225**	0.457	7.176	1	0.007	3.403	1.389	8.338
	GlobalBenefits	**0.756**	0.359	4.441	1	0.035	2.131	1.054	4.306
	GlobalRisks	-0.343	0.366	0.880	1	0.348	0.710	0.347	1.453
	QuantRisk	0.022	0.023	0.890	1	0.345	1.022	0.977	1.069
	SNS	-0.345	0.346	0.998	1	0.318	0.708	0.359	1.394
	Constant	-10.543	3.475	9.206	1	0.002	0.000		

a. Variable(s) entered on step 1: SNS.

Materials

Vaccination Knowledge

1	Getting the vaccine protects people from the flu
2*	Getting the vaccine is a very time consuming activity
3*	Flu vaccines are expensive
4*	There are very few locations which have flu vaccines available for people
5*	There are often flu vaccine shortages
6*	Getting the flu vaccine would increase the number of physician visits each year for recipients
7*	The seasonal flu often turns into a pandemic
8	The flu season ranges from September to April
9	It is possible to get the flu more than once in one flu season
10	Flu vaccines are inexpensive
11	The flu vaccine saves recipients an average of $50 a year in health care costs
12	Getting the flu vaccine would reduce the number of physician visits each year for recipients
13	One way to REDUCE the risk of getting the flu is to get the flu vaccine each year
14	Cold medicine can be used to treat the symptoms of the flu
15*	Cold medicine can be used to treat the cause of the flu
16	The flu can be spread between people by touching an infected item
17	The seasonal flu is viral and has no cure
18	The elderly and children under 6 years are the most susceptible to the flu
19	Pregnant women who get the vaccine, indirectly protect their unborn fetus and infant from the flu
20*	You cannot get the flu more than once in one flu season
21*	You have a low risk of getting the flu if you avoid health centers such as hospitals or physicians' offices
22	Washing your hands frequently lowers your risk of getting the flu
23	The flu can be spread by sharing drinks or food with an infected person

24*	Drinking orange juice decreases your chances of getting the flu
25	The seasonal flu vaccine protects against a majority of flu strains for that flu season
26*	The flu vaccine is completely effective at preventing the flu
27*	Receiving a flu vaccine will increase my chances of getting the flu
28*	Receiving a flu vaccine will provide me with lifelong protection from the flu
29*	The artificial immunity received from the flu vaccine is less effective than naturally acquired immunity
30*	Receiving a flu vaccine will make me more susceptible to upper respiratory illnesses
31*	Receiving the flu vaccine will weaken my immune system
32*	The flu is like a cold so I do not need to receive the vaccine
33	The flu vaccine is most necessary for those with weakened immune systems, like the elderly and children under 6
34	Even if I do not get the vaccine, if enough people around me get it, I will be protected by "herd immunity"
35*	Death is a common side effect of the flu vaccine
36*	Getting the vaccine will weaken my immune system and make me more susceptible to other illnesses
37*	Young adults do not need the flu vaccine, as they are not at risk from suffering from complications from the flu
38*	The flu vaccine has toxic metals and can cause people to develop mental disabilities such as autism
39*	The flu vaccine is introducing a virus into my system, and so it makes sense to avoid it, especially if I am already sick or pregnant
40*	Getting the flu vaccine will prevent my body from creating natural immune defenses
41*	Getting the flu vaccine will introduce the virus into my system, thus allowing me to spread it to others
42*	The flu vaccine contains toxic metals which can lead to or exacerbate the effects of progressive degenerative diseases such as Multiple Sclerosis.
43*	Getting the flu vaccine would increase my chances of getting sick with the flu
44*	Getting the flu vaccine would increase my chances of getting sick with a fever
45*	Getting the flu vaccine would decrease my productivity and my ability to work effectively
46*	Your chances of getting the flu are influenced by your behavior, such as going out with wet hair
47*	To treat the flu, I should take antibiotics
48*	If I were pregnant, it would be placing my unborn baby at risk if I were to get the flu vaccine
49*	Getting the flu is an inevitable part of life

50*	Getting the flu is the same as getting a cold
51	Getting the flu is the not same as getting a cold

Response scale: 1 = False, 2 = Probably False, 3 = Could Either be True or False, 4 = Probably True, 5 = True.
*Item is reversed scored.

Accessibility

1*	I would find it difficult to obtain a flu vaccine.
2*	I would find it difficult to afford a flu vaccine this flu season.
3*	I would find it difficult to afford a flu vaccine this flu season.
4*	I would find it difficult to obtain a flu vaccine this flu season.
5*	I am not sure I could obtain a flu vaccine this flu season.

Response scale: 1 = Completely Disagree to 5 = Completely Agree.
*Item is reversed scored.

Status Quo Gist

1	I feel fine now, so it is unnecessary to get the flu vaccine
2	I feel fine now, so there is no reason to be screened for a disease
3	I feel fine now, so there is no reason to get the flu vaccine

Response scale: 1 = Completely disagree, 5 = Completely agree

Gist Principles of Social Responsibility

1	Young adults have a responsibility to their families to get the flu vaccine
2	People who get the flu vaccine are protecting at-risk populations
3	My community would really benefit from a flu vaccine
4	Getting the flu vaccine protects myself and my loved ones from the flu
5	You should not hurt other people by giving them the flu
6	I have a responsibility to myself to get the flu vaccine
7	I have a responsibility to my family to get the flu vaccine
8	I have a responsibility to my child to not put him/her at risk
9	I have a responsibility to my elderly family members to not put them at risk

10	Getting vaccinated benefits my society.
11	Getting vaccinated protects those around me.

Response scale: 1 = Completely Disagree to 5 = Completely Agree

Global Risk and Global Benefit

Global Benefit	Overall, for YOU which of the following best represents the BENEFITS of getting a flu vaccine?
Global Risk	Overall, for YOU which of the following best represents the RISKS of getting a flu vaccine?

Response scale: 1 = None, 2 = Low, 3 = Medium, 4 = High

Quantitative Risk

1	Probability of getting the flu if you do get a flu vaccine?
2	Probability of death if you do get a flu vaccine?
3	Probability of getting sick if you do get a flu vaccine?
4	Probability of getting pneumonia if you do get a flu vaccine?
5	Probability of getting an upper respiratory illness if you do get the flu vaccine?
6	Probability of having a negative reaction to the flu vaccine?
7	Probability of having a mild side effect from the flu vaccine?
8	Probability of dying from the flu vaccine?
9	Probability of an allergic reaction to the flu vaccine?
10	Probability of an allergic reaction and getting sick if I get the flu vaccine?
11	Probability of a negative reaction and getting the flu if I get the flu vaccine?

Response scale: 0% no risk to 100% complete risk.

Social Norms

Friend Injunctive	Most of my friends believe.....People should get a flu vaccine[†]
Adult Injunctive	Most adults who are important to me believe....People should get a flu vaccine[†]
Friend Descriptive	Most of my friends....Have gotten the flu vaccination[‡]
Adult Descriptive	Most of these adults....Have gotten the flu vaccination[‡]

[†]Response scale: 1 = Completely Disagree to 5 = Completely Agree

‡Response scale: 0 = No and I don't know, 1 = Yes

Intentions to Get Vaccinated

1	Do you intend to get a flu vaccine at the start of the next flu season?
2	Do you think you will actually get a flu vaccine during the next flu season?
3	Do you intend to get a flu vaccine this flu season?
4	Do you think you will get a flu vaccine at the start of every flu season?
5	Do you plan to get a flu vaccine at the start of most flu seasons?
6	Do you think you will get the flu vaccine at least once in your lifetime?

Response scale: 1 = Extremely Unlikely, 2 = Unlikely, 3 = Undecided, 4 = Likely, 5 = Extremely Likely
